# Interviewer effects on abortion reporting: a multilevel analysis of household survey responses in Côte d’Ivoire, Nigeria and Rajasthan, India

**DOI:** 10.1136/bmjopen-2020-047570

**Published:** 2021-11-18

**Authors:** Katy Footman

**Affiliations:** Social Policy, The London School of Economics and Political Science, London, UK

**Keywords:** demography, statistics & research methods, reproductive medicine, public health

## Abstract

**Objectives:**

The analysis aimed to assess the scale of interviewer effects on abortion survey responses, to compare interviewer effects between different question wordings and between direct and indirect approaches, and to identify interviewer and interview characteristics that explain interviewer effects on abortion reporting.

**Setting:**

2018 Performance Monitoring for Action nationally representative household surveys from Côte d’Ivoire, Nigeria and Rajasthan, India.

**Participants:**

Survey data from 20 016 interviews with reproductive age (15–49) women, selected using multistage stratified cluster sampling. Data from self-administered interviewer surveys and from a sample of health service delivery points that serve the female survey participants were also included.

**Primary outcome measures:**

Outcomes were the respondent’s own experience of ever ‘removing a pregnancy’, their closest confidante’s experience of pregnancy removal and the respondent’s own experience of period regulation.

**Results:**

Substantial interviewer effects were observed, ranging from 7% in Côte d’Ivoire to 24% in Nigeria for pregnancy removal. Interviewer effects for survey questions that were designed to ask about abortion in a less stigmatising way were either similar to (9%–26% for confidante-reporting) or higher than (17%–32% for a question about period regulation) the pregnancy removal question. Interviewer and interview characteristics associated with abortion reporting included respondent–interviewer familiarity, the language of interview and the interviewer’s comfort asking questions about abortion.

**Conclusion:**

This study highlights that questions designed to be less stigmatising may increase interviewer effects due to lower comprehension among respondents. Further work is needed to assess question wordings for different contexts. Selecting and training interviewers to ensure comfort asking questions about abortion is important for reproductive health surveys. Challenges for the use of ‘insider’ interviewers and the management of surveys in countries with high linguistic diversity are also identified.

**Strengths and limitations of this study**

- This is one of the first studies of interviewer effects on abortion reporting in low- and middle-income countries and the first to assess interviewer effects in the recently developed Performance Monitoring for Action (PMA) survey abortion module.
- The analysis extends existing knowledge about interviewer effects for abortion as the PMA module included new survey questions, and meta-data allowed interview and interviewer characteristics to be assessed, including respondent–interviewer familiarity.
- It was not possible to entirely separate interviewer effects from area affects, so interviewer effects may be overestimated, but fixed respondent and community characteristics were included in the analysis to account for area effects to some extent.
- It is not possible to tell whether characteristics associated with higher reporting reflect an association with prior abortion experience or with willingness to report an abortion.

## Introduction

Only 37% of women of reproductive age live in countries where their legal right to access abortion does not depend on their reason for ending the pregnancy.^1^ Almost half of the 73 million abortions that occur each year are unsafe due to these restrictions on access to safe abortion.^2^ Unsafe abortions are defined by the WHO as abortions carried out either by a person lacking the necessary skills or in an environment that does not conform to minimal medical standards, or both.^3^ Despite its importance as a human rights and public health issue, there is limited evidence about abortion in most countries.^4^ Abortion data are needed to inform policy and advocacy: evidence about the incidence and impacts of unsafe abortion has played an important role in the liberalisation of abortion laws.^5–7^ Strategies to improve equitable access to safe abortion also require high-quality data about where and how women are seeking abortion, and which population subgroups are most affected by barriers to safe abortion care.^8 9^

Unsafe abortion disproportionately affects women in low-income and middle-income countries (LMICs) (86% of all abortions^10^ and 97% of unsafe abortions^1^), largely driven by laws and policies that restrict access to safe abortion. Data about abortion are often particularly limited in these contexts because health records exclude illegal, informal and self-managed abortions.^11^ Interviewer-administered household surveys tend to be key sources of demographic, reproductive and public health data in LMICs.^12–14^ However, concerns about under-reporting mean that survey data about abortion are rarely used and poorly trusted:^4 15^ studies from high-income countries that compare survey self-reports to health records suggest that 35%–80% of respondents accurately report abortions in surveys.^16–19^

Efforts to improve the availability of abortion data have focused on estimating abortion incidence using indirect methods^4^ or reducing under-reporting in surveys. Some surveys have (unsuccessfully) attempted to reduce under-reporting in direct questions by grouping abortions and miscarriages together,^4^ adding a filter question about previous unwanted pregnancies,^20^ or asking for a full pregnancy history.^21 22^ A recent review^4^ recommended additionally asking women directly if they have used medications to bring on their periods, due to recent changes in the way abortion methods are used and conceptualised. Performance Monitoring for Action (PMA) surveys now include this approach by asking participants about experiences of ‘period regulation’.^23^ Methods to reduce under-reporting in surveys through indirect questions have produced mixed results^4 18 24^ and have a number of flaws that limit the utility of the data they produce. For example, random response methods^25–27^ and list experiments^28–33^ do not produce individual-level data, have limited precision, and do not permit follow-on questions about sources of abortion and their safety, the process of abortion-seeking or issues with access to abortion care. Confidante-reporting,^34–36^ where the respondent is asked about abortions in their social networks, rather than their own abortions, has also been used in PMA surveys.^11^ Confidante-reporting can reduce the role of stigma in abortion reporting, though it relies on respondents being aware of their friends’ abortions and being willing to report them.^4^ Finally, survey researchers have also attempted to reduce under-reporting by limiting the impact of the interviewer through self-administered surveys,^37 38^ phone interviews^39 40^ or audio computer-assisted self-interviewing.^24 26^ However, these methods can be less feasible in contexts where there is lower literacy, technology use and phone ownership.^4 41^ Interviewers, therefore, continue to play a pivotal role in the quality of survey data, particularly in low-resource settings: they make contact with respondents, explain the purpose of the survey, gain consent, ask questions, record answers and motivate respondents throughout the interview.^42^

Abortion questions have often been excluded from household surveys for these methodological (and some political) reasons,^8^ but there are increasing efforts to address this gap. For example, several countries now include direct questions about abortion in Demographic and Health Surveys (DHS),^43 44^ and an abortion module was recently included in three countries’ PMA surveys.^45^ These surveys offer an opportunity to expand the availability of abortion data. However, more work is needed to explore data quality issues beyond under-reporting, to assess which questions and methods can improve the quality of abortion data from household surveys, and to identify which interviewer and interview characteristics affect abortion reporting.

Interviewers can impact the accuracy of survey responses due to their observed characteristics, such as age or gender, or role-related characteristics, such as the way they read questions, probe or provide instructions.^46^ Characteristics of the interaction itself can also impact survey responses, for example, whether there is pre-existing familiarity between interviewer and respondent,^47 48^ and the effects of observed interviewer characteristics (such as ethnicity or age) can be moderated by characteristics of the respondent.^49^ However, the characteristics of interviewers and interviews that may impact reporting of abortion in surveys has not previously been explored.

Interviewer effects vary by survey question, but are more likely to cause measurement error for questions that are complex, sensitive, non-factual, open-ended or evoke emotional responses.^50–52^ The interviewer effect on the variance of the mean for a survey item can be expressed as 1+ρ_int_(m-1), where m is the average number of interviews completed per interviewer and the interviewer effect ρ_int_ is the intraclass correlation or intrainterviewer correlation (IIC) of the survey responses.^53^ Interviewer effects can range from 1% to 12% of the variance in survey responses in face-to-face surveys, with most being below 2%,^54 55^ but even small interviewer effects can have a significant impact on survey data quality, especially if each interviewer collects a large number of responses.^56^

There is a large body of research on interviewer effects in high-income countries^49^ but few studies in LMICs,^41^ despite the increased importance of this topic in these contexts given higher dependence on interviewers for data collection.^12 48^ There have also been very few studies on interviewer effects for reproductive health surveys and this evidence has been mixed. A review of the DHS in Indonesia and the Philippines found low (1%) interviewer effects for questions about contraceptive use^57^ while analysis of the DHS in Kenya and Malawi found higher interviewer effects for contraceptive use (ranging between 3% and 25%), with interviewer gender, marital and fertility status being important covariates of response patterns.^58^ The remaining limited literature on interviewer effects in reproductive health surveys in LMICs has focused on the gender of interviewer^59–62^ and respondent-interviewer familiarity.^13 14 48 63–65^ Only one study has assessed interviewer effects on survey questions about abortion in LMICs, using a cross-classified multilevel model to separate interviewer effects and area effects in 22 DHS surveys. The study found large interviewer effects ranging from 0.2% to 50%, that interviewer effects were stronger for questions about ‘abortion’ than ‘any termination’, and that interviewer effects were greater than area effects.^41^ However, the interviewer and interview characteristics that might explain these effects could not be assessed in this DHS analysis, due to a lack of meta-data about interviewer and interview characteristics in most DHS surveys.

Building on these findings,^41^ the present analysis is the first to assess interviewer effects for abortion responses in PMA survey data. PMA surveys used an extended abortion module to collect nationally and state-representative data about abortion incidence and safety in 2018 in Nigeria, Côte d’Ivoire, and the state of Rajasthan in India, and expanded on previous survey methodologies for abortion by including new questions designed to increase reporting.^11 23 45^ This analysis, therefore, extends the limited literature on interviewer effects for abortion by assessing and comparing these new survey questions. In addition, PMA surveys include meta-data about interviewer and interview characteristics, so this analysis is the first to assess the characteristics that may explain interviewer effects on abortion questions. PMA surveys also collect abortion service delivery data from the area, allowing abortion reporting to be separated from abortion incidence to some extent in this analysis. Finally, the PMA model of using local interviewers enables further exploration of the role of respondent–interviewer familiarity in the context of interviewer effects for abortion.

### Aim

This analysis was intended to assess the scale of interviewer effects on abortion survey responses using PMA data. The aims of the analysis were to compare interviewer effects for abortion reporting: (1) between questions that employ different language to refer to abortion; (2) between direct and indirect approaches to abortion measurement and (3) with questions about less stigmatised topics. Finally, the analysis aimed (4) to identify interviewer and interview characteristics that explain interviewer effects on abortion reporting.

The study hypotheses were that questions about less stigmatised topics will have lower interviewer effects than questions about abortion, that questions designed to ask about abortion in a less stigmatising way (indirectly and using different language to refer to abortion) will have lower interviewer effects and that interviewer effects will be explained by interviewer and interview characteristics, including observable interviewer characteristics, interviewer–respondent familiarity and language of interview.

## Methods

### Contexts

This analysis used nationally representative PMA survey data collected in Nigeria,^66 67^ Côte d’Ivoire^68 69^ and Rajasthan state, India^70 71^ in 2018, as these surveys included the extended abortion module. The three countries have varying abortion laws, with abortion being broadly legal in India since 1971, while abortion is allowed only to save a woman’s life in Côte d’Ivoire and in Nigeria at the federal level. Abortion stigma is prevalent in each of the three contexts, though it can be more evident in countries with more restrictive abortion laws.^72^ The proportion of abortions that are most unsafe (involving non-recommended methods and non-clinical providers) was estimated at 62% in Côte d’Ivoire, 63% in Nigeria and 31% in Rajasthan by PMA.^45^ The most recent indirect estimates suggest the majority (73%) of abortions in India are medication abortions occurring outside health facilities, while only 5% of abortions involve informal unsafe methods.^73^ In Nigeria, use of medication abortions from pharmacies is also increasingly common,^74 75^ but in both the West African countries, unsafe methods are still most commonly used to end a pregnancy.^76–78^

### Data

PMA is a multicountry project that conducts frequent reproductive health surveys in nine countries in sub-Saharan Africa and Asia. PMA survey methodology is described in detail elsewhere.^79^ In brief, the surveys sample reproductive age women, households and health service delivery points (SDPs) using multistage stratified cluster sampling. Primary sampling units (PSUs) are selected using probability proportional to size procedures, with stratification by urban–rural status (in Côte d’Ivoire and Rajasthan) or state (Nigeria). In each PSU, households are mapped and listed by interviewers and 35 (40 in Lagos state, Nigeria) households are randomly selected to be invited for a face-to-face interview. Interviewers conduct the informed consent process. The proportion of selected, occupied households that consented to be take part in a household-level interview was 98% and the proportion of females within participating households who consented to take part was 98%–99% in each country.

A household interview is conducted, and all females age 15–49 in each household are interviewed using a structured questionnaire that includes demographic characteristics; pregnancy and fertility preferences; contraception; sexual activity; and menstrual hygiene. In 2018, researchers added an abortion module in round 2 in the survey of Côte d’Ivoire, round 5 of Nigeria and round 4 of Rajasthan, India. A survey is also conducted at a sample of health SDPs that serve the representative sample of reproductive age women. The SDP sample includes the public facilities at each level of the healthcare system that serve each PSU, and up to three private SDPs located in each PSU. The SDP questionnaire collects data on the availability and volumes of a range of health services, including abortion and postabortion care for the round of data collection under analysis. Additionally, before data collection begins, interviewers self-administer a staff survey with questions about their demographic characteristics and previous experience.

PMA surveys are repeated every 6–12 months with a new sample drawn at each round, so frequent, rapid data collection is facilitated using mobile data collection and ‘resident enumerators’ who live in the enumeration area. Interviewers may therefore be known or familiar to respondents, particularly as they are retained between data collection rounds whenever possible.^14^ Interviewers are typically women, over the age of 21, holding at least secondary school education, familiar with mobile phones and with no affiliation to the local health system.^14^

### Measures

The abortion module used four questions to gather data on experiences of abortion. First, the confidante method was used, where women are asked to report the abortions of a set number of close friends, defined by PMA as ‘women whom you share secrets with and who also share theirs with you’. In this survey, respondents were asked about their two closest friends but only data for the participant’s closest confidante were used in this analysis. Participants were asked whether each of their confidantes had ever done something to (1) ‘remove a pregnancy’ and/or to (2) ‘regulate a period’ when she was pregnant or worried she was pregnant. Participants could answer ‘yes I am certain’, ‘yes I think so’, ‘no’, ‘don’t know’ or ‘no response’, and the variable was dichotomised by grouping positive responses and treating ‘no response’ as missing (<1% of responses). Respondents were then asked whether they themselves had done something to (1) ‘remove a pregnancy’ and/or (2) ‘regulate a period’ when they were pregnant or worried that they were pregnant. Respondents could answer ‘yes’, ‘no’ or ‘no response’ and non-responses were treated as missing (<1% of responses). For each question, the interviewer was instructed to ‘probe to confirm whether the pregnancy removal (or period regulation) was successful’ and unsuccessful attempts were not recorded.

In this analysis, the respondent’s own experience of ever ‘removing a pregnancy’ was considered the main measure of a previous abortion. Interviewer effects for this outcome were compared with the abortion questions designed to be less stigmatising as they avoid personal disclosure^11^ or allow for different understandings of abortion^4 80 81^: their closest confidante’s experience of pregnancy removal and the respondent’s own experience of period regulation. The interviewer effects for these abortion questions were then compared with topics considered to be less stigmatised: first, whether the respondent or their partner were ‘currently doing something or using any method to delay or avoid getting pregnant’ (their current contraceptive use); second, whether the respondent reported they had ever given birth, and; third, whether the respondent reported they were currently pregnant.

Explanatory variables were selected based on previous literature about factors associated with abortion reporting,^17 19 38 39 82^ contraceptive use reporting^63–65^ and abortion incidence^10 17 19^ as well as the researchers’ hypotheses about the potential impacts of interviewer and interview characteristics on abortion reporting. Explanatory variables included respondent, community, interview and interviewer characteristics (see online supplemental appendix 1 for further detail). At the respondent level, explanatory variables included respondent age, education status, marital status, parity (ever given birth and number of births), household wealth and previous PMA participation. At the community level, explanatory variables were derived from the individual, household and service delivery data, including urban/rural status, region or state, whether the enumeration area had an SDP that provided abortion or post-abortion care, and the monthly volume of abortion services reported by these SDPs. Interview characteristics included respondent-interviewer familiarity (as recorded by the interviewer for each respondent) and survey language. Interviewer characteristics included age, marital status, education status, whether they had children, previous involvement in PMA data collection, previous survey experience outside of PMA, number of respondents interviewed and self-reported comfort asking questions about abortion. Comfort asking about abortion was measured using the question: ‘Are you comfortable asking respondents questions about abortion?’ with response options of ‘Yes, completely’, ‘Somewhat’ and ‘No’. Due to high levels of reported comfort asking questions about abortion, interviewers were considered to be comfortable only if they answered ‘Yes, completely’ as detailed in online supplemental appendix 1.

10.1136/bmjopen-2020-047570.supp1 Supplementary data

The staff survey was not completed in Nigeria in 2018 so interviewer characteristics were not available for all three countries. However, Nigeria was included in the analysis because the available data could still be used to address the first three aims of the analysis, and interview characteristics data were also available.

### Analysis

Descriptive analysis of the outcomes of interest and the potential explanatory variables was conducted to assess their distributions. Bivariate logistic regression analysis was used to assess associations between potential explanatory variables and the odds of reporting removing a pregnancy, period regulation and a confidante’s pregnancy removal.

Multilevel logistic regression models with an interviewer random intercept were then sequentially developed to assess the variance in outcomes within interviewers and between interviewers. Multilevel modelling was used to account for unmeasured interviewer characteristics and non-random allocation of respondents to interviewers, and to address the correlated error terms resulting from multiple respondents being interviewed by the same person and living within the same community (as there is one interviewer per PSU in PMA surveys).^56^ Explanatory variables were added to the model sequentially: Model 0 included only the interviewer random effects term, model 1 also included respondent and community characteristics, and model 2 additionally included interview and interviewer characteristics.

Model 0 was estimated according to the equation: log(π_ij_ /1-π_ij_) = β_0_+u_j_ where π is the probability of reporting having ever removed a pregnancy; β_0_ is the log-odds of reporting having removed a pregnancy when u=0 (ie, for the average interviewer); and the addition of u_j_ gives the intercept for interviewer j, or the interviewer effect. Models 1 and 2 were estimated using the equation: log(π_ij_ /1-π_ij_)= β_0_ + β_1_x_1ij_ + β_2_x_2ij_ + _…_+ β_k_x_kij_ + u_j_ where β_0_ is the log-odds of reporting having removed a pregnancy when x=0 and u=0; β_1_x_ij_ is the effect of a 1-unit change in x on the log-odds, holding constant all other explanatory variables and the interviewer effect u; and u_j_ is the interviewer effect for interviewer j. The models assume that (u_j_ ~N(0,σ^2^_u_)), meaning the level 2 residuals u_j_ are assumed to be independent and to follow normal distributions with means of zero. The variance of u_j_ (σ^2^_u_) is the level 2 residual variance and was used to calculate the IIC using the equation: IIC = σ_u_^2^ / σ_u_^2^+ σ_e_^2^ where σ_e_^2^ (the level 1 residual variance) is equal to 3.29 for a logit model.^83^ The IIC is therefore the proportion of total variance that can be explained by interviewer effects, and the terms ‘IIC’ and ‘interviewer effects’ are used interchangeably in the Results section.

Analyses were conducted for each country individually. Likelihood ratio tests were used at each stage to assess whether the multilevel model offered a significant improvement on a simple logistic model, and to assess whether the addition of variables improved the fit of the multilevel model. Simple fixed effects logistic regression models were also run at each stage and coefficients were compared between the simple and multilevel models to check for any substantial differences. In descriptive and bivariate analyses, survey weights were used and the complex sampling design was accounted for using the Taylor linearisation method. In the multilevel models, clustering and stratification were adjusted for through the inclusion of the interviewer random intercept and inclusion of strata (urban/rural and state) as covariates. The multilevel analysis was not weighted for simplicity, but the weights variable was included as a coefficient in model 2 as a sensitivity analysis. All other coefficients remained similar to the original model and the weight variable was non-significant in each country. The estimation procedure was maximum likelihood estimation using adaptive quadrature with seven integration points. To assess that seven integration points was adequate, the model was refitted with a larger number of integration points (up to 40) to assess that model parameters were substantially similar,^83^ and this sensitivity analysis confirmed that the coefficients were stable. Complete records analysis was conducted.

All analyses were conducted in Stata V.15.1. Statistical significance was determined using an alpha of 0.05, but coefficients significant at the 0.10 level were also noted.

### Patient and public involvement

No patients were involved in the development of the research questions, analysis or dissemination.

## Results

The interview and interviewer characteristics are shown in table 1 (respondent and community characteristics are in online supplemental appendix 2). There were 73 interviewers in Côte D’Ivoire, 145 in Rajasthan and 285 in Nigeria. Interviewer survey data were only available in Côte d’Ivoire and Rajasthan and in both surveys, all interviewers were female. Interviewers were older in Côte d’Ivoire and a lower proportion were married (20%) than in Rajasthan (80%). More interviewers in Côte d’Ivoire had technical or graduate education (86% vs 51%) and had previous survey experience (90% vs 19%). Almost all interviewers reported that they felt completely comfortable asking questions about abortion in both countries. There were no significant differences in the characteristics of interviewers who reported being completely comfortable asking about abortion in Côte D’Ivoire but in Rajasthan interviewers with technical or graduate education (94% vs 100%, p=0.049) and interviewers without children (92% vs 100%, p=0.007) were slightly less likely to report being completely comfortable compared with interviews with only primary/secondary education or with children (data not shown).

**Table 1:** Characteristics of interviewers and interview (weighted), by country

Interviewer characteristics			N	%	N	%	N	%
			Côte d'ivoire (n=73)		Rajasthan, India (n=145)		Nigeria (n=283)	
Mean respondents per interviewer			41		43		43	
Sociodemographics								
Mean age (SE)			31.1 (0.63)		26.9 (0.55)			
Female			70	100	134	100		
Married			14	20	107	80		
Has children			43	61	86	64		
Education								
Primary or secondary			10	14	65	49		
Technical or graduate			60	86	69	51		
Experience								
Existing staff			68	97	111	83		
Previous survey experience			63	90	26	19		
Comfortable asking about abortion			66	94	130	97		
**Interview characteristics**			**Côte d'Ivoire (n=2798)**		**Rajasthan, India (n=5915)**		**Nigeria (n=11 303)**	
Interviewer–respondent familiarity								
Very or well acquainted			42	1	3282	53	1780	19
Not well acquainted			109	3	1892	33	3641	32
Not at all acquainted			2647	95	741	14	5882	49
Language of interview								
French	Hindi	Hausa	1977	71	5673	94	5494	48
Baoule	English	English	170	6	44	2	4350	37
Yacouba	Other	Igbo	69	3	198	4	850	8
Attie	–	Yoruba	29	1			159	2
Dioula	–	Pidgin	438	14			247	3
Lobi	–	Other	54	3			203	3
Other	–	–	61	2				

Interviewer survey data were not collected in Nigeria (n=283). Interviewer survey data were missing for three interviewers (123 respondents) in Côte D’Ivoire and 11 interviewers (384 respondents) in Rajasthan.

The prevalence of reporting a previous abortion for the respondent themselves or their closest confidante is presented in table 2. Self-reports of having ever removed a pregnancy were highest in Côte d’Ivoire (19%) and Nigeria (15%) compared with Rajasthan (7%). Confidante-reporting was higher than self-report in Rajasthan and Nigeria, but not in Côte d’Ivoire. Reports of ever having regulated a period were significantly lower than reports of ever having removed a pregnancy in each setting, both for self-reporting and for confidante-reporting.

**Table 2:** Reported abortions of respondents and their closest confidante by country, weighted

	Côte d'Ivoire			Rajasthan, India			Nigeria		
	N	%	95% CI	N	%	95% CI	N	%	95% CI
Self-reported									
Pregnancy removal	511	19	(16 to 22)	390	7	(6 to 8)	1392	15	(13 to 16)
Period regulation	222	7	(5 to 10)	109	2	(1 to 2)	679	7	(6 to 8)
Closest confidante									
Pregnancy removal	305	18	(14 to 21)	705	15	(12 to 17)	1120	20	(18 to 22)
Period regulation	161	8	(6 to 22)	294	6	(4 to 8)	556	9	(8 to 11)
Total n respondents	2795			5912			11 254		
Total n confidantes	1803			4983			5986		

For self-reported pregnancy removal, a small number of respondents were coded as −99 (no response) in each country: 3 (0.1%) in Côte D’Ivoire, 3 (0.05%) in Rajasthan, 49 (0.4%) in Nigeria. For self-reported period regulation, the number of non-responses were 2, 9 and 47, respectively.

A high proportion of respondents reported that they did not have any close female confidantes age 15–49 who they mutually shared very personal information with: 991 (35.5%) in Côte D’Ivoire, 893 (15.2%) in Rajasthan and 4984 (45.4%) in Nigeria, resulting in a smaller sample size for these questions.

**Figure 1:**
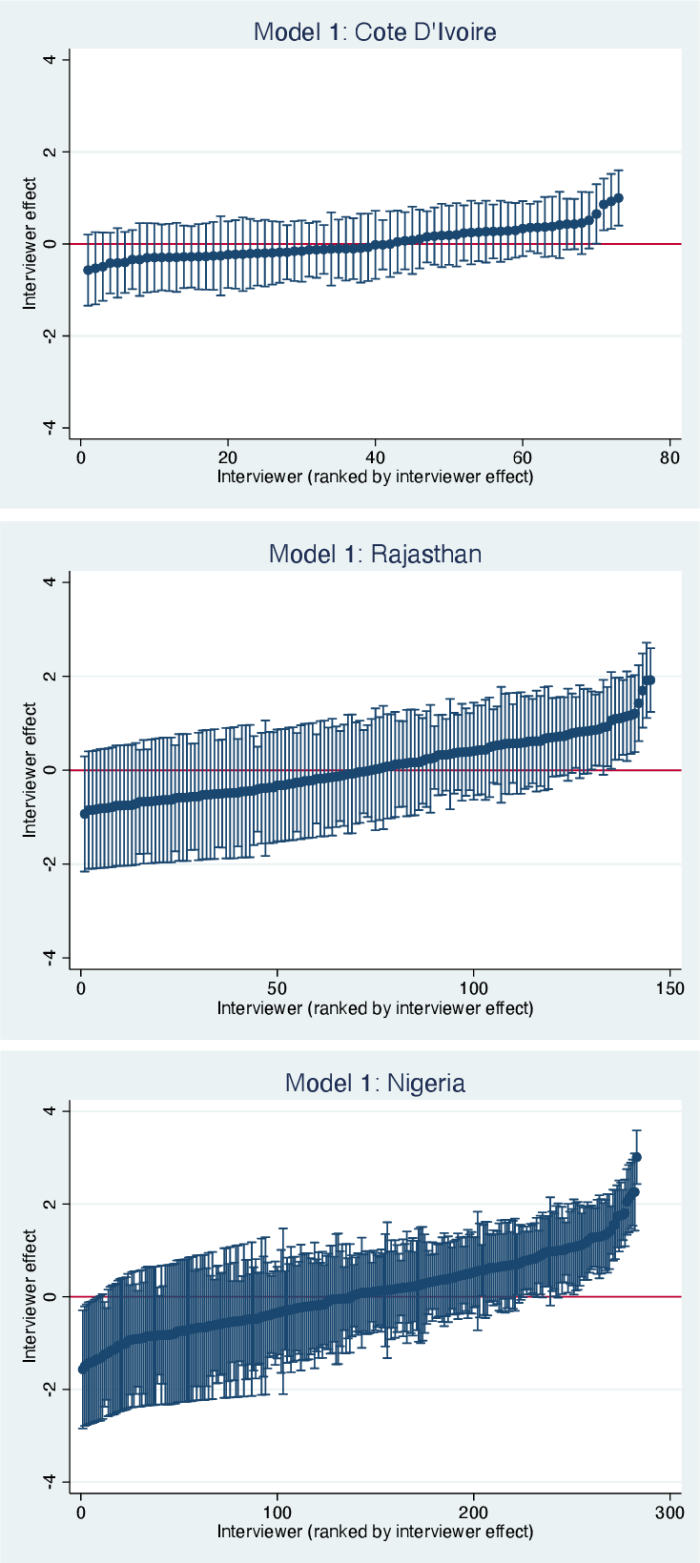
Caterpillar plots showing interviewer effects (level 2 residuals) with 95% CIs for the log-odds of reporting ever removing a pregnancy, adjusted for respondent and community characteristics.

**Figure 2:**
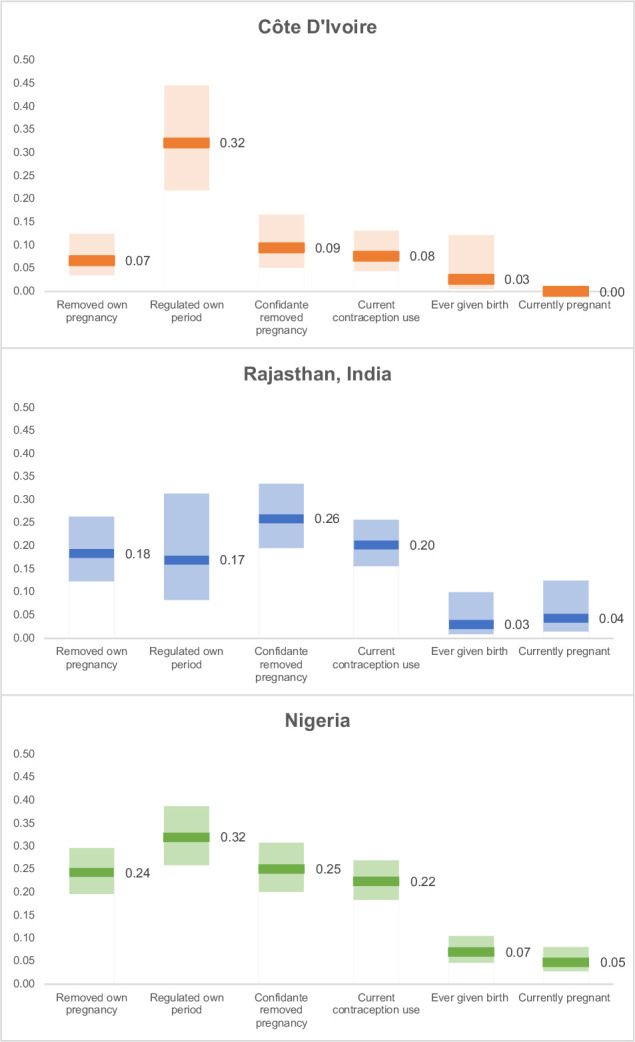
Intrainterviewer correlation by country and survey question, with 95% CIs, adjusted for respondent and community characteristics (model 1).

Figures 1 and 2 illustrate the interviewer effects in each country. The caterpillar plots in figure 1 show the mean log-odds for reporting ever removing a pregnancy to each interviewer, ordered by size, after adjusting for respondent and community characteristics (model 1). In figure 2, the interviewer effects for each of the questions is shown for each country (Model 1). Interviewer effects for the pregnancy removal question were substantial in each country but were largest in Nigeria and Rajasthan. Interviewer effect accounted for 7% of the variance in the odds of reporting removing a pregnancy in Côte d’Ivoire, 18% in Rajasthan and 24% in Nigeria. The interviewer effect was higher for the question about period regulation than pregnancy removal in the West African countries, particularly in Côte d’Ivoire (figure 2). The question about the closest confidante’s pregnancy removal had similar interviewer effects to the question about the respondent’s own pregnancy removal in each country. Interviewer effects were generally lower for the questions about previous births (ranging from 3% to 7%) and current pregnancy (0%–5%), compared with the questions about abortion. However, interviewer effects for the question about current contraceptive use were similar in size (8%–22%) to the questions about abortion.

Table 3 presents model 2 for the odds of reporting removing a pregnancy in each country, adjusting for respondent, community, interviewer and interview characteristics (full model is shown in online supplemental appendix 3). Interviewer effects were still substantial in model 2 for Rajasthan and Nigeria, suggesting that there are other factors not included in the model that may explain the variance between interviewers in these countries (particularly for Nigeria, where it was not possible to include interviewer characteristics in the model). The IIC for each of the models is presented in online supplemental appendix 4. Adjusting for interview and interviewer characteristics in model 2 did reduce the size of the interviewer effects for most countries and questions but did not significantly improve the fit of the model compared with model 1, particularly for Rajasthan.

**Table 3:** Full multilevel random intercept logit model (model 2) for the odds of reporting removing a pregnancy, adjusted for interviewer, interview, respondent and community characteristics*

			Côte d'Ivoire		Rajasthan, India		Nigeria	
			OR	(95% CI)	OR	(95% CI)	OR	(95% CI)
Interviewer characteristics								
No of respondents			1.00	(0.98 to 1.01)	0.99	(0.97 to 1.01)	**0.99**	**(0.98 to 1.00**)
Interviewer age			0.99	(0.96 to 1.02)	1.02	(0.99 to 1.06)		
Married (vs not married)			1.17	(0.84 to 1.64)	0.73	(0.39 to 1.34)		
Has children (vs no children)			***1.33***	***(0.96 to 1.83*** *)*	1.52	(0.85 to 2.74)		
Technical/Uni grad (vs secondary)			***1.47***	***(0.95 to 2.29*** *)*	1.08	(0.73 to 1.59)		
Existing staff (vs new staff)			1.34	(0.52 to 3.44)	1.11	(0.66 to 1.87)		
Previous survey experience (v none)			1.36	(0.82 to 2.26)	1.01	(0.62 to 1.64)		
Very comfortable asking about abortion (vs somewhat/not comfortable)			***1.88***	***(0.9 to 3.9*** *)*	1.55	(0.45 to 5.34)		
Interview characteristics								
Very well or well acquainted			Ref		Ref		Ref	
Not well acquainted			1.22	(0.28 to 5.22)	**0.68**	**(0.49 to 0.96**)	**0.75**	**(0.58 to 0.97**)
Not acquainted			1.67	(0.45 to 6.17)	0.91	(0.57 to 1.45)	0.85	(0.65 to 1.13)
Language of interview								
French	Hindi	Hausa	Ref		Ref		Ref	
Baoule	English	English	**3.11**	**(2.00 to 4.83**)	0.77	(0.08 to 6.97)	**1.59**	**(1.19 to 2.14**)
Yacouba	Other	Igbo	0.54	(0.15 to 1.93)	1.54	(0.69 to 3.47)	1.13	(0.72 to 1.78)
Attie	–	Yoruba	0.75	(0.28 to 2.02)			**1.94**	**(1.16 to 3.25**)
Dioula	–	Pidgin	**0.26**	**(0.14 to 0.48**)			**1.57**	**(0.99 to 2.47**)
Lobi	–	Other	***0.17***	***(0.02 to 1.35*** *)*			1.61	(0.73 to 3.55)
Other	–	–	0.67	(0.27 to 1.72)				
Intrainterviewer correlation			**0.01**		**0.15**		**0.22**	

Coefficients in bold are significant at the <0.05 level, coefficients in bold and italics are significant at the <0.10 level. Interviewer characteristics were unavailable for Nigeria.

*Respondent characteristics include age, age squared, education, marital status, ever given birth and number of birth events, wealth quintile and whether the respondent is a previous Performance Monitoring for Action (PMA) respondent. Community characteristics include region or state, rural/urban status, monthly number of abortions per community (mean, facility reported) and whether there is an abortion care facility in the community. The full model is available in online supplemental appendix 3, including all respondent and community variable coefficients.

In both Rajasthan and Nigeria, respondents who were not well acquainted with the interviewer had significantly lower odds of reporting a pregnancy removal compared with respondents who were well or very well acquainted. However, respondents who were not at all acquainted did not have significantly different odds from those who were well acquainted. There was no significant association with respondent-interviewer familiarity in Côte d’Ivoire, where almost all respondents were not at all familiar with the interviewer. Survey language was significantly associated with the odds of reporting a pregnancy removal in the West African countries, but not in Rajasthan where almost all interviews were conducted in Hindi.

In Rajasthan, none of the interviewer characteristics were significant. In Côte d’Ivoire the interviewer’s parity, education level and comfort asking questions about abortion were significant at the 0.1 level.

In each country (online supplemental appendix 3), respondent characteristics were significantly associated with the odds of reporting a pregnancy removal, in line with previous studies about subgroup differences in abortion reporting^17 19^ and abortion incidence.^10 17 19^ Broadly, respondents who were older, with more formal education and in a higher wealth quintile were more likely to report a pregnancy removal, though there were some differences between countries. Abortion service availability and volumes were only significant in Nigeria, where the availability of a clinic providing abortion was associated with significantly higher odds of reporting a pregnancy removal, though surprisingly the association with the facility-reported monthly number of abortions was negative.

## Discussion

Interviewer-administered household surveys will continue to be an important source of health information in LMICs for the foreseeable future due to limitations of formal health records^11^ and challenges with alternative modes of survey data collection in low-resource settings.^12^ A greater understanding of interviewer effects for abortion questions in household surveys can support efforts to improve the quality of these data and increase the availability of much-needed evidence about abortion.

This analysis identified substantial interviewer effects for abortion reporting in three PMA survey countries, ranging from 7% in Côte d’Ivoire to 24% in Nigeria for ever having removed a pregnancy. Contrary to the original study hypotheses, questions designed to ask about abortion in a less stigmatising way did not have lower interviewer effects, and in fact interviewer effects were higher for a question about period regulation than pregnancy removal in Côte d’Ivoire and Nigeria. Although interviewer effects were considerably smaller for less stigmatised topics such as previous births and current pregnancies as hypothesised, interviewer effects for contraceptive use were similar in scale to questions about abortion. The interviewer characteristics that affect abortion reporting could not be fully explained by the available data, but significant interview and interviewer characteristics identified in this analysis included: respondent–interviewer familiarity, the language of interview, and the interviewer’s comfort asking questions about abortion.

Interviewer effects observed in this study were higher than average estimates from surveys in high-income countries, which tend to be below 2% and vary from 1% to 12% (though these estimates are not for abortion measures).^54 55^ Higher variance between interviewers does not necessarily indicate lower validity of abortion reporting, as each interviewer could discourage true reporting equally, but evidence that less sensitive questions have lower interviewer variance suggests that lower variance may be a sign of higher validity.^41^ Interviewer effects can also affect survey data quality by increasing the variance of estimates,^56^ so understanding, minimising and accounting for interviewer effects is important.

Interviewer effects for questions about contraceptive use in PMA were as high as the abortion questions. The evidence on interviewer effects for contraceptive use questions in DHS data is limited and mixed^57 58^ but inaccuracies in self-reports of contraceptive use have been noted in several studies.^84–86^ Contraceptive use can be highly sensitive and is often covert.^87^ The topic of survey data quality for contraceptive use has been neglected,^84^ but the high interviewer effects for contraceptive use observed in this analysis suggest that this issue warrants further attention. Unlike abortion, contraceptive use is a widely collected survey item, and its measurement was the original aim of investment in PMA.^79^ Though abortion faces the additional issue of under-reporting in surveys, this finding does raise questions about the frequent exclusion of abortion from reproductive health surveys on methodological grounds, since some of the same quality issues exist for other common reproductive health topics.

To improve the quality of survey data on abortion, further assessment of the most effective survey questions is required. The questions used in the PMA surveys were the result of extensive pilot testing that aimed to appropriately capture the nuance of how women discuss and refer to abortion experiences.^88 89^ Although the PMA pilot in Côte d’Ivoire suggested high understanding of the period regulation question,^90^ the higher interviewer effects for period regulation observed in this study in both West African countries may have been caused by respondent confusion about the meaning of the question, resulting in the need for additional, unscripted clarification or explanation by the interviewers. Interviewer effects were similar for self-reporting and confidante-reporting, suggesting that this data quality issue is not reduced by using indirect reporting. Further cognitive interviewing and formative research could support identification of the most effective wording for asking about previous abortion experiences, and further work could also consider how ‘removing a pregnancy’ is understood compared with other commonly used terminologies. Use of familiar words is important for describing sensitive behaviours,^91^ but these are likely to vary considerably by context, creating challenges for cross-country comparisons. The finding that abortion reporting varied significantly based on the language of interview poses additional challenges for cross-national surveys, but also for countries where there are high levels of linguistic diversity. Understanding how the specific meanings and associations of question wording may vary between languages is also important during cognitive interviewing to assess potential impacts of specific wording on survey reporting.

The findings indicate that sociodemographic characteristics of interviewers were not significantly associated with respondents reporting a pregnancy removal, suggesting that the interview context or interviewer skills and behaviours may be more important than interviewers’ observable characteristics. Previous studies of interviewer effects have often found the predictive power of variables from interviewer surveys are low, explaining only a small proportion of observed variance, but interviewer surveys can be strengthened through inclusion of questions relating to attitudes, behaviours, experiences and expectations of survey outcomes.^42^ Use of interviewer observation or paradata on, for example, the speed of interview, may also provide further insight into the interviewer skills and behaviours that improve the validity of abortion reporting. Comfort asking questions about abortion was reportedly high but was significantly associated with abortion reporting at the <0.1 significance level in Côte d’Ivoire, suggesting additional values clarification training on abortion for interviewers may improve the quality of their data. Values clarification and attitude transformation workshops have been found to improve knowledge, attitudes and behavioural intentions relating to abortion and are intended to also reduce the impact of potential negative attitudes on professional responsibilities and ethics.^92^

In Rajasthan and Nigeria, respondent–interviewer familiarity was significantly associated with abortion reporting. Being slightly acquainted with an interviewer was negatively associated with abortion reporting compared with being well-acquainted, but there was no significant difference between reporting to a close acquaintance or a complete stranger. The historical norm of surveys has been to have strangers as interviewers, as respondents may wish to avoid judgement from a peer, or fear that their answers will not remain confidential. However, use of ‘insiders’ as interviewers may increase the interviewers’ understanding of local culture, increase the likelihood of being invited into a private space, and can promote rapport, trust and closeness which can increase the motivation to answer truthfully.^48 63 93^ The impact of interviewer–respondent familiarity will likely vary over time, and with the level of familiarity.^63^ In these PMA surveys, it seems that a loose tie between the interviewer and respondent may not be conducive to abortion reporting, which may relate to concerns about confidentiality and trust, compared with a stranger or a close acquaintance. This finding may support future use of stranger interviewers, as it is difficult to systematically ensure that acquainted interviewers are closely acquainted, rather than slightly acquainted, with all respondents. However, the results contrast with previous research that found limited difference in contraceptive reporting to local-insider and local-stranger interviewers in the Dominican Republic, while over-reporting was significantly higher to outsider-interviewers,^64^ suggesting that the benefits of personal and local familiarity may vary by context and topic.

### Strengths and limitations

This analysis has a number of limitations. First, the most significant limitation of this study was that it was not possible to entirely separate interviewer effects and area effects, as the survey does not have an interpenetrated design where respondents or geographic areas are randomly assigned to interviewers. Cross-classified multilevel models have been found to effectively estimate interviewer effects without a randomised design,^94^ but in this study cross-classified modelling could not be used to separate area and interviewer effects because one interviewer is assigned to each cluster in PMA surveys. This is problematic as respondents in different clusters may have different probabilities of reporting abortions due to geographic, cultural or demographic differences, and interviewer effects cannot be separated from area effects. However, studies have found area random effects to be non-significant after controlling for household fixed effects in cross-classified models,^95^ so controlling for the wide range of respondent-level and community-level fixed effects included in this analysis (demographic and socioeconomic characteristics of participants, region, urban/rural status, presence of abortion-providing facilities and number of abortions reported by these facilities) will account for area effects to some extent. Interviewer effects may be slightly over-estimated in this study since area effects may not be entirely explained by the respondent and community-level fixed effects. However, the only other analysis to have assessed interviewer effects for abortion reporting did use a cross-classified model to separate area and interviewer effects,^41^ and found interviewer effects were greater than area effects after controlling for region and rural/urban status, as done in this analysis.

Second, it is not possible to tell whether variables associated with higher abortion reports reflect an association with prior abortion experience or a willingness to report it. Though the inclusion of facility-reported data on number of abortions and abortion service availability was intended to partially account for this issue, there are likely inaccuracies in the facility reports of abortion caseload. Third, interviewer survey data were missing for 4% of respondents in Côte d’Ivoire and 6% of respondents in Rajasthan. However, sensitivity analysis was conducted to compare the coefficients and standard errors for model 1 when restricting the model to those cases that had complete interviewer survey data, and the results did not vary when comparing the model using all data to the model using only non-missing data. Fourth, for the sake of simplicity, the models do not include interactions between respondent and interviewer characteristics, which could be explored in future research. Fifth, while survey language was included as a potential explanatory variable in this analysis because linguistic differences in question wording was hypothesised to affect reporting due to potential differences in meanings and associations, the language spoken by respondents may reflect other respondent characteristics which are not fully accounted for in the model, such as religion and ethnicity (though region and education status are included). Finally, the limited variation in interviewer characteristics (table 1) and the limited number of questions relating to interviewer attitudes, behaviours, experiences and expectations in the staff survey may have reduced the predictive power of the variables included in this analysis.

This study also has several strengths. It is the first to assess interviewer effects in the expanded abortion module used by the PMA surveys in 2018, and it is one of the first to assess interviewer effects for abortion questions in household surveys in LMICs. The analysis used data collected from diverse settings in the same time period using standardised questionnaires and trainings. The data provide a rich range of respondent and community characteristics, including abortion service delivery environment data, as well as interviewer characteristics (in two countries) and interview characteristics. Finally, the PMA model of using local interviewers enabled further exploration of the role of respondent–interviewer familiarity in the context of interviewer effects for abortion.

## Conclusion

Surveys offer one of the only opportunities to gather representative evidence about the sources and safety of abortions, the subgroups most affected by unsafe abortion and their abortion-seeking pathways. These data are critical to inform strategies, policies and programmes, and will become even more important with the shift towards self-managed medication abortions using drugs purchased informally through pharmacies or online. Understanding interviewer effects for abortion reporting can help inform decisions about whether to include abortion questions in demographic and public health surveys, which questions to use, and how abortion survey data quality can be improved through methodological adjustments. This analysis highlights that interviewer effects for abortion reporting were high in the PMA surveys in Côte d’Ivoire, Nigeria and Rajasthan from 2018. Further work is needed to identify the effect of different question wordings on abortion reporting through comparative studies and more comprehensive qualitative and cognitive interviewing. Observable interviewer characteristics were not significantly associated with abortion reporting, suggesting skills and behaviours of interviewers may be responsible for unexplained variance at the interviewer level, and this could be further explored through the addition of relevant variables to the interviewer survey. Additional values clarification trainings or other mechanisms to address issues of abortion stigma and ensure interviewers feel comfortable asking questions about abortion may improve the quality of abortion data from surveys. Interview characteristics, including respondent–interviewer familiarity and language of interview, were also significantly associated with abortion reporting, which raises challenges for survey logistics when using ‘insider’ interviewers, for countries with high linguistic diversity and cross-national surveys. Consideration of variations in language should inform testing of different question wordings and designs in future work.

## Supplementary Material

Author's manuscript

## Data Availability

Data are available in a public, open access repository. PMA survey datasets can be requested through the PMA website: https://www.pmadata.org/data/available-datasets/request-access-datasets.
