# Supplementary material for: Interviewer effects on abortion reporting: a multilevel analysis of household survey responses in Côte d’Ivoire, Nigeria and Rajasthan, India
Source: BMJ Open. 2021 Nov 18;11(11):e047570. doi: 10.1136/bmjopen-2020-047570 (PMC8606767; doi:10.1136/bmjopen-2020-047570)
Supplement: Supplementary data [file bmjopen-2020-047570supp001.pdf]

Appendix 1. Details of explanatory variables

Variable	Question	Categories	Re-code
**Respondent-Level Variables**			
**Age**	How old were you at your last birthday? [confirmed agreement with birth date]	Integer	- Treated as continuous
**Education**	What is the highest level of school you attended? [Only record formal schooling]	Never attended<br>Primary<br>Secondary<br>Higher Secondary [India only]<br>Tertiary / Higher / Graduate<br>No response	- 'Higher secondary' grouped with 'Secondary'<br>- No response treated as missing
**Marital status**	Are you currently married or living together with a man as if married?<br>[Probe: If no, ask whether the respondent is divorced, separated, or widowed.]	Currently married<br>Currently married, gauna not performed [India only]<br>Living with a man<br>Divorced / separated<br>Widow<br>No, never in union<br>No response	- 'Married, gauna not performed' coded as 'Currently married'<br>- Divorced/separated grouped with Widowed<br>- No response treated as missing.
**Ever given birth**	Have you ever given birth?	Yes<br>No<br>No response	- No response treated as missing.
**Number of birth events**	How many times have you given birth?	Integer	- No response treated as missing
**Wealth**	Household wealth index questions administered in household questionnaire	Lowest quintile<br>Lower quintile<br>Middle quintile<br>Higher quintile<br>Highest quintile	- Pre-constructed in dataset
**Previous PMA participation**	Has the respondent previously participated in PMA 2020 surveys? [completed by interviewer]	Yes<br>No<br>Do not know<br>No response	- Do not know and No response coded as missing
**Community-Level Variables**			
**Region / State**	Enumeration area categorised based on sampling frame	Vary by country	- Pre-constructed in dataset
**Urban /rural**		Urban<br>Rural	- Pre-constructed in dataset
**Abortion care facility in community**	Does this facility provide post-abortion care for pregnancies of 12 weeks or less / more than 12 weeks?<br>AND<br>Does this facility provide abortion for pregnancies of 12 weeks or less / more than 12 weeks?	Yes<br>No<br>Do not know<br>No response	- Binary variable coded 1 if any facilities in or serving the enumeration area answered yes to providing abortion or post-abortion care for either gestational age group, and 0 if all facilities answered no to all questions. Public hospitals were excluded as hospital catchment areas can be very large, so an enumeration area may not have abortion care available if it only has a public hospital serving the area.
**Monthly mean number of abortions**	In an AVERAGE / the LAST month, how many post-abortion care patients would you estimate are/were treated as INPATIENTS / OUTPATIENTS in this facility as a whole? Please remember to include all patients	Integer variable<br>-88 for do not know<br>-99 for no response.	- Average of the number of post-abortion care services in the last month and in an average month was calculated for each facility (to get a more accurate average that accounts for possible seasonal variation).<br>- Average of the number of abortion services in the last month and in an average month was calculated for each facility (to get a

	treated for extremely serious or minor abortion complications, whether the abortions were spontaneous or induced. Ensure provider responds from memory and does NOT reference the log book.<br><br>In an AVERAGE / the LAST month, how many abortions are/were provided?		-<br><br>-	more accurate average that accounts for possible seasonal variation).<br>The average numbers of abortions and post-abortion care services were summed per facility, and then per cluster to give a cluster total of the number of abortions / post-abortion care services reported by facilities per month on average.
**Interview-level Variables**				
**Interviewer-respondent familiarity**	How well acquainted are you with the respondent? [completed by interviewer]	Very well acquainted<br>Well acquainted<br>Not well acquainted<br>Not acquainted	-	Very well acquainted and well acquainted grouped together to give a categorical variable with three groups.
**Survey language**	Administrative data recorded by interviewer	Varied by country	-	Languages used in <20 interviews grouped into ‘Other’
**Interviewer-level Variables**				
**Number of respondents**	-	-	-	Constructed by summing the number of interviews completed per interviewer
**Age**	How old were you at your last birthday?	Integer	-	Treated as continuous
**Marital status**	Select your marital status	Never Married<br>Currently Married<br>Living with a partner<br>Divorced<br>Widowed	-	Coded as binary variable where 1 is married (‘Currently Married’) and 0 is all other categories
**Children**	Do you have any children?	Yes<br>No	-	
**Education**	What is the highest level of education that you have completed?	Primary School<br>Secondary School<br>Higher Secondary [India only]<br>University<br>Technical School<br>Graduate School (Masters)<br>Graduate School (Doctoral) [Côte D’Ivoire only]	-	Recoded into a binary variable where 1 is "Primary/Secondary" and 2 is "Technical / Uni graduate and above"
**Existing staff**	Have you ever completed a round of data collection for PMA2020 before the round you are preparing for now?	Yes, I am a returning PMA2020 staff member<br>No, this will be my first round of data collection	-	
**Previous experience**	Do you have any previous survey experience?	Yes<br>No	-	
**Comfort asking about abortion**	Are you comfortable asking respondents questions about abortion?	Yes, completely<br>Somewhat<br>No	-	Binary variable coded as 1 if interviewer answered ‘yes completely’ and 0 if they answered ‘somewhat’ or ‘no’

Appendix 2. Characteristics of respondents and communities by country (weighted)

Respondent characteristics	Côte d'Ivoire (n=2798)		Rajasthan, India (n=5915)		Nigeria (n=11,303)	
	N	%	N	%	N	%
**Mean age** (SE)	28.5 (0.21)		29.1 (0.15)		29.1 (0.13)	
**Education**						
No formal schooling	1,282	45%	2,212	37%	2,389	18%
Primary school	733	26%	1,424	24%	1,931	15%
Secondary school	626	23%	1,509	26%	5,039	47%
Higher education	154	6%	770	14%	1,944	20%
**Marital status**						
Currently married	1,187	41%	4,547	77%	7,069	59%
Living with partner	622	24%	9	0%	414	5%
Divorced or widowed	129	4%	153	3%	528	5%
Never married	859	31%	1,188	20%	3,288	31%
**Parity**						
Ever given birth	2,080	74%	4,082	69%	7,456	65%
Mean number of births (SE)	2.5 (0.12)		1.9 (0.04)		2.3 (0.06)	
Previous PMA participant	387	23%	1,281	25%	1,884	23%
**Community characteristics**						
Urban	1,768	62%	1,503	35%	5,664	57%
Rural	1,096	38%	4,561	65%	5,875	43%
Abortion care facility in community	2,129	76%	1,624	27%	5,602	50%
Mean monthly facility-reported number of abortions, per community with abortion facility (SE)	2.7 (0.09)		12.7 (0.51)		5.9 (0.11)	

Abbreviations: SE = Standard Error.

Appendix 3: Full multilevel random intercept logit model (Model 2) for the odds of reporting removing a pregnancy, adjusted for interviewer, interview, respondent and community characteristics

	Côte d'Ivoire		Rajasthan, India		Nigeria	
	OR	[95% CI]	OR	[95% CI]	OR	[95% CI]
**Interviewer characteristics**						
Number of respondents	1.00	[0.98;1.01]	0.99	[0.97;1.01]	**0.99**	**[0.98;1.00]**
Interviewer age	0.99	[0.96;1.02]	1.02	[0.99;1.06]		
Married (vs not married)	1.17	[0.84;1.64]	0.73	[0.39;1.34]		
Has children (vs no children)	***1.33***	***[0.96;1.83]***	1.52	[0.85;2.74]		
Technical / Uni grad (vs secondary)	***1.47***	***[0.95;2.29]***	1.08	[0.73;1.59]		
Existing staff (vs new staff)	1.34	[0.52;3.44]	1.11	[0.66;1.87]		
Previous survey experience (v none)	1.36	[0.82;2.26]	1.01	[0.62;1.64]		
Very comfortable asking about abortion (vs somewhat/not comfortable)	***1.88***	***[0.9;3.9]***	1.55	[0.45;5.34]		
**Interview Characteristics**						
Very well or well acquainted	Ref		Ref		Ref	
Not well acquainted	1.22	[0.28;5.22]	**0.68**	**[0.49;0.96]**	**0.75**	**[0.58;0.97]**
Not acquainted	1.67	[0.45;6.17]	0.91	[0.57;1.45]	0.85	[0.65;1.13]
Language of interview						
French / Hindi / Hausa	Ref		Ref		Ref	
Baoule / English / English	**3.11**	**[2.00;4.83]**	0.77	[0.08;6.97]	**1.59**	**[1.19;2.14]**
Yacouba / Other / Igbo	0.54	[0.15;1.93]	1.54	[0.69;3.47]	1.13	[0.72;1.78]
Attie / - / Yoruba	0.75	[0.28;2.02]			**1.94**	**[1.16;3.25]**
Dioula / - / Pidgin	**0.26**	**[0.14;0.48]**			**1.57**	**[0.99;2.47]**
Lobi / - / Other	***0.17***	***[0.02;1.35]***			1.61	[0.73;3.55]
Other / - / -	0.67	[0.27;1.72]				
**Respondent Characteristics**						
Age	**1.35**	**[1.22;1.50]**	**1.40**	**[1.21;1.61]**	**1.46**	**[1.38;1.56]**
Age squared	**1.00**	**[0.99;1.00]**	**0.99**	**[0.99;1.00]**	**0.99**	**[0.99;1.00]**
No formal schooling	Ref		Ref		Ref	
Primary school	**1.96**	**[1.48;2.60]**	**2.01**	**[1.48;2.72]**	**1.62**	**[1.21;2.16]**
Secondary school	**2.10**	**[1.50;2.92]**	**1.46**	**[1.00;2.14]**	**2.19**	**[1.63;2.94]**
Higher education	1.31	[0.8;2.150]	1.49	[0.92;2.41]	**1.94**	**[1.39;2.7]**
Married	Ref		Ref		Ref	
Living with partner	**2.85**	**[2.12;3.83]**	1.33	[0.14;13.0]	**2.75**	**[2.07;3.65]**
Divorced or widowed	**2.25**	**[1.39;3.63]**	0.94	[0.47;1.89]	**1.44**	**[1.11;1.87]**
Never married	**3.66**	**[2.54;5.27]**	**0.14**	**[0.04;0.51]**	**1.30**	**[1.01;1.67]**
Ever given birth (vs never)	**3.63**	**[2.41;5.46]**	**2.76**	**[1.49;5.13]**	**1.39**	**[1.09;1.79]**
Number of birth events	**0.92**	**[0.85;1.00]**	1.08	[0.97;1.19]	***0.96***	***[0.92;1.01]***
Lowest wealth quintile	Ref		Ref		Ref	
Lower quintile	1.36	[0.90;2.05]	0.81	[0.51;1.29]	**1.44**	**[1.10;1.90]**
Middle quintile	1.23	[0.78;1.95]	1.06	[0.67;1.66]	***1.33***	***[0.96;1.84]***
Higher quintile	***1.61***	***[0.97;2.64]***	0.88	[0.54;1.43]	**1.50**	**[1.07;2.11]**
Highest quintile	**1.88**	**[1.12;3.15]**	1.07	[0.64;1.78]	**1.75**	**[1.22;2.52]**
Previous PMA respondent (vs not)	1.26	[0.90;1.75]	0.82	[0.62;1.10]	0.91	[0.74;1.10]
**Community Characteristics**						
Region or state	0.99	[0.97;1.01]	0.99	[0.97;1.01]	1.01	[0.94;1.08]
Rural (vs urban)	0.99	[0.68;1.46]	**0.57**	**[0.36;0.91]**	0.77	[0.56;1.05]
Monthly number of abortions per community (mean, facility-reported)	0.99	[0.95;1.04]	1.00	[0.97;1.02]	**0.97**	**[0.94;0.99]**
Abortion care facility in community (vs none)	0.75	[0.55;1.03]	1.42	[0.90;2.26]	**1.36**	**[1.04;1.78]**

**Intra-Interviewer Correlation**	**0.01**	**0.15**	**0.22**

Notes: Coefficients in bold are significant at the <0.05 level, coefficients in bold and italics are significant at the <0.10 level. Interviewer characteristics were unavailable for Nigeria. OR = odds ratio. CI = confidence intervals.

Appendix 4. Multilevel logit models with interviewer effects for PMA survey outcomes

	Côte d'Ivoire				Rajasthan, India				Nigeria			
	Level 2 variance	IIC	95% CI	LR test P value	Level 2 variance	IIC	95% CI	LR test P value	Level 2 variance	IIC	95% CI	LR test P value
**Abortion-related survey questions**												
**Ever removed a pregnancy**												
Null model	0.60	**0.15**	[0.10;0.23]		0.81	**0.20**	[0.14;0.28]		1.86	**0.36**	[0.31;0.42]	
Model 1	0.24	**0.07**	[0.03;0.13]	<0.001	0.74	**0.18**	[0.12;0.26]	<0.001	1.05	**0.24**	[0.20;0.30]	<0.001
Model 2	0.03	**0.01**	[0.00;0.11]	<0.001	0.60	**0.15**	[0.10;0.24]	0.15	0.94	**0.22**	[0.18;0.27]	<0.001
**Ever regulated a period**												
Null model	1.63	**0.33**	[0.23;0.45]		0.74	**0.18**	[0.10;0.32]		1.95	**0.37**	[0.31;0.44]	
Model 1	1.56	**0.32**	[0.22;0.45]	<0.001	0.67	**0.17**	[0.08;0.31]	<0.001	1.55	**0.32**	[0.26;0.39	<0.001
Model 2	1.10	**0.25**	[0.15;0.38]	0.03	0.45	**0.12**	[0.05;0.27]	0.08	1.51	**0.32**	[0.25;0.38]	0.30
**Confidante removed pregnancy**												
Null model	0.40	**0.11**	[0.06;0.18]		1.17	**0.26**	[0.20;0.34]		1.33	**0.29**	[0.24;0.35]	
Model 1	0.34	**0.09**	[0.05;0.17]	<0.001	1.15	**0.26**	[0.20;0.33]	<0.001	1.10	**0.25**	[0.20;0.31]	<0.001
Model 2	0.15	**0.04**	[0.01;0.12]	0.01	0.99	**0.23**	[0.17;0.31]	0.18	1.01	**0.23**	[0.19;0.29]	<0.001
**Less stigmatised survey questions**												
**Current contraceptive user**												
Null model	0.40	**0.11**	[0.07;0.17]		0.35	**0.10**	[0.07;0.13]		1.15	**0.26**	[0.22;0.31]	
Model 1	0.27	**0.08**	[0.04;0.13]	<0.001	0.83	**0.20**	[0.16;0.26]	<0.001	0.95	**0.22**	[0.18;0.27]	<0.001
Model 2	0.17	**0.05**	[0.02;0.10]	<0.001	0.73	**0.18**	[0.14;0.24]	0.07	0.92	**0.22**	[0.18;0.26]	0.07
**Ever given birth**												
Null model	0.43	**0.11**	[0.07;0.17]		0.06	**0.02**	[0.01;0.03]		0.37	**0.10**	[0.08;0.13]	
Model 1*	0.09	**0.03**	[0.01;0.12]	<0.001	0.10	**0.03**	[0.01;0.10]	<0.001	0.25	**0.07**	[0.05;0.11]	<0.001
Model 2	0.02	**0.01**	[0.00;0.00]	0.19	0.02	**0.00**	[0.00;0.85]	0.06	0.23	**0.06**	[0.04;0.10]	<0.001
**Currently pregnant**												
Null model	0.06	**0.02**	[0.00;0.00]		0.19	**0.05**	[0.02;0.12]		0.27	**0.08**	[0.05;0.11]	
Model 1**	0.00	**0.00**	[0.00;0.00]	<0.001	0.15	**0.04**	[0.01;0.13]	<0.001	0.17	**0.05**	[0.03;0.08]	<0.001
Model 2	0.00	**0.00**	[0.00;0.00]	0.29	0.11	**0.03**	[0.01;0.14]	0.41	0.17	**0.05**	[0.03;0.08]	0.08

Notes: Model 1 includes respondent variables (age, education, marital status, ever given birth, number of birth events, wealth, previous PMA experience) and community variables (region or state, rural / urban status, presence of facility providing abortion, monthly average number of abortions reported by facilities in community).
Model 2 includes respondent, community, and interviewer / interview-related variables (interviewer age, marital status, children, education, previous PMA staff, previous survey experience, comfort asking questions about abortion, respondent-interviewer familiarity and survey language).
*Excluding parity variables. **Excludes respondent marital status for Rajasthan. IIC = intra-interviewer correlation. CI = confidence intervals. LR test = Likelihood ratio test.
