# Supplementary material for: Interviewer effects on abortion reporting: a multilevel analysis of household survey responses in Côte d’Ivoire, Nigeria and Rajasthan, India
Source: BMJ Open. 2021 Nov 18;11(11):e047570. doi: 10.1136/bmjopen-2020-047570 (PMC8606767; doi:10.1136/bmjopen-2020-047570)
Supplement: Author's manuscript [file bmjopen-2020-047570.draft_revisions.pdf]

BMJ Open

BMJ Open is committed to open peer review. As part of this commitment we make the peer review history of every article we publish publicly available.

When an article is published we post the peer reviewers' comments and the authors' responses online. We also post the versions of the paper that were used during peer review. These are the versions that the peer review comments apply to.

The versions of the paper that follow are the versions that were submitted during the peer review process. They are not the versions of record or the final published versions. They should not be cited or distributed as the published version of this manuscript.

BMJ Open is an open access journal and the full, final, typeset and author-corrected version of record of the manuscript is available on our site with no access controls, subscription charges or pay-per-view fees (http://bmjopen.bmj.com).

If you have any questions on BMJ Open's open peer review process please email info.bmjopen@bmj.com

Interviewer effects on abortion: assessment of survey responses in three countries

Journal:	*BMJ Open*
Manuscript ID	bmjopen-2020-047570
Article Type:	Original research
Date Submitted by the Author:	04-Dec-2020
Complete List of Authors:	Footman, Katharine; The London School of Economics and Political Science, Social Policy
Keywords:	Demography < TROPICAL MEDICINE, STATISTICS & RESEARCH METHODS, REPRODUCTIVE MEDICINE, PUBLIC HEALTH

SCHOLARONE™
Manuscripts

I, the Submitting Author has the right to grant and does grant on behalf of all authors of the Work (as defined in the below author licence), an exclusive licence and/or a non-exclusive licence for contributions from authors who are: i) UK Crown employees; ii) where BMJ has agreed a CC-BY licence shall apply, and/or iii) in accordance with the terms applicable for US Federal Government officers or employees acting as part of their official duties; on a worldwide, perpetual, irrevocable, royalty-free basis to BMJ Publishing Group Ltd ("BMJ") its licensees and where the relevant Journal is co-owned by BMJ to the co-owners of the Journal, to publish the Work in this journal and any other BMJ products and to exploit all rights, as set out in our licence.

The Submitting Author accepts and understands that any supply made under these terms is made by BMJ to the Submitting Author unless you are acting as an employee on behalf of your employer or a postgraduate student of an affiliated institution which is paying any applicable article publishing charge ("APC") for Open Access articles. Where the Submitting Author wishes to make the Work available on an Open Access basis (and intends to pay the relevant APC), the terms of reuse of such Open Access shall be governed by a Creative Commons licence – details of these licences and which Creative Commons licence will apply to this Work are set out in our licence referred to above.

Other than as permitted in any relevant BMJ Author's Self Archiving Policies, I confirm this Work has not been accepted for publication elsewhere, is not being considered for publication elsewhere and does not duplicate material already published. I confirm all authors consent to publication of this Work and authorise the granting of this licence.

For peer review only

Title: Interviewer effects on abortion: assessment of survey responses in three countries

Author: Katharine Footman, Department of Social Policy, London School of Economics and Political Science, Houghton Street, London, WC2A 2AE. K.footman@lse.ac.uk

Abstract:

Objectives: The analysis aimed to assess the scale of interviewer effects on abortion survey responses, to compare interviewer effects between different question wordings and between direct and indirect approaches, and to identify interviewer and interview characteristics that explain interviewer effects on abortion reporting.

Setting: 2018 Performance Monitoring for Action (PMA) nationally representative household surveys from Côte d'Ivoire, Nigeria and Rajasthan, India

Participants: Survey data from 20,016 interviews with reproductive age (15-49) women, selected using multi-stage stratified cluster sampling. Data from self-administered interviewer surveys and from a sample of health service delivery points that serve the female survey participants were also included.

Primary outcome measures: Outcomes were the respondent's own experience of ever 'removing a pregnancy', their closest confidante's experience of pregnancy removal and the respondent's own experience of period regulation.

Results: Substantial interviewer effects were observed, ranging from 7% in Côte d'Ivoire to 24% in Nigeria for pregnancy removal. Interviewer effects for survey questions that were designed to ask about abortion in a less stigmatising way were either similar to (9-26% for confidante-reporting) or higher than (17-32% for a question about period regulation) the pregnancy removal question. Factors associated with abortion reporting included respondent-interviewer familiarity, the language of interview, and the interviewer's comfort asking questions about abortion.

Conclusion: This study highlights that questions designed to be less stigmatising may increase interviewer effects due to lower comprehension among respondents. Further work is needed to assess question wordings for different contexts. Selecting and training interviewers to ensure comfort asking questions about abortion is important for reproductive health surveys. Challenges for the use of 'insider' interviewers and the management of surveys in countries with high linguistic diversity are also identified.

For peer review only

Strengths and limitations of the study:

- This is one of the first studies of interviewer effects on abortion reporting in low- and middle-income countries and it is the first to assess the recently developed Performance Monitoring for Action (PMA) survey abortion module.
- The analysis extends existing knowledge about interviewer effects for abortion as the PMA module included new survey questions, and meta-data allowed interview and interviewer characteristics to be assessed, including respondent-interviewer familiarity.
- It was not possible to entirely separate interviewer effects from area affects, so interviewer effects may be over-estimated, but fixed respondent and community characteristics were included in the analysis to account for area effects to some extent.
- It is not possible to tell whether characteristics associated with higher reporting reflect an association with prior abortion experience or with willingness to report an abortion.

.

For peer review only

1. Introduction

Only 37% of women of reproductive age live in countries where their right to access abortion does not depend on their reason for ending the pregnancy.[1] Almost half of the 73 million abortions that occur each year are unsafe due to these restrictions on access to safe abortion.[2] Despite its importance as a human rights and public health issue, there is limited evidence about abortion in most countries.[3] Abortion data are needed to inform policy and advocacy: evidence about the incidence and impacts of unsafe abortion has played an important role in the liberalisation of abortion laws.[4–6] Strategies to improve equitable access to safe abortion also require high quality data about where and how women are seeking abortion, and which population sub-groups are most affected by barriers to safe abortion care.[7,8]

Unsafe abortion disproportionately (86% of all abortions[9] and 97% of unsafe abortions[1]) affects women in low- and middle-income countries (LMICs), largely driven by laws and policies that restrict access to safe abortion. Data about abortion is often particularly limited in these contexts because health records exclude illegal, informal and self-managed abortions.[10] Interviewer-administered household surveys tend to be key sources of demographic, reproductive and public health data in LMICs.[11–13] However, concerns about under-reporting mean that data about abortion are rarely used and poorly trusted[3,14]: studies from high-income countries that compare survey self-reports to health records suggest that 35-80% of respondents accurately report abortions in surveys.[15–18]

Efforts to improve the availability of abortion data have focussed on estimating abortion incidence using indirect methods[3] or reducing under-reporting in surveys. Some surveys have (unsuccessfully) attempted to reduce under-reporting in direct questions by grouping abortions and miscarriages together [3], adding a filter question about previous unwanted pregnancies[19], or asking for a full pregnancy history.[20,21] A recent review[3] recommended additionally asking women directly if they have used medications to bring on their periods, due to recent changes in the way abortion methods are used and conceptualised. Performance Monitoring for Action (PMA) surveys now include this approach by asking participants about experiences of 'period regulation'.[22] Methods to reduce under-reporting in surveys through indirect questions have produced mixed results[3,17,23] and have a number of flaws that limit the utility of the data they produce. For example, random response methods[24–26] and list experiments[27–32] do not produce individual-level data, have limited precision, and do not permit follow-on questions about sources of abortion and their safety, the process of abortion-seeking or issues with access to abortion care. Confidante reporting[33–35], where the respondent is asked about abortions in their social networks, rather than their own abortions, has also been used in PMA surveys.[10] Confidante reporting can reduce the role of stigma in abortion reporting, though it relies on respondents being aware of their friends' abortions and being willing to report them.[3] Finally, survey researchers have also attempted to reduce under-reporting by limiting the impact of the interviewer through self-administered surveys[36,37], phone interviews[38,39], or audio computer-assisted self-interviewing (ACASI).[23,25] However, these methods can be less feasible in contexts where there is lower literacy, technology use and phone ownership.[3,40] Interviewers therefore continue to play a pivotal role in the quality of survey data, particularly in low-resource settings: they make contact with respondents, explain the purpose of the survey, gain consent, ask questions, record answers, and motivate respondents throughout the interview.[41]

For peer review only

Abortion questions have often been excluded from household surveys for these methodological (and some political) reasons[7], but there are increasing efforts to address this gap. For example, several countries now include direct questions about abortion in Demographic and Health Surveys (DHS)[42,43], and an abortion module was recently included in three countries' PMA (Performance Monitoring for Action) surveys.[44] These surveys offer an opportunity to expand the availability of abortion data. However, more work is needed to explore data quality issues beyond under-reporting, to assess which questions and methods can improve the quality of abortion data from household surveys, and to identify which interviewer characteristics affect abortion reporting.

Interviewers can impact the accuracy of survey responses due to their observed characteristics, such as age or gender, or role-related characteristics, such as the way they read questions, probe or provide instructions.[45] Interviewer effects vary by survey question, but are more likely to cause measurement error for questions that are complex, sensitive, non-factual, open-ended or evoke emotional responses.[46–48] The interviewer effect on the variance of the mean for a survey item can be expressed as $1+\rho_{int}(m-1)$, where m is the average number of interviews completed per interviewer and the interviewer effect ρ_{int} is the intra-class correlation, or intra-interviewer correlation (IIC) of the survey responses.[49] Interviewer effects can range from 1-12% of the variance in survey responses in face-to-face surveys, with most being below 2% [50,51], but even small interviewer effects can have a significant impact on survey data quality, especially if each interviewer collects a large number of responses.[52]

There is a large body of research on interviewer effects in high-income countries[53] but few studies in LMICs[40], despite the increased importance of this topic in these contexts. Household surveys in LMICs often depend more on interviewers for data collection, but interviewers may face additional challenges: notions of households are often complex, linguistic diversity is often higher, familiarity with surveys and acceptability of strangers may be lower, and the power differentials between interviewer and respondent can be wider.[11,54] There have also been very few studies on interviewer effects for reproductive health surveys and this evidence has been mixed. A review of the DHS in Indonesia and the Philippines found low (1%) interviewer effects for questions about contraceptive use[55] while analysis of the DHS in Kenya and Malawi found higher interviewer effects for contraceptive use (ranging between 3-25%), with interviewer gender, marital and fertility status being important covariates of response patterns.[56] The remaining limited literature on interviewer effects in reproductive health surveys in LMICs has focused on the gender of interviewer[57–60] and respondent-interviewer familiarity.[12,13,54,61–63] Only one study has assessed interviewer effects on survey questions about abortion in LMICs, using a cross-classified multilevel model to separate interviewer effects and area effects in 22 DHS surveys. The study found large interviewer effects ranging from 0.2% to 50%, that interviewer effects were stronger for questions about 'abortion' than 'any termination', and that interviewer effects were greater than area effects.[40] However, the interviewer characteristics that might explain these effects could not be assessed in this DHS analysis, due to a lack of meta-data about interviewer and interview characteristics in most DHS surveys.

Building on these findings[40], the present analysis is the first to assess interviewer effects for abortion responses in PMA survey data. PMA surveys used an extended abortion module to collect nationally- and state-representative data about abortion incidence and safety in 2018

For peer review only - http://bmjopen.bmj.com/site/about/guidelines.xhtml

in Nigeria, Cote d'Ivoire, and the state of Rajasthan in India, and expanded on previous survey methodologies for abortion by including new questions designed to increase reporting.[10,22,44] This analysis therefore extends the limited literature on interviewer effects for abortion by assessing and comparing these new survey questions. In addition, PMA surveys include meta-data about interviewer and interview characteristics, so this analysis is the first to assess the characteristics that may explain interviewer effects on abortion questions. PMA surveys also include abortion service delivery data from the area, allowing abortion reporting to be separated from abortion incidence to some extent in this analysis. Finally, the PMA model of using local interviewers enables further exploration of the role of respondent-interviewer familiarity in the context of interviewer effects for abortion.

1.1. Aim

This analysis was intended to assess the scale of interviewer effects on abortion survey responses using PMA data. The aims of the analysis were to compare interviewer effects for abortion reporting: between questions that employ different language to refer to abortion; between direct and indirect approaches to abortion measurement; and with questions about less stigmatised topics. Finally, the analysis aimed to identify interviewer and interview characteristics that explain interviewer effects on abortion reporting.

2. Methods

2.1. Contexts

This analysis used nationally representative PMA survey data collected in Nigeria, Côte d'Ivoire and Rajasthan state, India in 2018, as these surveys included the extended abortion module. The three countries have varying abortion laws, with abortion being broadly legal in India since 1971, while abortion is allowed only to save a woman's life in Côte d'Ivoire and in Nigeria at the federal level. Abortion stigma is prevalent in each of the three contexts, though it can be more evident in countries with more restrictive abortion laws.[64] According to PMA 2018 data, annual abortion incidence rates are substantially lower in Rajasthan, India (23.2 per 1,000 women of reproductive age)[65] than in Côte d'Ivoire (40.7 per 1,000)[66] and Nigeria (45.8 per 1,000).[67] The proportion of abortions that are most unsafe (involving non-recommended methods and non-clinical providers) was estimated at 62% in Côte d'Ivoire, 63% in Nigeria and 31% in Rajasthan.[44] The most recent indirect estimates suggest the majority (73%) of abortions in India are safe medication abortions occurring outside health facilities, while only 5% of abortions involve informal unsafe methods.[68] In Nigeria, use of medication abortions from pharmacies is also increasingly common[69,70], but in both the West African countries, unsafe methods are still most commonly used to end a pregnancy.[71–73]

2.2. Data

PMA is a multi-country project that conducts frequent reproductive health surveys in nine countries in sub-Saharan Africa and Asia. PMA survey methodology is described in detail elsewhere.[74] In brief, the surveys sample reproductive age women, households and health service delivery points using multi-stage stratified cluster sampling. Primary sampling units (PSU) are selected using probability proportional to size procedures, with stratification by urban-rural status (in Côte d'Ivoire and Rajasthan) or state (Nigeria). In each PSU, households are mapped and listed by interviewers and 35 (40 in Lagos state, Nigeria)

households are randomly selected to be invited for a face-to-face interview. Interviewers conduct the informed consent process, and response rates were 98% in each country.

A household interview is conducted, and all females age 15-49 in each household are interviewed using a structured questionnaire that includes demographic characteristics; pregnancy and fertility preferences; contraception; sexual activity; and menstrual hygiene. In 2018, researchers added an abortion module in Round 2 of Cote d'Ivoire, Round 5 of Nigeria, and Round 4 of Rajasthan, India. A survey is also conducted at a sample of health service delivery points (SDPs) that serve the representative sample of reproductive age women. The SDP sample includes the public facilities at each level of the healthcare system that serve each PSU, and up to three private SDPs located in each PSU. The SDP questionnaire collects data on the availability and volumes of a range of health services, including abortion and post-abortion care for the round of data collection under analysis. Additionally, before data collection begins, interviewers self-administer a staff survey with questions about their demographic characteristics and previous experience. This staff survey was not completed in Nigeria in 2018.

PMA surveys are repeated every 6-12 months with a new sample drawn at each round, so frequent, rapid data collection is facilitated using mobile data collection and 'resident enumerators' who live in the enumeration area. Interviewers may therefore be known or familiar to respondents, particularly as they are retained between data collection rounds whenever possible.[13] Interviewers are typically women, over the age of 21, holding at least secondary school education, familiar with mobile phones, and with no affiliation to the local health system.[13]

2.3. Measures

The abortion module used four questions to gather data on experiences of abortion. First, the confidante method was used, where women are asked to report the abortions of a set number of close friends, defined by PMA as "women whom you share secrets with and who also share theirs with you". In this survey, respondents were asked about their two closest friends but only data for the participant's closest confidante were used in this analysis. Participants were asked whether each of their confidantes had ever done something to 1) "remove a pregnancy" and/or to 2) "regulate a period" when she was pregnant or worried she was pregnant. Participants could answer 'yes I am certain', 'yes I think so', 'no', 'don't know' or 'no response', and the variable was dichotomised by grouping positive responses and treating 'no response' as missing (<1% of responses). Respondents were then asked whether they themselves had done something to 1) "remove a pregnancy" and/or 2) "regulate a period" when they were pregnant or worried that they were pregnant. Respondents could answer 'yes', 'no' or 'no response' and non-responses were treated as missing (<1% of responses). For each question, the interviewer was instructed to "*probe to confirm whether the pregnancy removal (or period regulation) was successful"* and unsuccessful attempts were not recorded.

In this analysis, the respondent's own experience of ever 'removing a pregnancy' was considered the main measure of a previous abortion. Interviewer effects for this outcome were compared to the abortion questions designed to be less stigmatising as they avoid personal disclosure[10] or allow for different understandings of abortion[3,75,76]: their closest

confidante's experience of pregnancy removal and the respondent's own experience of period regulation. The interviewer effects for these abortion questions were then compared to topics considered to be less stigmatised: first, whether the respondent or their partner were 'currently doing something or using any method to delay or avoid getting pregnant' (their current contraceptive use); second, whether the respondent reported they had ever given birth, and; third, whether the respondent reported they were currently pregnant.

Explanatory variables were selected based on previous literature about factors associated with abortion reporting[15,18,37,38,77], contraceptive use reporting[61–63], and abortion incidence[9,15,18]. Explanatory variables included respondent, community, interview and interviewer characteristics (see Appendix 2 for further detail). At the respondent-level, explanatory variables included respondent age, education status, marital status, parity (ever given birth and number of births), household wealth, and previous PMA participation. At the community-level, explanatory variables were derived from the individual, household and service delivery data, including urban/rural status, region or state, whether the enumeration area had a SDP that provided abortion or post-abortion care, and the monthly volume of abortion services reported by these SDPs. Interview characteristics included respondent-interviewer familiarity (as recorded by the interviewer for each respondent) and survey language. Interviewer characteristics included age, marital status, education status, whether they had children, previous involvement in PMA data collection, previous survey experience outside of PMA, number of respondents interviewed and self-reported comfort asking questions about abortion.

2.4. Analysis

Descriptive analysis of the outcomes of interest and the potential explanatory variables was conducted to assess their distributions. Bivariate logistic regression analysis was used to assess associations between potential explanatory variables and the odds of reporting removing a pregnancy, period regulation and a confidante's pregnancy removal.

Multi-level logistic regression models with an interviewer random intercept were then sequentially developed to assess the variance in outcomes within- and between-interviewers. Multi-level modelling was used to account for unmeasured interviewer characteristics and non-random allocation of respondents to interviewers, and to address the correlated error terms resulting from multiple respondents being interviewed by the same person and living within the same community (as there is one interviewer per PSU in PMA surveys).[52] Explanatory variables were added to the model sequentially: Model 0 included only the interviewer random effects term, Model 1 also included respondent and community characteristics, and Model 2 additionally included interview and interviewer characteristics.

Model 0 was estimated according to the equation: $\log(\pi_{ij} /1-\pi_{ij}) = \beta_0 + u_j$ where π is the probability of reporting having ever removed a pregnancy; β_0 is the log-odds of reporting having removed a pregnancy when u=0 (i.e. for the average interviewer); and the addition of u_j gives the intercept for interviewer j, or the interviewer effect. Models 1 and 2 were estimated using the equation: $\log(\pi_{ij} /1-\pi_{ij})= \beta_0 + \beta_1 x_{1ij} + \beta_2 x_{2ij} + _{...} + \beta_k x_{kij} + u_j$ where β_0 is the log-odds of reporting having removed a pregnancy when x=0 and u=0; $\beta_1 x_{ij}$ is the effect of a 1-unit change in x on the log-odds, holding constant all other explanatory variables and the interviewer effect u; and u_j is the interviewer effect for interviewer j. The models assume that

(u_j~$N(0,\sigma^2_u)$), meaning the level 2 residuals u_j are assumed to be independent and to follow normal distributions with means of zero. The variance of u_j (σ^2_u) is the level 2 residual variance and was used to calculate the intra-interviewer correlation (IIC) using the equation: IIC = $\sigma_u^2 / \sigma_u^2 + \sigma_e^2$ where σ_e^2 (the level 1 residual variance) is equal to 3.29 for a logit model.[78] The IIC is therefore the proportion of total variance that can be explained by interviewer effects, and the terms 'IIC' and 'interviewer effect' are used interchangeably in the Results.

Analyses were conducted for each country individually. Likelihood ratio tests were used at each stage to assess whether the multi-level model offered a significant improvement on a simple logistic model, and to assess whether the addition of variables improved the fit of the multi-level model. Simple fixed effects logistic regression models were also run at each stage and coefficients were compared between the simple and multi-level models to check for any substantial differences. In descriptive and bivariate analyses, survey weights were used and the complex sampling design was accounted for using the Taylor linearization method. In the multi-level models, clustering and stratification were adjusted for through the inclusion of the interviewer random intercept and inclusion of strata (urban/rural and state) as covariates. The multilevel analysis was not weighted for simplicity, but the weights variable was included as a coefficient in Model 2 as a sensitivity analysis. All other coefficients remained similar to the original model and the weight variable was non-significant in each country. The estimation procedure was maximum likelihood estimation using adaptive quadrature with seven integration points. To assess that seven integration points was adequate, the model was refitted with a larger number of integration points (up to 40) to assess that model parameters were substantially similar[78], and this sensitivity analysis confirmed that the coefficients were stable. Complete records analysis was conducted.

All analyses were conducted in Stata 15.1. Statistical significance was determined using an alpha of 0.05, but coefficients significant at the 0.10 level were also noted.

2.5. Ethics

As this secondary analysis used de-identified and administrative data, the analysis was exempted from review by the London School of Economics and Political Science and the Johns Hopkins Bloomberg School of Public Health ethics review boards.

2.6. Patient and public involvement

No patients were involved in the development of the research questions, analysis or dissemination.

3. Results

The respondent, community, interview and interviewer characteristics are shown in Table 1. Interviewer survey data were only available in Côte d'Ivoire and Rajasthan and in both surveys, all interviewers were female. Interviewers were older in Côte d'Ivoire and a lower proportion were married (20%) than in Rajasthan (80%). More interviewers in Côte d'Ivoire had technical or graduate education (86% vs 51%) and had previous survey experience

For peer review only - http://bmjopen.bmj.com/site/about/guidelines.xhtml

(90% vs 19%). Almost all interviewers reported that they felt comfortable asking questions about abortion in both countries.

The prevalence of reporting a previous abortion for the respondent themselves or their closest confidante is presented in Table 2. Self-reports of having ever removed a pregnancy were highest in Côte d'Ivoire (19%) and Nigeria (15%) compared to Rajasthan (7%). Confidante-reporting was higher than self-report in Rajasthan and Nigeria, but not in Côte d'Ivoire. Reports of ever having regulated a period were significantly lower than reports of ever having removed a pregnancy in each setting, both for self-reporting and for confidante-reporting.

Figures 1 and 2 illustrate the interviewer effects in each country. The caterpillar plots in Figure 1 show the mean log-odds for reporting ever removing a pregnancy to each interviewer, ordered by size, after adjusting for respondent and community characteristics (Model 1). In Figure 2, the interviewer effects for each of the questions is shown for each country (Model 1). Interviewer effects for the pregnancy removal question were substantial in each country but were largest in Nigeria and Rajasthan. Interviewer effect accounted for 7% of the variance in the odds of reporting removing a pregnancy in Côte d'Ivoire, 18% in Rajasthan and 24% in Nigeria. The interviewer effect was higher for the question about period regulation than pregnancy removal in the West African countries, particularly in Côte d'Ivoire (Figure 2). The question about the closest confidante's pregnancy removal had similar interviewer effects to the question about the respondent's own pregnancy removal in each country. Interviewer effects were generally lower for the questions about previous births (ranging from 3-7%) and current pregnancy (0-5%), compared to the questions about abortion. However, interviewer effects for the question about current contraceptive use were similar in size (8-22%) to the questions about abortion.

Table 3 presents Model 2 for the odds of reporting removing a pregnancy in each country, adjusting for respondent, community, interviewer and interview characteristics. Interviewer effects were still substantial in Model 2 for Rajasthan and Nigeria, suggesting that there are other factors not included in the model that may explain the variance between interviewers in these countries (particularly for Nigeria, where it was not possible to include interviewer characteristics in the model). The IIC for each of the models is presented in Appendix 1. Adjusting for interview and interviewer characteristics in Model 2 did reduce the size of the interviewer effects for most countries and questions but did not significantly improve the fit of the model compared to Model 1, particularly for Rajasthan.

In each country (Table 3), respondent characteristics were significantly associated with the odds of reporting a pregnancy removal, in line with previous studies about sub-group differences in abortion reporting[15,18] and abortion incidence.[9,15,18] Broadly, respondents who were older, with more formal education and in a higher wealth quintile were more likely to report a pregnancy removal, though there were some differences between countries. Abortion service availability and volumes were only significant in Nigeria, where the availability of a clinic providing abortion was associated with significantly higher odds of reporting a pregnancy removal, though surprisingly the association with the facility-reported monthly number of abortions was negative.

In both Rajasthan and Nigeria, respondents who were not well acquainted with the interviewer had significantly lower odds of reporting a pregnancy removal compared to

For peer review only

respondents who were well or very well acquainted. However, respondents who were not at all acquainted did not have significantly different odds from those who were well acquainted. There was no significant association with respondent-interviewer familiarity in Côte d'Ivoire, where almost all respondents were not at all familiar with the interviewer. Survey language was significantly associated with the odds of reporting a pregnancy removal in the West African countries, but not in Rajasthan where almost all interviews were conducted in Hindi. In Rajasthan, none of the interviewer characteristics were significant. In Côte d'Ivoire the interviewer's parity, education level and comfort asking questions about abortion were significant at the 0.1 level.

4. Discussion

Interviewer-administered household surveys will continue to be an important source of health information in LMICs for the foreseeable future due to limitations of formal health records[10] and challenges with alternative modes of survey data collection in low-resource settings.[11] A greater understanding of interviewer effects for abortion questions in household surveys can support efforts to improve the quality of these data and increase the availability of much-needed evidence about abortion. This analysis identified substantial interviewer effects for abortion reporting in three PMA survey countries, ranging from 7% in Côte d'Ivoire to 24% in Nigeria for ever having removed a pregnancy. Questions designed to ask about abortion in a less stigmatising way did not have lower interviewer effects, and in fact interviewer effects were higher for a question about period regulation than pregnancy removal in Côte d'Ivoire and Nigeria. Although interviewer effects were considerably smaller for less stigmatised topics such as previous births and current pregnancies, interviewer effects for contraceptive use were similar in scale to questions about abortion. The interviewer characteristics that affect abortion reporting could not be fully explained by the available data, but significant interview and interviewer characteristics identified in this analysis were: respondent-interviewer familiarity, the language of interview, and the interviewer's comfort asking questions about abortion.

Interviewer effects observed in this study were higher than average estimates from surveys in high-income countries, which tend to be below 2% and vary from 1-12%.[50,51] Higher variance between interviewers does not necessarily indicate lower validity of abortion reporting, as each interviewer could discourage true reporting equally, but evidence that less sensitive questions have lower interviewer variance suggests that lower variance may be a sign of higher validity.[40] Interviewer effects can also affect survey data quality by increasing the variance of estimates[52], so understanding, minimising and accounting for interviewer effects is important.

Interviewer effects for questions about contraceptive use in PMA were as high as the abortion questions. The evidence on interviewer effects for contraceptive use questions in DHS data is limited and mixed[55,56] but inaccuracies in self-reports of contraceptive use have been noted in several studies.[79–81] Contraceptive use can be highly sensitive and is often covert.[82] The topic of survey data quality for contraceptive use has been neglected[79], but the high interviewer effects for contraceptive use observed in this analysis suggest that this issue warrants further attention. Unlike abortion, contraceptive use is a widely collected survey item, and its measurement was the original aim of investment in PMA.[74] Though abortion faces the additional issue of under-reporting in surveys, this finding does raise

For peer review only

questions about the frequent exclusion of abortion from reproductive health surveys on methodological grounds, since some of the same quality issues exist for other common reproductive health topics.

To improve the quality of survey data on abortion, further assessment of the most effective survey questions is required. The questions used in the PMA surveys were the result of extensive pilot testing that aimed to appropriately capture the nuance of how women discuss and refer to abortion experiences.[67,83] Although the PMA pilot in Côte d'Ivoire suggested high understanding of the period regulation question[66], the higher interviewer effects for period regulation in both West African countries may have been caused by respondent confusion about the meaning of the question, resulting in the need for additional, unscripted clarification or explanation by the interviewers. Interviewer effects were similar for self-reporting and confidante-reporting, suggesting that this data quality issue is not reduced by using indirect reporting. Further cognitive interviewing and formative research could support identification of the most effective wording for asking about previous abortion experiences, and further work could also consider how 'removing a pregnancy' is understood compared to other commonly used terminologies. Use of familiar words is important for describing sensitive behaviours[84], but these are likely to vary considerably by context, creating challenges for cross-country comparisons. The finding that abortion reporting varied significantly based on the language of interview poses additional challenges for cross-national surveys, but also for countries where there are high levels of linguistic diversity.

The findings indicate that socio-demographic characteristics of interviewers were not significantly associated with respondents reporting a pregnancy removal, suggesting that the interview context or interviewer skills and behaviours may be more important than their observable characteristics. Previous studies of interviewer effects have often found the predictive power of variables from interviewer surveys are low, explaining only a small proportion of observed variance, but interviewer surveys can be strengthened through inclusion of questions relating to attitudes, behaviours, experiences and expectations of survey outcomes.[41] Use of interviewer observation or para-data on, for example, the speed of interview, may also provide further insight into the interviewer skills and behaviours that improve the validity of abortion reporting. Comfort asking questions about abortion was reportedly high but was significantly associated with abortion reporting at the 0.1 significance level in Côte d'Ivoire, suggesting additional values clarification training on abortion for interviewers may improve the quality of their data.

In Rajasthan and Nigeria, respondent-interviewer familiarity was significantly associated with abortion reporting. Being slightly acquainted with an interviewer was negatively associated with abortion reporting compared to being well-acquainted, but there was no significant difference between reporting to a close acquaintance or a complete stranger. The historical norm of surveys has been to have strangers as interviewers, as respondents may wish to avoid judgement from a peer, or fear that their answers will not remain confidential. However, use of 'insiders' as interviewers may increase the interviewers' understanding of local culture, increase the likelihood of being invited into a private space, and can promote rapport, trust and closeness which can increase the motivation to answer truthfully.[54,61,85] The impact of interviewer-respondent familiarity will likely vary over time, and with the level of familiarity.[61] In these PMA surveys, it seems that a loose tie between the interviewer and respondent may not be conducive to abortion reporting, which may relate to concerns about

For peer review only

confidentiality and trust, compared to a stranger or a close acquaintance. This finding may support future use of stranger interviewers, as it is difficult to systematically ensure that acquainted interviewers are closely acquainted, rather than slightly acquainted, with all respondents. However the results contrast with previous research that found limited difference in contraceptive reporting to local-insider and local-stranger interviewers in the Dominican Republic, while over-reporting was significantly higher to outsider-interviewers[62], suggesting that the benefits of personal and local familiarity may vary by context and topic.

4.1. Strengths and limitations

This analysis has a number of limitations. First, the most significant limitation of this study was that it was not possible to entirely separate interviewer effects and area effects, as the survey does not have an interpenetrated design where respondents or geographic areas are randomly assigned to interviewers, and cross-classified modelling could not be used to separate area and interviewer effects because one interviewer is assigned to each cluster in PMA surveys. This is problematic as respondents in different clusters may have different probabilities of reporting abortions due to geographic, cultural or demographic differences so interviewer effects may be over-estimated in this study since our findings combine interviewer and area effects. However, studies have found area random effects to be non-significant after controlling for household fixed effects in cross-classified models[86], and the previous DHS analysis, which could separate interviewer effects and area effects, also found substantial interviewer effects for abortion reporting, which were greater than area effects.[40] Second, it is not possible to tell whether variables associated with higher abortion reports reflect an association with prior abortion experience or a willingness to report it. Though the inclusion of facility-reported data on number of abortions and abortion service availability was intended to partially account for this issue, there are likely inaccuracies in the facility-reports of abortion caseload. Third, interviewer survey data was missing for 4% of respondents in Côte d'Ivoire and 6% of respondents in Rajasthan. However, sensitivity analysis was conducted to compare the coefficients and standard errors for Model 1 when restricting the model to those cases that had complete interviewer survey data, and the results did not vary when comparing the model using all data to the model using only non-missing data. Fourth, for the sake of simplicity, the models do not include interactions between respondent and interviewer characteristics, which could be explored in future research.

This study also has several strengths. It is the first to assess interviewer effects in the expanded abortion module used by the PMA surveys in 2018, and it is one of the first to assess interviewer effects for abortion questions in household surveys in LMICs. The analysis used data collected from diverse settings in the same time period using standardised questionnaires and trainings. The data provide a rich range of respondent and community characteristics, including abortion service delivery environment data, as well as interviewer characteristics (in two countries). Finally, the PMA model of using local interviewers enabled further exploration of the role of respondent-interviewer familiarity in the context of interviewer effects.

5. Conclusion

For peer review only

Surveys offer one of the only opportunities to gather representative evidence about the sources and safety of abortions, the sub-groups most affected by unsafe abortion and their abortion-seeking pathways. These data are critical to inform strategies, policies and programmes, and will become even more important with the shift towards self-managed medication abortions using drugs purchased informally through pharmacies or online. Understanding interviewer effects for abortion reporting can help inform decisions about whether to include abortion questions in demographic and public health surveys, which questions to use, and how abortion survey data quality can be improved through methodological adjustments. This analysis highlights that interviewer effects for abortion reporting were high in the PMA surveys in Côte d'Ivoire, Nigeria and Rajasthan from 2018. Further work is needed to identify the effect of different question wordings on abortion reporting through comparative studies and more comprehensive qualitative and cognitive interviewing. Observable interviewer characteristics were not significantly associated with abortion reporting, suggesting skills and behaviours of interviewers may be responsible for unexplained variance at the interviewer level, and this could be further explored through the addition of relevant variables to the interviewer survey. Additional values clarification trainings or other mechanisms to address issues of abortion stigma and ensure interviewers feel comfortable asking questions about abortion may improve the quality of abortion data from surveys. Respondent-interviewer familiarity and language of interview were also significantly associated with abortion reporting, which raises challenges for survey logistics when using 'insider' interviewers, for countries with high linguistic diversity and cross-national surveys. Consideration of variations in language should inform testing of different question wordings and designs in future work.

Funding: The UK Economic and Social Research Council funded this analysis as part of a doctoral grant (Award/Grant number is not applicable). The PMA abortion module was funded by an Anonymous Donor. The funders had no role in study design, data collection and analysis, decision to publish, or preparation of the manuscript.

Acknowledgements: I am extremely grateful to Dr Suzanne Bell for enabling my access to the PMA interviewer data, for her feedback on the analysis and paper, and for her support throughout the project. This study was completed as part of a Masters dissertation, supervised by Professor Patrick Sturgis, and I am very grateful for his advice and guidance. I would also like to thank my PhD supervisors, Professor Ernestina Coast and Dr Tiziana Leone, for their reviews of this paper, and Dr Laura Sochas for very helpful discussions at the analysis stage. I am grateful to all the researchers involved in the design, management and sharing of PMA survey data at Johns Hopkins Bloomberg School of Public Health, the Centre for Research, Evaluation Resources and Development (CRERD), the Écoles Nationale de Statistiques et d'Economie Appliquee (ENSEA) and the Indian Institute of Health Management Research. Finally, I would like to gratefully acknowledge the time and contributions made by the participants of each of the surveys.

Contributorship statement: This analysis was conceptualised and conducted, and the manuscript was written, by the sole author.

Data sharing statement: PMA survey datasets can be requested through the PMA website: https://www.pmadata.org/data/available-datasets/request-access-datasets.

For peer review only

Bibliography

1. Singh S, Remez L, Sedgh G, Kwok L, Tsuyoshi O. *Abortion Worldwide 2017: Uneven Progress and Unequal Access*. New York; 2018.
2. Ganatra B, Gerdts C, Rossier C, et al. Global, regional, and subregional classification of abortions by safety, 2010–14: estimates from a Bayesian hierarchical model. *Lancet*. 2017;390(10110):2372-2381.
3. Sedgh G, Keogh SC. Novel approaches to estimating abortion incidence. *Reprod Health*. 2019;16(1):1-10.
4. Thapa S. Abortion law in Nepal: The road to reform. *Reprod Health Matters*. 2004;12(24):85-94.
5. Chavkin W, Stifani BM, Bridgman-Packer D, Greenberg JMS, Favier M. Implementing and expanding safe abortion care: An international comparative case study of six countries. *Int J Gynecol Obstet*. 2018;143:3-11.
6. Bell S. Abortion Reporting and Underreporting: Can Better Design Yield Better Data? Doctoral Dissertation, Johns Hopkins University. 2018. http://jhir.library.jhu.edu/handle/1774.2/59185.
7. Harvey P. Medical abortion: the hidden revolution. *J Fam Plann Reprod Heal Care*. 2015;41(3):193-196.
8. Bullough C, Meda N, Makowiecka K, Ronsmans C, Achadi EL, Hussein J. Current strategies for the reduction of maternal mortality. *BJOG An Int J Obstet Gynaecol*. 2005;112(9):1180-1188.
9. Chae S, Desai S, Crowell M, Sedgh G, Singh S. Characteristics of women obtaining induced abortions in selected low- and middle-income countries. *PLoS One*. 2017;12(3):1-19.
10. Bell SO, Shankar M, Omoluabi E, et al. Social network-based measurement of abortion incidence: promising findings from population-based surveys in Nigeria, Cote d'Ivoire, and Rajasthan, India. *Popul Health Metr*. 2020;18(1):1-15. doi:10.1186/s12963-020-00235-y
11. Randall S, Coast E, Compaore N, Antoine P. The power of the interviewer. *Demogr Res*. 2013;28:763-792.
12. Bell SO, Bishai D. The impact of respondent–interviewer familiarity and repeated survey participation on abortion reporting: Evidence from rajasthan, india. *Int Perspect Sex Reprod Health*. 2019;44(4):147-156.
13. Safi S, Greenleaf A, Hawes M, et al. Resident Interviewers and Repeat Surveys: Effects on Measures of Reproductive Health. In: *Population Associations of America Conference*. Chicago; 2017.
14. MacQuarrie KLD, Winfrey W, Meijer-Irons J, Morse AR. Consistency of reporting of terminated pregnancies in DHS calendars . *DHS Methodol Reports No 25* . 2018;(September). http://dhsprogram.com/pubs/pdf/MR25/MR25.pdf.
15. Anderson BA, Katus K, Puur A, Silver BD. The Validity of Survey Responses on Abortion : Evidence from Estonia. *Demography*. 1994;31(1):115-132.
16. Rossier C. Estimating induced abortion rates: A review. *Stud Fam Plann*. 2003;34(2):87-102.
17. Edmeades J, Nyblade L, Malhotra A, MacQuarrie K, Parasuraman S, Walia S. Methodological innovation in studying abortion in developing countries: A "narrative" quantitative survey in Madhya Pradesh, india. *J Mix Methods Res*. 2010;4(3):176-198.
18. Udry JR, Gaughan M, Schwingl PJ, Berg BJ Van Den. A Medical Record Linkage

For peer review only

Analysis of Abortion Underreporting. *Fam Plann Perspect*. 1996;28(5):228-231.

19. Huntington D, Mensch B, Miller VC. Survey Questions for the Measurement of Induced Abortion. *Stud Fam Plann*. 1996;27(3):155.

20. Scott R, Bajos N, Wellings K, Slaymaker E. Comparing reporting of abortions in three nationally-representative surveys: Methodological and contextual influences. *BMJ Sex Reprod Heal*. 2019;45(3):213-219.

21. Moreau C, Bajos N, Bouyer J. Question comprehension and recall: the reporting of induced abortions in quantitative surveys on the general population. *Population (Paris)*. 2004;59(3):439-454.

22. Bell S, Shankar M, Guiella G, et al. P33 Menstrual regulation: Incidence, methods, and sources of this understudied reproductive practice in three countries. *Contraception*. 2020;102(4):287.

23. Lindberg L, Scott RH. Effect of ACASI on Reporting of Abortion and Other Pregnancy Outcomes in the US National Survey of Family Growth. *Stud Fam Plann*. 2018;49(3):259-278.

24. Lara D, García SG, Ellertson C, Camlin C, Suárez J. The measure of induced abortion levels in Mexico using random response technique. *Sociol Methods Res*. 2006;35(2):279-301.

25. Lara D, Strickler J, Olavarrieta CD, Ellertson C. Measuring induced abortion in Mexico: a comparison of four methodologies. *Sociol Methods Res*. 2004;32(4):529-558.

26. Singh S, Remez L, Tartaglione A. *Methodologies for Estimating Abortion Incidence and Abortion- Related Morbidity: A Review*. New York and Paris; 2010.

27. Moseson HS, Gerdts C, Fuentes L, et al. Measuring Texas women's experiences with abortion self-induction using a list experiment. *Contraception*. 2017;96(4):272.

28. Moseson H, Massaquoi M, Dehlendorf C, et al. Reducing under-reporting of stigmatized health events using the List Experiment: Results from a randomized, population-based study of abortion in Liberia. *Int J Epidemiol*. 2015;44(6):1951-1958.

29. Cowan SK, Wu LL, Makela S, England P. Alternative estimates of lifetime prevalence of abortion from indirect survey questioning methods. *Perspect Sex Reprod Health*. 2016;48(4):229-234.

30. Elewonibi B, Gleason S, Msuya S, Shah I, Mahande M, Canning D. Estimating Prevalence of Abortion in Tanzania via Direct Question and the List Experiment. In: *APHA's 2019 Annual Meeting and Expo (Nov. 2-Nov. 6)*. American Public Health Association; 2019.

31. Bell SO, Bishai D. Can a List Experiment Improve Validity of Abortion Measurement? *Stud Fam Plann*. 2019;50(1):43-61.

32. Keogh SC, Otupiri E, Chiu DW, et al. Estimating the incidence of abortion: a comparison of five approaches in Ghana. *BMJ Glob Heal*. 2020;5(4):e002129.

33. Rossier C. Measuring abortion with the Anonymous Third Party Reporting Method. In: Singh S, Remez L, Tartiglione A, eds. *Methodologies for Estimating Abortion Incidence and Abortion-Related Morbidity: A Review.* New York and Paris: Guttmacher Institute and IUSSP; 2010:99-106. http://www.guttmacher.org/pubs/compilations/IUSSP/IUSSP-Chapter7.pdf.

34. Elul B. Anonymous third party reporting of induced abortion: An experiment in Rajasthan, India. In: *Annual Meeting of the Population Association of America*. Boston; 2004.

35. Yeatman S, Trinitapoli J. Best-friend reports: A tool for measuring the prevalence of

For peer review only

sensitive behaviors. *Am J Public Health*. 2011;101(9):1666-1667.

36. Tourangeau R, Yan T. Sensitive Questions in Surveys. *Psychol Bull*. 2007;133(5):859-883.

37. Jones EF, Forrest JD. Underreporting of abortion in surveys of U.S. women: 1976 to 1988. *Demography*. 1992;29(1):113-126.

38. Jagannathan R. Relying on surveys to understand abortion. *Am J Public Health*. 2001;91(11):1825-1831.

39. Broussard K, Hathi P, Coffey D. Assessing public awareness and use of medical abortion via mobile phone survey in India. *Contraception*. 2019;100(6):457-463.

40. Leone T, Sochas L, Coast E. Depends who's asking: interviewer effects in Demographic and Health Surveys abortion data. *Demography*. 2020;In press.

41. Blom AG, Julie M. Korbmacher. Measuring Interviewer Characteristics Pertinent to Social Surveys: A Conceptual Framework. *Surv Methods Insights from F*. 2013:1-16.

42. GSS, GHS, ICF. *Ghana Maternal Health Survey 2017.* Accra, Ghana; 2018.

43. IIPS, ICF. *National Family Health Survey (NFHS-4), 2015-16: India*. Mumbai; 2017.

44. Bell SO, OlaOlorun F, Shankar M, et al. Measurement of abortion safety using community-based surveys: Findings from three countries. *PLoS One*. 2019;14(11):1-14.

45. Davis RE, Couper MP, Janz NK, Caldwell CH, Resnicow K. Interviewer effects in public health surveys. *Health Educ Res*. 2010;25(1):14-26.

46. Schnell R, Kreuter F. Separating interviewer and sampling-point effects. *J Off Stat*. 2005;21(3):389-410.

47. Pickery J, Loosveldt G. An Exploration of Question Characteristics that Mediate Interviewer Effects on Item Nonresponse. *J Off Stat*. 2001;17(3):337-350.

48. Schaeffer NC, Dykema J, Maynard D. Interviewers and Interviewing. In: Marsden P, Wright J, eds. *Handbook of Survey Research*. 2nd ed. Bingley: Emerald Group Publishing Limited; 2014:437-471.

49. West BT, Conrad FG, Kreuter F, Mittereder F. Nonresponse and measurement error variance among interviewers in standardized and conversational interviewing. *J Surv Stat Methodol*. 2018;6(3):335-359.

50. West BT, Olson K. How much of interviewer variance is really nonresponse error variance? *Public Opin Q*. 2010;74(5):1004-1026. doi:10.1093/poq/nfq061

51. Groves RM, Magilavy LJ. Measuring and explaining interviewer effects in centralized telephone surveys. *Public Opin Q*. 1986;50(2):251-266.

52. Hox JJ. Hierarchical Regression Models for Interviewer and Respondent Effects. *Sociol Methods Res*. 1994;22(3):300-318.

53. West BT, Blom AG. Explaining interviewer effects: A research synthesis. *J Surv Stat Methodol*. 2017;5(2):175-211.

54. Weinreb A, Sana M, Stecklov G. Strangers in the Field: A Methodological Experiment on Interviewer–Respondent Familiarity. *BMS Bull Sociol Methodol*. 2018;137-138(1):94-119.

55. Amos M. Interviewer effects on patterns of nonresponse: Evaluating the impact on the reasons for contraceptive nonuse in the Indonesia and the Philippines DHS. *Demogr Res*. 2018;39(1):415-430.

56. Assche SB Van, Reniers G, Weinreb AA. An assessment of the KDICP and MDICP data quality: Interviewer effects, question reliability and sample attrition. *Demogr Res*.

For peer review only

2003;9(1):31-76.

57. Agula J, Barrett JB, Tobi H. The other side of rapport: Data collection mode and interviewer gender effects on sexual health reporting in Ghana. *Afr J Reprod Health*. 2015;19(3):111-117.

58. Becker S, Feyisetan K, Makinwa-Adebusoye P. The Effect of the Sex of Interviewers on the Quality of Data in a Nigerian Family Planning Questionnaire. *Stud Fam Plann*. 1995;26(4):233.

59. Johnson K, Grant M, Khan S, Moore Z, Armstrong A, Sa Z. *Fieldwork-Related Factors and Data Quality in the Demographic and Health Surveys Program.* Calverton; 2009.

60. McCombie SC, Anarfi JK. The influence of sex of interviewer on the results of an AIDS survey in Ghana. *Hum Organ*. 2002;61(1):51-57.

61. Anglewicz P, Akilimali P, Eitmann LP, Hernandez J, Kayembe P. The relationship between interviewer-respondent familiarity and family planning outcomes in the Democratic Republic of Congo: A repeat cross-sectional analysis. *BMJ Open*. 2019;9(1):1-9.

62. Stecklov G, Weinreb AA, Sana M, Anglewicz P. Family planning for strangers: An experiment on the validity of reported contraceptive use. *PLoS One*. 2015;10(8):1-19.

63. Weinreb A. The Limitations of Stranger-Interviewers in Rural Kenya. *Am Sociol Rev*. 2006;71:1014-1039.

64. Shellenberg KM, Moore AM, Bankole A, et al. Social stigma and disclosure about induced abortion: Results from an exploratory study. *Glob Public Health*. 2011;6(1):111-125.

65. Ahmad D. Induced Abortion Incidence and Safety in Rajasthan, India: Evidence that Expansion of Services is Needed. *Stud Fam Plann*. 2020.

66. Bell SO, Sheehy G, Hyacinthe AK, Guiella G, Moreau C. Induced abortion incidence and safety in Côte d'Ivoire. *PLoS One*. 2020;15(5):e0232364.

67. Bell SO, Omoluabi E, OlaOlorun F, Shankar M, Moreau C. Inequities in the incidence and safety of abortion in Nigeria. *BMJ Glob Heal*. 2020;5(1).

68. Singh S, Shekhar C, Acharya R, et al. The incidence of abortion and unintended pregnancy in India, 2015. *Lancet Glob Heal*. 2018;6(1):e111-e120.

69. Akiode A, Fetters T, Okoh M, et al. The availability of misoprostol in pharmacies and patent medicine stores in two Nigerian cities. *Ebonyi Med J*. 2010;9(2).

70. Stillman M, Owolabi O, Fatusi AO, et al. Women's self-reported experiences using misoprostol obtained from drug sellers: a prospective cohort study in Lagos State, Nigeria. *BMJ Open*. 2020;10(5):e034670.

71. PMA2020. *PMA2020 Abortion Survey Results: Cote D'Ivoire*.; 2018. https://www.pma2020.org/sites/default/files/Cote d'Ivoire_AbortionModule_Brief_EN_012019.pdf.

72. PMA2020. *PMA2020 Abortion Survey Results: Nigeria*.; 2018. https://www.pma2020.org/sites/default/files/AbortionModule_Brief_111518.pdf.

73. Guillaume A, Du Loû AD. Fertility regulation among women in Abidjan, Côte d'Ivoire: contraception, abortion or both? *Int Fam Plan Perspect*. 2002;28(3):159-166.

74. Zimmerman L, Olson H, Group PPI, Tsui A, Radloff S. PMA2020: Rapid turn-around survey data to monitor family planning service and practice in ten countries. *Stud Fam Plann*. 2017;48(3):293-303.

75. Plummer ML, Wamoyi J, Nyalali K, et al. Aborting and Suspending Pregnancy in Rural Tanzania: An Ethnography of Young People's Beliefs and Practices. *Stud Fam*

For peer review only

Plann. 2008;39(4):281-292.

76. Ladipo OA, Ojo OA, James S, Stewart KR. Menstrual regulation in Ibadan, Nigeria. *Int J Gynecol Obstet*. 1978;15(5):428-432.

77. Jones RK, Kost K. Underreporting of induced and spontaneous abortion in the United States: An analysis of the 2002 National Survey of Family Growth. *Stud Fam Plann*. 2007;38(3):187-197.

78. Steele F. Multilevel Models for Binary Responses. LEMMA VLE Module 7.

79. Stuart GS, Grimes DA. Social desirability bias in family planning studies: a neglected problem. *Contraception*. 2009;80(2):108-112.

80. Smith C, Edwards P, Free C. Assessing the validity and reliability of self-report data on contraception use in the MObile Technology for Improved Family Planning (MOTIF) randomised controlled trial. *Reprod Health*. 2018;15(1):50.

81. Nwaohiri AN, Tang JH, Stanczyk F, et al. Discordance between self-reported contraceptive use and detection of exogenous hormones among Malawian women enrolling in a randomized clinical trial. *Contraception*. 2018;97(4):354-356.

82. Biddlecom AE, Fapohunda BM. Covert Contraceptive Use: Prevalence, Motivations, and Consequences. *Stud Fam Plann*. 1998;29(4):360-372. doi:10.2307/172249

83. Bell S, Shankar M, Omoluabi E, et al. Methodological Advances in Survey-Based Abortion Estimation: Promising Findings from Nigeria, India, and Cote d'Ivoire. In: *Population Association of America Annual Meeting*. Austin, Texas; 2019.

84. Groves RM, Fowler FJ, Couper MP, Lepkowski JM, Singer E, Tourangeau R. *Survey Methodology*. 2nd ed. Wiley; 2009.

85. Kühne S. From strangers to acquaintances? Interviewer continuity and socially desirable responses in panel surveys. *Surv Res Methods*. 2018;12(2):121-146.

86. Vassallo R, Durrant G, Smith P. Separating interviewer and area effects by using a cross-classified multilevel logistic model: simulation findings and implications for survey designs. *J R Stat Soc*. 2017;180(2):531-550.

For peer review only

Table 1. Characteristics of respondents, communities, interview and interviewer by country (weighted)

	Côte d'Ivoire (n=2798)		Rajasthan, India (n=5915)		Nigeria (n=11,303)	
	N	%	N	%	N	%
**Respondent characteristics**						
**Mean age** (SE)	28.5 (0.21)		29.1 (0.15)		29.1 (0.13)	
**Education**						
No formal schooling	1,282	45%	2,212	37%	2,389	18%
Primary school	733	26%	1,424	24%	1,931	15%
Secondary school	626	23%	1,509	26%	5,039	47%
Higher education	154	6%	770	14%	1,944	20%
**Marital status**						
Currently married	1,187	41%	4,547	77%	7,069	59%
Living with partner	622	24%	9	0%	414	5%
Divorced or widowed	129	4%	153	3%	528	5%
Never married	859	31%	1,188	20%	3,288	31%
**Parity**						
Ever given birth	2,080	74%	4,082	69%	7,456	65%
Mean number of births (SE)	2.5 (0.12)		1.9 (0.04)		2.3 (0.06)	
Previous PMA participant	387	23%	1,281	25%	1,884	23%
**Community characteristics**						
Urban	1,768	62%	1,503	35%	5,664	57%
Rural	1,096	38%	4,561	65%	5,875	43%
Abortion care facility in community	2,129	76%	1,624	27%	5,602	50%
Mean monthly facility-reported number of abortions, per community with abortion facility (SE)	2.7 (0.09)		12.7 (0.51)		5.9 (0.11)	
**Interview characteristics**						
**Interviewer-respondent familiarity**						
Very or well acquainted	42	1%	3,286	53%	1,783	19%
Not well acquainted	109	3%	1,893	33%	3,654	32%
Not at all acquainted	2,654	95%	741	14%	5,915	49%
**Language of Interview**						
French / Hindi / Hausa	1,977	71%	5,673	94%	5,494	48%
Baoule / English / English	170	6%	44	2%	4,350	37%
Yacouba / Other / Igbo	69	3%	198	4%	850	8%
Attie / - / Yoruba	29	1%			159	2%
Dioula / - / Pidgin	438	14%			247	3%
Lobi / - / Other	54	3%			203	3%
Other / - / -	2%	2%				
**Interviewer characteristics**	Côte d'Ivoire (n=70)		Rajasthan, India (n=145)			
**Socio-demographics**						
Female	70	100%	134	100%		
Mean age (SE)		31.1 (0.63)		26.9 (0.55)		
Married	14	20%	107	80%		
Has children	43	61%	86	64%		
**Education**						
Primary or secondary	10	14%	65	49%		
Technical or graduate	60	86%	69	51%		
**Experience**						
Existing staff	68	97%	111	83%		
Previous survey experience	63	90%	26	19%		
Comfortable asking about abortion	66	94%	130	97%		
Existing staff	68	97%	111	83%		

Abbreviations: SE = Standard Error. Note: Interviewer survey data was not collected in Nigeria (n=285). Interviewer survey data were missing for 3 interviewers (123 respondents) in Côte D'Ivoire and 11 interviewers (384 respondents) in Rajasthan.

For peer review only

Table 2. Reported abortions of respondents and their closest confidante by country, weighted

	Côte d'Ivoire			Rajasthan, India			Nigeria		
	N	%	95% CI	N	%	95% CI	N	%	95% CI
**Self-reported**									
Pregnancy removal	511	19	[16;22]	390	7	[6;8]	1,392	15	[13;16]
Period regulation	222	7	[5;10]	109	2	[1;2]	679	7	[6;8]
**Closest confidante**									
Pregnancy removal	305	18	[14;21]	705	15	[12;17]	1,120	20	[18;22]
Period regulation	161	8	[6;22]	294	6	[4;8]	556	9	[8;11]
Total n respondents	2,795			5,912			11,254		
Total n confidantes	1,803			4,983			5,986		

Notes: For self-reported pregnancy removal a small number of respondents were coded as -99 (no response) in each country: 3 (0.1%) in Côte D'Ivoire, 3 (0.04%) in Rajasthan, 49 (0.4%) in Nigeria. For self-reported period regulation, the number of non-responses were 2, 9 and 47 respectively.
A high proportion of respondents reported that they did not have any close female confidantes age 15-49 who they mutually shared very personal information with: 991 (35.5%) in Côte D'Ivoire, 893 (15.2%) in Rajasthan and 4984 (45.4%) in Nigeria, resulting in a smaller sample size for these questions.

For peer review only

Table 3: Full multilevel random intercept logit model (Model 2) for the odds of reporting removing a pregnancy, with respondent, community, interview and interviewer characteristics

	Côte d'Ivoire		Rajasthan, India		Nigeria	
	OR	[95% CI]	OR	[95% CI]	OR	[95% CI]
**Respondent Characteristics**						
Age	**1.35**	**[1.22;1.50]**	**1.40**	**[1.21;1.61]**	**1.46**	**[1.38;1.56]**
Age squared	**1.00**	**[0.99;1.00]**	**0.99**	**[0.99;1.00]**	**0.99**	**[0.99;1.00]**
No formal schooling	Ref		Ref		Ref	
Primary school	**1.96**	**[1.48;2.60]**	**2.01**	**[1.48;2.72]**	**1.62**	**[1.21;2.16]**
Secondary school	**2.10**	**[1.50;2.92]**	**1.46**	**[1.00;2.14]**	**2.19**	**[1.63;2.94]**
Higher education	1.31	[0.8;2.150]	1.49	[0.92;2.41]	**1.94**	**[1.39;2.7]**
Married	Ref		Ref		Ref	
Living with partner	**2.85**	**[2.12;3.83]**	1.33	[0.14;13.0]	**2.75**	**[2.07;3.65]**
Divorced or widowed	**2.25**	**[1.39;3.63]**	0.94	[0.47;1.89]	**1.44**	**[1.11;1.87]**
Never married	**3.66**	**[2.54;5.27]**	**0.14**	**[0.04;0.51]**	**1.30**	**[1.01;1.67]**
Ever given birth (vs never)	**3.63**	**[2.41;5.46]**	**2.76**	**[1.49;5.13]**	**1.39**	**[1.09;1.79]**
Number of birth events	**0.92**	**[0.85;1.00]**	1.08	[0.97;1.19]	***0.96***	***[0.92;1.01]***
Lowest wealth quintile	Ref		Ref		Ref	
Lower quintile	1.36	[0.90;2.05]	0.81	[0.51;1.29]	**1.44**	**[1.10;1.90]**
Middle quintile	1.23	[0.78;1.95]	1.06	[0.67;1.66]	***1.33***	***[0.96;1.84]***
Higher quintile	***1.61***	***[0.97;2.64]***	0.88	[0.54;1.43]	**1.50**	**[1.07;2.11]**
Highest quintile	**1.88**	**[1.12;3.15]**	1.07	[0.64;1.78]	**1.75**	**[1.22;2.52]**
Previous PMA respondent (vs not)	1.26	[0.90;1.75]	0.82	[0.62;1.10]	0.91	[0.74;1.10]
**Community Characteristics**						
Region or state	0.99	[0.97;1.01]	0.99	[0.97;1.01]	1.01	[0.94;1.08]
Rural (vs urban)	0.99	[0.68;1.46]	**0.57**	**[0.36;0.91]**	0.77	[0.56;1.05]
Monthly number of abortions per community (mean, facility-reported)	0.99	[0.95;1.04]	1.00	[0.97;1.02]	**0.97**	**[0.94;0.99]**
Abortion care facility in community (vs none)	0.75	[0.55;1.03]	1.42	[0.90;2.26]	**1.36**	**[1.04;1.78]**
**Interview Characteristics**						
Very well or well acquainted	Ref		Ref		Ref	
Not well acquainted	1.22	[0.28;5.22]	**0.68**	**[0.49;0.96]**	**0.75**	**[0.58;0.97]**
Not acquainted	1.67	[0.45;6.17]	0.91	[0.57;1.45]	0.85	[0.65;1.13]
Language of interview						
French / Hindi / Hausa	Ref		Ref		Ref	
Baoule / English / English	**3.11**	**[2.00;4.83]**	0.77	[0.08;6.97]	**1.59**	**[1.19;2.14]**
Yacouba / Other / Igbo	0.54	[0.15;1.93]	1.54	[0.69;3.47]	1.13	[0.72;1.78]
Attie / - / Yoruba	0.75	[0.28;2.02]			**1.94**	**[1.16;3.25]**
Dioula / - / Pidgin	**0.26**	**[0.14;0.48]**			**1.57**	**[0.99;2.47]**
Lobi / - / Other	***0.17***	***[0.02;1.35]***			1.61	[0.73;3.55]
Other / - / -	0.67	[0.27;1.72]				
**Interviewer characteristics**						
Number of respondents	1.00	[0.98;1.01]	0.99	[0.97;1.01]	**0.99**	**[0.98;1.00]**
Interviewer age	0.99	[0.96;1.02]	1.02	[0.99;1.06]		
Married (vs not married)	1.17	[0.84;1.64]	0.73	[0.39;1.34]		
Has children (vs no children)	***1.33***	***[0.96;1.83]***	1.52	[0.85;2.74]		
Technical / Uni grad (vs secondary)	***1.47***	***[0.95;2.29]***	1.08	[0.73;1.59]		
Existing staff (vs new staff)	1.34	[0.52;3.44]	1.11	[0.66;1.87]		
Previous survey experience (v none)	1.36	[0.82;2.26]	1.01	[0.62;1.64]		
Very comfortable asking about abortion (vs somewhat/not comfortable)	***1.88***	***[0.9;3.9]***	1.55	[0.45;5.34]		
**Intra-Interviewer Correlation**	**0.01**		**0.15**		**0.22**	

For peer review only

Notes: Coefficients in bold are significant at the <0.05 level, coefficients in bold and italics are significant at the <0.10 level. Interviewer characteristics were unavailable for Nigeria. OR = odds ratio. CI = confidence intervals.

For peer review only

For peer review only

For peer review only - http://bmjopen.bmj.com/site/about/guidelines.xhtml

Figure 1. Caterpillar plots showing interviewer effects (Level 2 Residuals) with 95% confidence intervals for the log-odds of reporting ever removing a pregnancy, adjusted for respondent and community characteristics

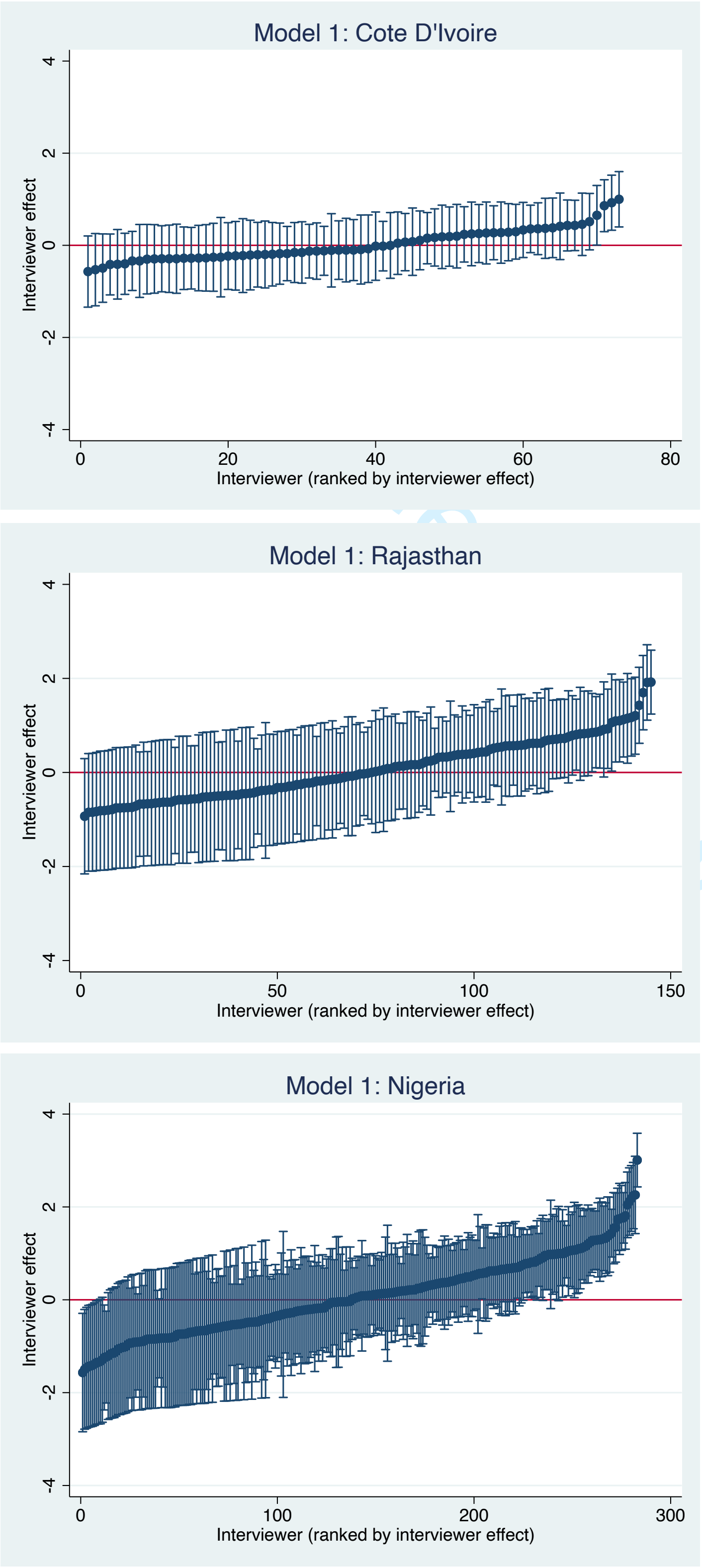

Figure 2. Intra-interviewer correlation by country and survey question, with 95% confidence intervals, adjusted for respondent and community characteristics (Model 1)

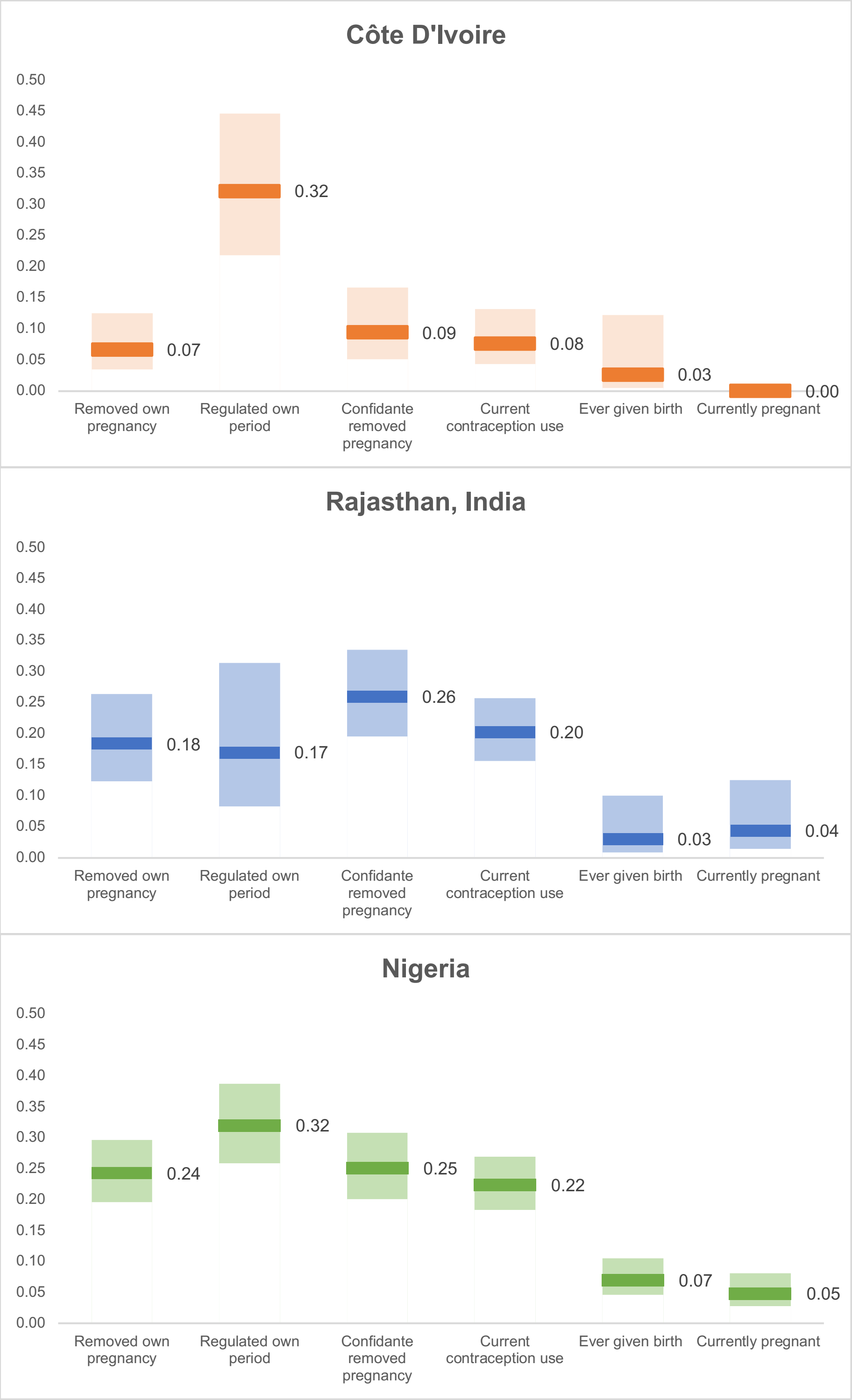

Appendix 1. Multilevel logit models with interviewer effects for PMA survey outcomes

	Côte d'Ivoire				Rajasthan, India				Nigeria			
	Level 2 variance	**IIC**	95% CI	LR test P value	Level 2 variance	**IIC**	95% CI	LR test P value	Level 2 variance	**IIC**	95% CI	LR test P value
**Abortion-related survey questions**												
**Ever removed a pregnancy**												
Null model	0.60	**0.15**	[0.10;0.23]		0.81	**0.20**	[0.14;0.28]		1.86	**0.36**	[0.31;0.42]	
Model 1	0.24	**0.07**	[0.03;0.13]	<0.001	0.74	**0.18**	[0.12;0.26]	<0.001	1.05	**0.24**	[0.20;0.30]	<0.001
Model 2	0.03	**0.01**	[0.00;0.11]	<0.001	0.60	**0.15**	[0.10;0.24]	0.15	0.94	**0.22**	[0.18;0.27]	<0.001
**Ever regulated a period**												
Null model	1.63	**0.33**	[0.23;0.45]		0.74	**0.18**	[0.10;0.32]		1.95	**0.37**	[0.31;0.44]	
Model 1	1.56	**0.32**	[0.22;0.45]	<0.001	0.67	**0.17**	[0.08;0.31]	<0.001	1.55	**0.32**	[0.26;0.39	<0.001
Model 2	1.10	**0.25**	[0.15;0.38]	0.03	0.45	**0.12**	[0.05;0.27]	0.08	1.51	**0.32**	[0.25;0.38]	0.30
**Confidante removed pregnancy**												
Null model	0.40	**0.11**	[0.06;0.18]		1.17	**0.26**	[0.20;0.34]		1.33	**0.29**	[0.24;0.35]	
Model 1	0.34	**0.09**	[0.05;0.17]	<0.001	1.15	**0.26**	[0.20;0.33]	<0.001	1.10	**0.25**	[0.20;0.31]	<0.001
Model 2	0.15	**0.04**	[0.01;0.12]	0.01	0.99	**0.23**	[0.17;0.31]	0.18	1.01	**0.23**	[0.19;0.29]	<0.001
**Less stigmatised survey questions**												
**Current contraceptive user**												
Null model	0.40	**0.11**	[0.07;0.17]		0.35	**0.10**	[0.07;0.13]		1.15	**0.26**	[0.22;0.31]	
Model 1	0.27	**0.08**	[0.04;0.13]	<0.001	0.83	**0.20**	[0.16;0.26]	<0.001	0.95	**0.22**	[0.18;0.27]	<0.001
Model 2	0.17	**0.05**	[0.02;0.10]	<0.001	0.73	**0.18**	[0.14;0.24]	0.07	0.92	**0.22**	[0.18;0.26]	0.07
**Ever given birth**												
Null model	0.43	**0.11**	[0.07;0.17]		0.06	**0.02**	[0.01;0.03]		0.37	**0.10**	[0.08;0.13]	
Model 1*	0.09	**0.03**	[0.01;0.12]	<0.001	0.10	**0.03**	[0.01;0.10]	<0.001	0.25	**0.07**	[0.05;0.11]	<0.001
Model 2	0.02	**0.01**	[0.00;0.00]	0.19	0.02	**0.00**	[0.00;0.85]	0.06	0.23	**0.06**	[0.04;0.10]	<0.001
**Currently pregnant**												
Null model	0.06	**0.02**	[0.00;0.00]		0.19	**0.05**	[0.02;0.12]		0.27	**0.08**	[0.05;0.11]	
Model 1**	0.00	**0.00**	[0.00;0.00]	<0.001	0.15	**0.04**	[0.01;0.13]	<0.001	0.17	**0.05**	[0.03;0.08]	<0.001
Model 2	0.00	**0.00**	[0.00;0.00]	0.29	0.11	**0.03**	[0.01;0.14]	0.41	0.17	**0.05**	[0.03;0.08]	0.08

Notes: Model 1 includes respondent variables (age, education, marital status, ever given birth, number of birth events, wealth, previous PMA experience) and community variables (region or state, rural / urban status, presence of facility providing abortion, monthly average number of abortions reported by facilities in community).
Model 2 includes respondent, community, and interviewer / interview-related variables (interviewer age, marital status, children, education, previous PMA staff, previous survey experience, comfort asking questions about abortion, respondent-interviewer familiarity and survey language).
*Excluding parity variables. **Excludes respondent marital status for Rajasthan. IIC = intra-interviewer correlation. CI = confidence intervals. LR test = Likelihood ratio test.

For peer review only

For peer review only - http://bmjopen.bmj.com/site/about/guidelines.xhtml

Appendix 2. Details of explanatory variables

Variable	Question	Categories	Re-code
**Respondent-Level Variables**			
**Age**	How old were you at your last birthday? [confirmed agreement with birth date]	Integer	- Treated as continuous
**Education**	What is the highest level of school you attended? [Only record formal schooling]	Never attended<br>Primary<br>Secondary<br>Higher Secondary [India only]<br>Tertiary / Higher / Graduate<br>No response	- 'Higher secondary' grouped with 'Secondary'<br>- No response treated as missing
**Marital status**	Are you currently married or living together with a man as if married?<br>[Probe: If no, ask whether the respondent is divorced, separated, or widowed.]	Currently married<br>Currently married, gauna not performed [India only]<br>Living with a man<br>Divorced / separated<br>Widow<br>No, never in union<br>No response	- 'Married, gauna not performed' coded as 'Currently married'<br>- Divorced/separated grouped with Widowed<br>- No response treated as missing.
**Ever given birth**	Have you ever given birth?	Yes<br>No<br>No response	- No response treated as missing.
**Number of birth events**	How many times have you given birth?	Integer	- No response treated as missing
**Wealth**	Household wealth index questions administered in household questionnaire	Lowest quintile<br>Lower quintile<br>Middle quintile<br>Higher quintile<br>Highest quintile	- Pre-constructed in dataset
**Previous PMA participation**	Has the respondent previously participated in PMA 2020 surveys?<br>[completed by interviewer]	Yes<br>No<br>Do not know<br>No response	- Do not know and No response coded as missing
**Community-Level Variables**			
**Region / State**	Enumeration area categorised based on sampling frame	Vary by country	- Pre-constructed in dataset
**Urban /rural**		Urban<br>Rural	- Pre-constructed in dataset
**Abortion care facility in community**	Does this facility provide post-abortion care for pregnancies of 12 weeks or less / more than 12 weeks?<br>AND<br>Does this facility provide abortion for pregnancies of 12 weeks or less / more than 12 weeks?	Yes<br>No<br>Do not know<br>No response	- Binary variable coded 1 if any facilities in or serving the enumeration area answered yes to providing abortion or post-abortion care for either gestational age group, and 0 if all facilities answered no to all questions. Public hospitals were excluded as hospital catchment areas can be very large, so an enumeration area may not have abortion care available if it only has a public hospital serving the area.
**Monthly mean number of abortions**	In an AVERAGE / the LAST month, how many post-abortion care patients would you	Integer variable<br>-88 for do not know<br>-99 for no response.	- Average of the number of post-abortion care services in the last month and in an average month was calculated for each

For peer review only

	estimate are/were treated as INPATIENTS / OUTPATIENTS in this facility as a whole? Please remember to include all patients treated for extremely serious or minor abortion complications, whether the abortions were spontaneous or induced. Ensure provider responds from memory and does NOT reference the log book.<br><br>In an AVERAGE / the LAST month, how many abortions are/were provided?		facility (to get a more accurate average that accounts for possible seasonal variation).<br>- Average of the number of abortion services in the last month and in an average month was calculated for each facility (to get a more accurate average that accounts for possible seasonal variation).<br>- The average numbers of abortions and post-abortion care services were summed per facility, and then per cluster to give a cluster total of the number of abortions / post-abortion care services reported by facilities per month on average.
**Interview-level Variables**			
**Interviewer-respondent familiarity**	How well acquainted are you with the respondent? [completed by interviewer]	Very well acquainted<br>Well acquainted<br>Not well acquainted<br>Not acquainted	- Very well acquainted and well acquainted grouped together to give a categorical variable with three groups.
**Survey language**	Administrative data recorded by interviewer	Varied by country	- Languages used in <20 interviews grouped into ‘Other’
**Interviewer-level Variables**			
**Number of respondents**	-	-	- Constructed by summing the number of interviews completed per interviewer
**Age**	How old were you at your last birthday?	Integer	- Treated as continuous
**Marital status**	Select your marital status	Never Married<br>Currently Married<br>Living with a partner<br>Divorced<br>Widowed	- Coded as binary variable where 1 is married (‘Currently Married’) and 0 is all other categories
**Children**	Do you have any children?	Yes<br>No	-
**Education**	What is the highest level of education that you have completed?	Primary School<br>Secondary School<br>Higher Secondary [India only]<br>University<br>Technical School<br>Graduate School (Masters)<br>Graduate School (Doctoral)<br>[Côte D’Ivoire only]	- Recoded into a binary variable where 1 is "Primary/Secondary" and 2 is "Technical / Uni graduate and above"
**Existing staff**	Have you ever completed a round of data collection for PMA2020 before the round you are preparing for now?	Yes, I am a returning PMA2020 staff member<br>No, this will be my first round of data collection	-
**Previous experience**	Do you have any previous survey experience?	Yes<br>No	-

For peer review only

**Comfort asking about abortion**	Are you comfortable asking respondents questions about abortion?	Yes, completely<br>Somewhat<br>No	-	Binary variable coded as 1 if interviewer answered 'yes completely' and 0 if they answered 'somewhat' or 'no'

For peer review only

STROBE Statement—Checklist of items that should be included in reports of ***cross-sectional studies***

	Item No	Recommendation	Page No
**Title and abstract**	1	(*a*) Indicate the study's design with a commonly used term in the title or the abstract	Page 1
		(*b*) Provide in the abstract an informative and balanced summary of what was done and what was found	Page 1
**Introduction**			
Background/rationale	2	Explain the scientific background and rationale for the investigation being reported	Page 3-4
Objectives	3	State specific objectives, including any prespecified hypotheses	Page 5
**Methods**			
Study design	4	Present key elements of study design early in the paper	Page 5-6,7
Setting	5	Describe the setting, locations, and relevant dates, including periods of recruitment, exposure, follow-up, and data collection	Page 5-6
Participants	6	(*a*) Give the eligibility criteria, and the sources and methods of selection of participants	Page 5-6
Variables	7	Clearly define all outcomes, exposures, predictors, potential confounders, and effect modifiers. Give diagnostic criteria, if applicable	Page 6-7, Appendix 1
Data sources/ measurement	8*	For each variable of interest, give sources of data and details of methods of assessment (measurement). Describe comparability of assessment methods if there is more than one group	Page 6-7, Appendix 1
Bias	9	Describe any efforts to address potential sources of bias	Page 8
Study size	10	Explain how the study size was arrived at	Page 5-6
Quantitative variables	11	Explain how quantitative variables were handled in the analyses. If applicable, describe which groupings were chosen and why	Page 6-7, Appendix 1
Statistical methods	12	(*a*) Describe all statistical methods, including those used to control for confounding	Page 8
		(*b*) Describe any methods used to examine subgroups and interactions	Page 8
		(*c*) Explain how missing data were addressed	Page 8
		(*d*) If applicable, describe analytical methods taking account of sampling strategy	Page 8
		(*e*) Describe any sensitivity analyses	Page 8
**Results**			
Participants	13*	(a) Report numbers of individuals at each stage of study—eg numbers potentially eligible, examined for eligibility, confirmed eligible, included in the study, completing follow-up, and analysed	n/a
		(b) Give reasons for non-participation at each stage	n/a
		(c) Consider use of a flow diagram	n/a
Descriptive data	14*	(a) Give characteristics of study participants (eg demographic, clinical, social) and information on exposures and potential confounders	Page 19/20 Tables 1 & 2
		(b) Indicate number of participants with missing data for each variable of interest	Page 19/20 Tables 1 & 2
Outcome data	15*	Report numbers of outcome events or summary measures	Page 20, Table 2

For peer review only

Main results	16	(*a*) Give unadjusted estimates and, if applicable, confounder-adjusted estimates and their precision (eg, 95% confidence interval). Make clear which confounders were adjusted for and why they were included	Page 23, Table 3
		(*b*) Report category boundaries when continuous variables were categorized	N/A
		(*c*) If relevant, consider translating estimates of relative risk into absolute risk for a meaningful time period	N/A
Other analyses	17	Report other analyses done—eg analyses of subgroups and interactions, and sensitivity analyses	Page 8
**Discussion**			
Key results	18	Summarise key results with reference to study objectives	Page 10
Limitations	19	Discuss limitations of the study, taking into account sources of potential bias or imprecision. Discuss both direction and magnitude of any potential bias	Page 10-11
Interpretation	20	Give a cautious overall interpretation of results considering objectives, limitations, multiplicity of analyses, results from similar studies, and other relevant evidence	Page 10-11
Generalisability	21	Discuss the generalisability (external validity) of the study results	Page 11
**Other information**			
Funding	22	Give the source of funding and the role of the funders for the present study and, if applicable, for the original study on which the present article is based	Page 13

*Give information separately for exposed and unexposed groups.

Note: An Explanation and Elaboration article discusses each checklist item and gives methodological background and published examples of transparent reporting. The STROBE checklist is best used in conjunction with this article (freely available on the Web sites of PLoS Medicine at http://www.plosmedicine.org/, Annals of Internal Medicine at http://www.annals.org/, and Epidemiology at http://www.epidem.com/). Information on the STROBE Initiative is available at www.strobe-statement.org.

For peer review only

BMJ Open

Interviewer effects on abortion reporting: a multilevel analysis of household survey responses in Côte d’Ivoire, Nigeria and Rajasthan, India

Journal:	*BMJ Open*
Manuscript ID	bmjopen-2020-047570.R1
Article Type:	Original research
Date Submitted by the Author:	31-Mar-2021
Complete List of Authors:	Footman, Katharine; The London School of Economics and Political Science, Social Policy
**Primary Subject Heading**:	Research methods
Secondary Subject Heading:	Reproductive medicine
Keywords:	Demography < TROPICAL MEDICINE, STATISTICS & RESEARCH METHODS, REPRODUCTIVE MEDICINE, PUBLIC HEALTH

SCHOLARONE™
Manuscripts

I, the Submitting Author has the right to grant and does grant on behalf of all authors of the Work (as defined in the below author licence), an exclusive licence and/or a non-exclusive licence for contributions from authors who are: i) UK Crown employees; ii) where BMJ has agreed a CC-BY licence shall apply, and/or iii) in accordance with the terms applicable for US Federal Government officers or employees acting as part of their official duties; on a worldwide, perpetual, irrevocable, royalty-free basis to BMJ Publishing Group Ltd ("BMJ") its licensees and where the relevant Journal is co-owned by BMJ to the co-owners of the Journal, to publish the Work in this journal and any other BMJ products and to exploit all rights, as set out in our licence.

The Submitting Author accepts and understands that any supply made under these terms is made by BMJ to the Submitting Author unless you are acting as an employee on behalf of your employer or a postgraduate student of an affiliated institution which is paying any applicable article publishing charge ("APC") for Open Access articles. Where the Submitting Author wishes to make the Work available on an Open Access basis (and intends to pay the relevant APC), the terms of reuse of such Open Access shall be governed by a Creative Commons licence – details of these licences and which Creative Commons licence will apply to this Work are set out in our licence referred to above.

Other than as permitted in any relevant BMJ Author's Self Archiving Policies, I confirm this Work has not been accepted for publication elsewhere, is not being considered for publication elsewhere and does not duplicate material already published. I confirm all authors consent to publication of this Work and authorise the granting of this licence.

For peer review only

Title: Interviewer effects on abortion reporting: a multilevel analysis of household survey responses in Côte d'Ivoire, Nigeria and Rajasthan, India

Author: Katharine Footman, Department of Social Policy, London School of Economics and Political Science, Houghton Street, London, WC2A 2AE. K.footman@lse.ac.uk

Abstract:

Objectives: The analysis aimed to assess the scale of interviewer effects on abortion survey responses, to compare interviewer effects between different question wordings and between direct and indirect approaches, and to identify interviewer and interview characteristics that explain interviewer effects on abortion reporting.
Setting: 2018 Performance Monitoring for Action (PMA) nationally representative household surveys from Côte d'Ivoire, Nigeria and Rajasthan, India
Participants: Survey data from 20,016 interviews with reproductive age (15-49) women, selected using multi-stage stratified cluster sampling. Data from self-administered interviewer surveys and from a sample of health service delivery points that serve the female survey participants were also included.
Primary outcome measures: Outcomes were the respondent's own experience of ever 'removing a pregnancy', their closest confidante's experience of pregnancy removal and the respondent's own experience of period regulation.
Results: Substantial interviewer effects were observed, ranging from 7% in Côte d'Ivoire to 24% in Nigeria for pregnancy removal. Interviewer effects for survey questions that were designed to ask about abortion in a less stigmatising way were either similar to (9-26% for confidante-reporting) or higher than (17-32% for a question about period regulation) the pregnancy removal question. Interviewer and interview characteristics associated with abortion reporting included respondent-interviewer familiarity, the language of interview, and the interviewer's comfort asking questions about abortion.
Conclusion: This study highlights that questions designed to be less stigmatising may increase interviewer effects due to lower comprehension among respondents. Further work is needed to assess question wordings for different contexts. Selecting and training interviewers to ensure comfort asking questions about abortion is important for reproductive health surveys. Challenges for the use of 'insider' interviewers and the management of surveys in countries with high linguistic diversity are also identified.

For peer review only

Strengths and limitations of the study:

- This is one of the first studies of interviewer effects on abortion reporting in low- and middle-income countries and it is the first to assess the recently developed Performance Monitoring for Action (PMA) survey abortion module.
- The analysis extends existing knowledge about interviewer effects for abortion as the PMA module included new survey questions, and meta-data allowed interview and interviewer characteristics to be assessed, including respondent-interviewer familiarity.
- It was not possible to entirely separate interviewer effects from area affects, so interviewer effects may be over-estimated, but fixed respondent and community characteristics were included in the analysis to account for area effects to some extent.
- It is not possible to tell whether characteristics associated with higher reporting reflect an association with prior abortion experience or with willingness to report an abortion.

For peer review only

Introduction

Only 37% of women of reproductive age live in countries where their legal right to access abortion does not depend on their reason for ending the pregnancy.[1] Almost half of the 73 million abortions that occur each year are unsafe due to these restrictions on access to safe abortion.[2] Unsafe abortions are defined by the WHO as abortions carried out either by a person lacking the necessary skills or in an environment that does not conform to minimal medical standards, or both.[3] Despite its importance as a human rights and public health issue, there is limited evidence about abortion in most countries.[4] Abortion data are needed to inform policy and advocacy: evidence about the incidence and impacts of unsafe abortion has played an important role in the liberalisation of abortion laws.[5–7] Strategies to improve equitable access to safe abortion also require high quality data about where and how women are seeking abortion, and which population sub-groups are most affected by barriers to safe abortion care.[8,9]

Unsafe abortion disproportionately affects women in low- and middle-income countries (LMICs) (86% of all abortions[10] and 97% of unsafe abortions[1]), largely driven by laws and policies that restrict access to safe abortion. Data about abortion is often particularly limited in these contexts because health records exclude illegal, informal and self-managed abortions.[11] Interviewer-administered household surveys tend to be key sources of demographic, reproductive and public health data in LMICs.[12–14] However, concerns about under-reporting mean that survey data about abortion are rarely used and poorly trusted[4,15]: studies from high-income countries that compare survey self-reports to health records suggest that 35-80% of respondents accurately report abortions in surveys.[16–19]

Efforts to improve the availability of abortion data have focussed on estimating abortion incidence using indirect methods[4] or reducing under-reporting in surveys. Some surveys have (unsuccessfully) attempted to reduce under-reporting in direct questions by grouping abortions and miscarriages together [4], adding a filter question about previous unwanted pregnancies[20], or asking for a full pregnancy history.[21,22] A recent review[4] recommended additionally asking women directly if they have used medications to bring on their periods, due to recent changes in the way abortion methods are used and conceptualised. Performance Monitoring for Action (PMA) surveys now include this approach by asking participants about experiences of 'period regulation'.[23] Methods to reduce under-reporting in surveys through indirect questions have produced mixed results[4,18,24] and have a number of flaws that limit the utility of the data they produce. For example, random response methods[25–27] and list experiments[28–33] do not produce individual-level data, have limited precision, and do not permit follow-on questions about sources of abortion and their safety, the process of abortion-seeking or issues with access to abortion care. Confidante reporting[34–36], where the respondent is asked about abortions in their social networks, rather than their own abortions, has also been used in PMA surveys.[11] Confidante reporting can reduce the role of stigma in abortion reporting, though it relies on respondents being aware of their friends' abortions and being willing to report them.[4] Finally, survey researchers have also attempted to reduce under-reporting by limiting the impact of the interviewer through self-administered surveys[37,38], phone interviews[39,40], or audio computer-assisted self-interviewing (ACASI).[24,26] However, these methods can be less feasible in contexts where there is lower literacy, technology use and phone ownership.[4,41] Interviewers therefore continue to play a pivotal role in the quality of survey data, particularly in low-resource settings: they make contact with respondents, explain the purpose of the survey, gain consent, ask questions, record answers, and motivate respondents throughout the interview.[42]

Abortion questions have often been excluded from household surveys for these methodological (and some political) reasons[8], but there are increasing efforts to address this gap. For example, several countries now include direct questions about abortion in Demographic and Health Surveys (DHS)[43,44], and an abortion module was recently included in three countries' PMA (Performance Monitoring for Action) surveys.[45] These surveys offer an opportunity to expand the availability of abortion data. However, more work is needed to explore data quality issues beyond under-reporting, to assess which questions and methods can improve the quality of abortion data from household surveys, and to identify which interviewer and interview characteristics affect abortion reporting.

For peer review only - http://bmjopen.bmj.com/site/about/guidelines.xhtml

Interviewers can impact the accuracy of survey responses due to their observed characteristics, such as age or gender, or role-related characteristics, such as the way they read questions, probe or provide instructions.[46] Characteristics of the interaction itself can also impact survey responses, for example whether there is pre-existing familiarity between interviewer and respondent[47,48], and the effects of observed interviewer characteristics (such as ethnicity or age) can be moderated by characteristics of the respondent.[49] However, the characteristics of interviewers and interviews that may impact reporting of abortion in surveys has not previously been explored.

Interviewer effects vary by survey question, but are more likely to cause measurement error for questions that are complex, sensitive, non-factual, open-ended or evoke emotional responses.[50–52] The interviewer effect on the variance of the mean for a survey item can be expressed as $1+\rho_{int}(m-1)$, where m is the average number of interviews completed per interviewer and the interviewer effect ρ_{int} is the intra-class correlation, or intra-interviewer correlation (IIC) of the survey responses.[53] Interviewer effects can range from 1-12% of the variance in survey responses in face-to-face surveys, with most being below 2% [54,55], but even small interviewer effects can have a significant impact on survey data quality, especially if each interviewer collects a large number of responses.[56]

There is a large body of research on interviewer effects in high-income countries[49] but few studies in LMICs[41], despite the increased importance of this topic in these contexts given higher dependence on interviewers for data collection.[12,48] There have also been very few studies on interviewer effects for reproductive health surveys and this evidence has been mixed. A review of the DHS in Indonesia and the Philippines found low (1%) interviewer effects for questions about contraceptive use[57] while analysis of the DHS in Kenya and Malawi found higher interviewer effects for contraceptive use (ranging between 3-25%), with interviewer gender, marital and fertility status being important covariates of response patterns.[58] The remaining limited literature on interviewer effects in reproductive health surveys in LMICs has focused on the gender of interviewer[59–62] and respondent-interviewer familiarity.[13,14,48,63–65] Only one study has assessed interviewer effects on survey questions about abortion in LMICs, using a cross-classified multilevel model to separate interviewer effects and area effects in 22 DHS surveys. The study found large interviewer effects ranging from 0.2% to 50%, that interviewer effects were stronger for questions about 'abortion' than 'any termination', and that interviewer effects were greater than area effects.[41] However, the interviewer and interview characteristics that might explain these effects could not be assessed in this DHS analysis, due to a lack of meta-data about interviewer and interview characteristics in most DHS surveys.

Building on these findings[41], the present analysis is the first to assess interviewer effects for abortion responses in PMA survey data. PMA surveys used an extended abortion module to collect nationally- and state-representative data about abortion incidence and safety in 2018 in Nigeria, Cote d'Ivoire, and the state of Rajasthan in India, and expanded on previous survey methodologies for abortion by including new questions designed to increase reporting.[11,23,45] This analysis therefore extends the limited literature on interviewer effects for abortion by assessing and comparing these new survey questions. In addition, PMA surveys include meta-data about interviewer and interview characteristics, so this analysis is the first to assess the characteristics that may explain interviewer effects on abortion questions. PMA surveys also include abortion service delivery data from the area, allowing abortion reporting to be separated from abortion incidence to some extent in this analysis. Finally, the PMA model of using local interviewers enables further exploration of the role of respondent-interviewer familiarity in the context of interviewer effects for abortion.

Aim

This analysis was intended to assess the scale of interviewer effects on abortion survey responses using PMA data. The aims of the analysis were to compare interviewer effects for abortion reporting: (1) between questions that employ different language to refer to abortion; (2) between direct and indirect approaches to abortion measurement; and (3) with questions about less stigmatised topics. Finally, the analysis aimed (4) to identify interviewer and interview characteristics that explain interviewer effects on abortion reporting.

For peer review only - http://bmjopen.bmj.com/site/about/guidelines.xhtml

The study's hypotheses were that interviewer effects for less stigmatized topics will have lower interviewer effects than questions about abortion, and that questions designed to ask about abortion in a less stigmatizing way (indirectly and using different language to refer to abortion) will have lower interviewer effects. The study also hypothesized that interviewer effects will be explained by interviewer and interview characteristics, including observable interviewer characteristics, interviewer-respondent familiarity and language of interview.

Methods

Contexts

This analysis used nationally representative PMA survey data collected in Nigeria[66,67], Côte d'Ivoire[68,69] and Rajasthan state, India[70,71] in 2018, as these surveys included the extended abortion module. The three countries have varying abortion laws, with abortion being broadly legal in India since 1971, while abortion is allowed only to save a woman's life in Côte d'Ivoire and in Nigeria at the federal level. Abortion stigma is prevalent in each of the three contexts, though it can be more evident in countries with more restrictive abortion laws.[72] The proportion of abortions that are most unsafe (involving non-recommended methods and non-clinical providers) was estimated at 62% in Côte d'Ivoire, 63% in Nigeria and 31% in Rajasthan.[45] The most recent indirect estimates suggest the majority (73%) of abortions in India are safe medication abortions occurring outside health facilities, while only 5% of abortions involve informal unsafe methods.[73] In Nigeria, use of medication abortions from pharmacies is also increasingly common[74,75], but in both the West African countries, unsafe methods are still most commonly used to end a pregnancy.[76–78]

Data

PMA is a multi-country project that conducts frequent reproductive health surveys in nine countries in sub-Saharan Africa and Asia. PMA survey methodology is described in detail elsewhere.[79] In brief, the surveys sample reproductive age women, households and health service delivery points using multi-stage stratified cluster sampling. Primary sampling units (PSU) are selected using probability proportional to size procedures, with stratification by urban-rural status (in Côte d'Ivoire and Rajasthan) or state (Nigeria). In each PSU, households are mapped and listed by interviewers and 35 (40 in Lagos state, Nigeria) households are randomly selected to be invited for a face-to-face interview. Interviewers conduct the informed consent process. The proportion of selected, occupied households that consented to be take part in a household-level interview was 98% and the proportion of females within participating households who consented to take part was 98-99% in each country.

A household interview is conducted, and all females age 15-49 in each household are interviewed using a structured questionnaire that includes demographic characteristics; pregnancy and fertility preferences; contraception; sexual activity; and menstrual hygiene. In 2018, researchers added an abortion module in Round 2 in the survey of Côte d'Ivoire, Round 5 of Nigeria, and Round 4 of Rajasthan, India. A survey is also conducted at a sample of health service delivery points (SDPs) that serve the representative sample of reproductive age women. The SDP sample includes the public facilities at each level of the healthcare system that serve each PSU, and up to three private SDPs located in each PSU. The SDP questionnaire collects data on the availability and volumes of a range of health services, including abortion and post-abortion care for the round of data collection under analysis. Additionally, before data collection begins, interviewers self-administer a staff survey with questions about their demographic characteristics and previous experience.

PMA surveys are repeated every 6-12 months with a new sample drawn at each round, so frequent, rapid data collection is facilitated using mobile data collection and 'resident enumerators' who live in the enumeration area. Interviewers may therefore be known or familiar to respondents, particularly as they are retained between data collection rounds whenever possible.[14] Interviewers are typically women, over the age of 21, holding at least secondary school education, familiar with mobile phones, and with no affiliation to the local health system.[14]

For peer review only

Measures

The abortion module used four questions to gather data on experiences of abortion. First, the confidante method was used, where women are asked to report the abortions of a set number of close friends, defined by PMA as “women whom you share secrets with and who also share theirs with you”. In this survey, respondents were asked about their two closest friends but only data for the participant’s closest confidante were used in this analysis. Participants were asked whether each of their confidantes had ever done something to 1) “remove a pregnancy” and/or to 2) “regulate a period” when she was pregnant or worried she was pregnant. Participants could answer ‘yes I am certain’, ‘yes I think so’, ‘no’, ‘don’t know’ or ‘no response’, and the variable was dichotomised by grouping positive responses and treating ‘no response’ as missing (<1% of responses). Respondents were then asked whether they themselves had done something to 1) “remove a pregnancy” and/or 2) “regulate a period” when they were pregnant or worried that they were pregnant. Respondents could answer ‘yes’, ‘no’ or ‘no response’ and non-responses were treated as missing (<1% of responses). For each question, the interviewer was instructed to “*probe to confirm whether the pregnancy removal (or period regulation) was successful”* and unsuccessful attempts were not recorded.

In this analysis, the respondent’s own experience of ever ‘removing a pregnancy’ was considered the main measure of a previous abortion. Interviewer effects for this outcome were compared to the abortion questions designed to be less stigmatising as they avoid personal disclosure[11] or allow for different understandings of abortion[4,80,81]: their closest confidante’s experience of pregnancy removal and the respondent’s own experience of period regulation. The interviewer effects for these abortion questions were then compared to topics considered to be less stigmatised: first, whether the respondent or their partner were ‘currently doing something or using any method to delay or avoid getting pregnant’ (their current contraceptive use); second, whether the respondent reported they had ever given birth, and; third, whether the respondent reported they were currently pregnant.

Explanatory variables were selected based on previous literature about factors associated with abortion reporting[17,19,38,39,82], contraceptive use reporting[63–65], and abortion incidence[10,17,19] as well as the researchers’ hypotheses about the potential impacts of interviewer and interview characteristics on abortion reporting. Explanatory variables included respondent, community, interview and interviewer characteristics (see Appendix 1 for further detail). At the respondent-level, explanatory variables included respondent age, education status, marital status, parity (ever given birth and number of births), household wealth, and previous PMA participation. At the community-level, explanatory variables were derived from the individual, household and service delivery data, including urban/rural status, region or state, whether the enumeration area had a SDP that provided abortion or post-abortion care, and the monthly volume of abortion services reported by these SDPs. Interview characteristics included respondent-interviewer familiarity (as recorded by the interviewer for each respondent) and survey language. Interviewer characteristics included age, marital status, education status, whether they had children, previous involvement in PMA data collection, previous survey experience outside of PMA, number of respondents interviewed and self-reported comfort asking questions about abortion. Comfort asking about abortion was measured using the question: “Are you comfortable asking respondents questions about abortion?” with response options of ‘Yes, completely’, ‘Somewhat’ and ‘No’. Due to high levels of reported comfort asking questions about abortion, interviewers were considered to be comfortable only if they answered ‘Yes, completely’ as detailed in Appendix 1.

The staff survey was not completed in Nigeria in 2018 so interviewer characteristics were not available for all three countries. However, Nigeria was included in the analysis because the available data could still be used to address the first three aims of the analysis, and interview characteristics data were also available.

Analysis

Descriptive analysis of the outcomes of interest and the potential explanatory variables was conducted to assess their distributions. Bivariate logistic regression analysis was used to assess associations between

For peer review only - http://bmjopen.bmj.com/site/about/guidelines.xhtml

potential explanatory variables and the odds of reporting removing a pregnancy, period regulation and a confidante's pregnancy removal.

Multi-level logistic regression models with an interviewer random intercept were then sequentially developed to assess the variance in outcomes within- and between-interviewers. Multi-level modelling was used to account for unmeasured interviewer characteristics and non-random allocation of respondents to interviewers, and to address the correlated error terms resulting from multiple respondents being interviewed by the same person and living within the same community (as there is one interviewer per PSU in PMA surveys).[56] Explanatory variables were added to the model sequentially: Model 0 included only the interviewer random effects term, Model 1 also included respondent and community characteristics, and Model 2 additionally included interview and interviewer characteristics.

Model 0 was estimated according to the equation: $\log(\pi_{ij} / 1-\pi_{ij}) = \beta_0 + u_j$ where π is the probability of reporting having ever removed a pregnancy; β_0 is the log-odds of reporting having removed a pregnancy when u=0 (i.e. for the average interviewer); and the addition of u_j gives the intercept for interviewer j, or the interviewer effect. Models 1 and 2 were estimated using the equation: $\log(\pi_{ij} / 1-\pi_{ij}) = \beta_0 + \beta_1 x_{1ij} + \beta_2 x_{2ij} + \ldots + \beta_k x_{kij} + u_j$ where β_0 is the log-odds of reporting having removed a pregnancy when x=0 and u=0; $\beta_1 x_{ij}$ is the effect of a 1-unit change in x on the log-odds, holding constant all other explanatory variables and the interviewer effect u; and u_j is the interviewer effect for interviewer j. The models assume that $(u_j \sim N(0,\sigma^2_u))$, meaning the level 2 residuals u_j are assumed to be independent and to follow normal distributions with means of zero. The variance of u_j (σ^2_u) is the level 2 residual variance and was used to calculate the intra-interviewer correlation (IIC) using the equation: $IIC = \sigma_u^2 / \sigma_u^2 + \sigma_e^2$ where σ_e^2 (the level 1 residual variance) is equal to 3.29 for a logit model.[83] The IIC is therefore the proportion of total variance that can be explained by interviewer effects, and the terms 'IIC' and 'interviewer effects' are used interchangeably in the Results.

Analyses were conducted for each country individually. Likelihood ratio tests were used at each stage to assess whether the multi-level model offered a significant improvement on a simple logistic model, and to assess whether the addition of variables improved the fit of the multi-level model. Simple fixed effects logistic regression models were also run at each stage and coefficients were compared between the simple and multi-level models to check for any substantial differences. In descriptive and bivariate analyses, survey weights were used and the complex sampling design was accounted for using the Taylor linearization method. In the multi-level models, clustering and stratification were adjusted for through the inclusion of the interviewer random intercept and inclusion of strata (urban/rural and state) as covariates. The multilevel analysis was not weighted for simplicity, but the weights variable was included as a coefficient in Model 2 as a sensitivity analysis. All other coefficients remained similar to the original model and the weight variable was non-significant in each country. The estimation procedure was maximum likelihood estimation using adaptive quadrature with seven integration points. To assess that seven integration points was adequate, the model was refitted with a larger number of integration points (up to 40) to assess that model parameters were substantially similar[83], and this sensitivity analysis confirmed that the coefficients were stable. Complete records analysis was conducted.

All analyses were conducted in Stata 15.1. Statistical significance was determined using an alpha of 0.05, but coefficients significant at the 0.10 level were also noted.

Ethics

As this secondary analysis used de-identified and administrative data, the analysis was exempted from review by the London School of Economics and Political Science and the Johns Hopkins Bloomberg School of Public Health ethics review boards.

Patient and public involvement

For peer review only

No patients were involved in the development of the research questions, analysis or dissemination.

Results

The interview and interviewer characteristics are shown in Table 1 (respondent and community characteristics are in Appendix 2). There were 73 interviewers in Côte D'Ivoire, 145 in Rajasthan and 285 in Nigeria. Interviewer survey data were only available in Côte d'Ivoire and Rajasthan and in both surveys, all interviewers were female. Interviewers were older in Côte d'Ivoire and a lower proportion were married (20%) than in Rajasthan (80%). More interviewers in Côte d'Ivoire had technical or graduate education (86% vs 51%) and had previous survey experience (90% vs 19%). Almost all interviewers reported that they felt completely comfortable asking questions about abortion in both countries. There were no significant differences in the characteristics of interviewers who reported being completely comfortable asking about abortion in Côte D'Ivoire but in Rajasthan interviewers with technical or graduate education (94% vs 100%, p=0.049) and interviewers without children (92% vs 100%, p=0.007) were slightly less likely to report being completely comfortable compared to interviews with only primary / secondary education or with children (data not shown).

The prevalence of reporting a previous abortion for the respondent themselves or their closest confidante is presented in Table 2. Self-reports of having ever removed a pregnancy were highest in Côte d'Ivoire (19%) and Nigeria (15%) compared to Rajasthan (7%). Confidante-reporting was higher than self-report in Rajasthan and Nigeria, but not in Côte d'Ivoire. Reports of ever having regulated a period were significantly lower than reports of ever having removed a pregnancy in each setting, both for self-reporting and for confidante-reporting.

Figures 1 and 2 illustrate the interviewer effects in each country. The caterpillar plots in Figure 1 show the mean log-odds for reporting ever removing a pregnancy to each interviewer, ordered by size, after adjusting for respondent and community characteristics (Model 1). In Figure 2, the interviewer effects for each of the questions is shown for each country (Model 1). Interviewer effects for the pregnancy removal question were substantial in each country but were largest in Nigeria and Rajasthan. Interviewer effect accounted for 7% of the variance in the odds of reporting removing a pregnancy in Côte d'Ivoire, 18% in Rajasthan and 24% in Nigeria. The interviewer effect was higher for the question about period regulation than pregnancy removal in the West African countries, particularly in Côte d'Ivoire (Figure 2). The question about the closest confidante's pregnancy removal had similar interviewer effects to the question about the respondent's own pregnancy removal in each country. Interviewer effects were generally lower for the questions about previous births (ranging from 3-7%) and current pregnancy (0-5%), compared to the questions about abortion. However, interviewer effects for the question about current contraceptive use were similar in size (8-22%) to the questions about abortion.

Table 3 presents Model 2 for the odds of reporting removing a pregnancy in each country, adjusting for respondent, community, interviewer and interview characteristics (full model is shown in Appendix 3). Interviewer effects were still substantial in Model 2 for Rajasthan and Nigeria, suggesting that there are other factors not included in the model that may explain the variance between interviewers in these countries (particularly for Nigeria, where it was not possible to include interviewer characteristics in the model). The IIC for each of the models is presented in Appendix 4. Adjusting for interview and interviewer characteristics in Model 2 did reduce the size of the interviewer effects for most countries and questions but did not significantly improve the fit of the model compared to Model 1, particularly for Rajasthan.

In both Rajasthan and Nigeria, respondents who were not well acquainted with the interviewer had significantly lower odds of reporting a pregnancy removal compared to respondents who were well or very well acquainted. However, respondents who were not at all acquainted did not have significantly different odds from those who were well acquainted. There was no significant association with respondent-interviewer familiarity in Côte d'Ivoire, where almost all respondents were not at all familiar with the

For peer review only

interviewer. Survey language was significantly associated with the odds of reporting a pregnancy removal in the West African countries, but not in Rajasthan where almost all interviews were conducted in Hindi.

In Rajasthan, none of the interviewer characteristics were significant. In Côte d'Ivoire the interviewer's parity, education level and comfort asking questions about abortion were significant at the 0.1 level.

In each country (Appendix 3), respondent characteristics were significantly associated with the odds of reporting a pregnancy removal, in line with previous studies about sub-group differences in abortion reporting[17,19] and abortion incidence.[10,17,19] Broadly, respondents who were older, with more formal education and in a higher wealth quintile were more likely to report a pregnancy removal, though there were some differences between countries. Abortion service availability and volumes were only significant in Nigeria, where the availability of a clinic providing abortion was associated with significantly higher odds of reporting a pregnancy removal, though surprisingly the association with the facility-reported monthly number of abortions was negative.

Discussion

Interviewer-administered household surveys will continue to be an important source of health information in LMICs for the foreseeable future due to limitations of formal health records[11] and challenges with alternative modes of survey data collection in low-resource settings.[12] A greater understanding of interviewer effects for abortion questions in household surveys can support efforts to improve the quality of these data and increase the availability of much-needed evidence about abortion.

This analysis identified substantial interviewer effects for abortion reporting in three PMA survey countries, ranging from 7% in Côte d'Ivoire to 24% in Nigeria for ever having removed a pregnancy. Contrary to the original study hypotheses, questions designed to ask about abortion in a less stigmatising way did not have lower interviewer effects, and in fact interviewer effects were higher for a question about period regulation than pregnancy removal in Côte d'Ivoire and Nigeria. Although interviewer effects were considerably smaller for less stigmatised topics such as previous births and current pregnancies as hypothesised, interviewer effects for contraceptive use were similar in scale to questions about abortion. The interviewer characteristics that affect abortion reporting could not be fully explained by the available data, but significant interview and interviewer characteristics identified in this analysis were: respondent-interviewer familiarity, the language of interview, and the interviewer's comfort asking questions about abortion.

Interviewer effects observed in this study were higher than average estimates from surveys in high-income countries, which tend to be below 2% and vary from 1-12% (though these estimates are not for abortion measures).[54,55] Higher variance between interviewers does not necessarily indicate lower validity of abortion reporting, as each interviewer could discourage true reporting equally, but evidence that less sensitive questions have lower interviewer variance suggests that lower variance may be a sign of higher validity.[41] Interviewer effects can also affect survey data quality by increasing the variance of estimates[56], so understanding, minimising and accounting for interviewer effects is important.

Interviewer effects for questions about contraceptive use in PMA were as high as the abortion questions. The evidence on interviewer effects for contraceptive use questions in DHS data is limited and mixed[57,58] but inaccuracies in self-reports of contraceptive use have been noted in several studies.[84–86] Contraceptive use can be highly sensitive and is often covert.[87] The topic of survey data quality for contraceptive use has been neglected[84], but the high interviewer effects for contraceptive use observed in this analysis suggest that this issue warrants further attention. Unlike abortion, contraceptive use is a widely collected survey item, and its measurement was the original aim of investment in PMA.[79] Though abortion faces the additional issue of under-reporting in surveys, this finding does raise questions about the frequent exclusion of abortion from reproductive health surveys on methodological grounds, since some of the same quality issues exist for other common reproductive health topics.

For peer review only

To improve the quality of survey data on abortion, further assessment of the most effective survey questions is required. The questions used in the PMA surveys were the result of extensive pilot testing that aimed to appropriately capture the nuance of how women discuss and refer to abortion experiences.[88,89] Although the PMA pilot in Côte d'Ivoire suggested high understanding of the period regulation question[90], the higher interviewer effects for period regulation in both West African countries may have been caused by respondent confusion about the meaning of the question, resulting in the need for additional, unscripted clarification or explanation by the interviewers. Interviewer effects were similar for self-reporting and confidante-reporting, suggesting that this data quality issue is not reduced by using indirect reporting. Further cognitive interviewing and formative research could support identification of the most effective wording for asking about previous abortion experiences, and further work could also consider how 'removing a pregnancy' is understood compared to other commonly used terminologies. Use of familiar words is important for describing sensitive behaviours[91], but these are likely to vary considerably by context, creating challenges for cross-country comparisons. The finding that abortion reporting varied significantly based on the language of interview poses additional challenges for cross-national surveys, but also for countries where there are high levels of linguistic diversity. Understanding how the specific meanings and associations of question wording may vary between languages is also important during cognitive interviewing to assess potential impacts of specific wording on survey reporting.

The findings indicate that socio-demographic characteristics of interviewers were not significantly associated with respondents reporting a pregnancy removal, suggesting that the interview context or interviewer skills and behaviours may be more important than their observable characteristics. Previous studies of interviewer effects have often found the predictive power of variables from interviewer surveys are low, explaining only a small proportion of observed variance, but interviewer surveys can be strengthened through inclusion of questions relating to attitudes, behaviours, experiences and expectations of survey outcomes.[42] Use of interviewer observation or para-data on, for example, the speed of interview, may also provide further insight into the interviewer skills and behaviours that improve the validity of abortion reporting. Comfort asking questions about abortion was reportedly high but was significantly associated with abortion reporting at the 0.1 significance level in Côte d'Ivoire, suggesting additional values clarification training on abortion for interviewers may improve the quality of their data. Values clarification and attitude transformation workshops have been found to improve knowledge, attitudes and behavioural intentions relating to abortion and are intended to also reduce the impact of potential negative attitudes on professional responsibilities and ethics.[92]

In Rajasthan and Nigeria, respondent-interviewer familiarity was significantly associated with abortion reporting. Being slightly acquainted with an interviewer was negatively associated with abortion reporting compared to being well-acquainted, but there was no significant difference between reporting to a close acquaintance or a complete stranger. The historical norm of surveys has been to have strangers as interviewers, as respondents may wish to avoid judgement from a peer, or fear that their answers will not remain confidential. However, use of 'insiders' as interviewers may increase the interviewers' understanding of local culture, increase the likelihood of being invited into a private space, and can promote rapport, trust and closeness which can increase the motivation to answer truthfully.[48,63,93] The impact of interviewer-respondent familiarity will likely vary over time, and with the level of familiarity.[63] In these PMA surveys, it seems that a loose tie between the interviewer and respondent may not be conducive to abortion reporting, which may relate to concerns about confidentiality and trust, compared to a stranger or a close acquaintance. This finding may support future use of stranger interviewers, as it is difficult to systematically ensure that acquainted interviewers are closely acquainted, rather than slightly acquainted, with all respondents. However the results contrast with previous research that found limited difference in contraceptive reporting to local-insider and local-stranger interviewers in the Dominican Republic, while over-reporting was significantly higher to outsider-interviewers[64], suggesting that the benefits of personal and local familiarity may vary by context and topic.

Strengths and limitations

For peer review only

This analysis has a number of limitations. First, the most significant limitation of this study was that it was not possible to entirely separate interviewer effects and area effects, as the survey does not have an interpenetrated design where respondents or geographic areas are randomly assigned to interviewers. Cross-classified multilevel models have been found to effectively estimate interviewer effects without a randomised design[94], but in this study cross-classified modelling could not be used to separate area and interviewer effects because one interviewer is assigned to each cluster in PMA surveys. This is problematic as respondents in different clusters may have different probabilities of reporting abortions due to geographic, cultural or demographic differences, and interviewer effects cannot be separated from area effects. However, studies have found area random effects to be non-significant after controlling for household fixed effects in cross-classified models[95], so controlling for the wide range of respondent-level and community-level fixed effects included in this analysis (demographic and socio-economic characteristics of participants, region, urban/rural status, presence of abortion-providing facilities and number of abortions reported by these facilities) will account for area effects to some extent. Interviewer effects may be slightly over-estimated in this study since area effects may not be entirely explained by the respondent and community-level fixed effects. However, the only other analysis to have assessed interviewer effects for abortion reporting did use a cross-classified model to separate area and interviewer effects[41], and found interviewer effects were greater than area effects after controlling for region and rural/urban status, as done in this analysis.

Second, it is not possible to tell whether variables associated with higher abortion reports reflect an association with prior abortion experience or a willingness to report it. Though the inclusion of facility-reported data on number of abortions and abortion service availability was intended to partially account for this issue, there are likely inaccuracies in the facility-reports of abortion caseload. Third, interviewer survey data was missing for 4% of respondents in Côte d'Ivoire and 6% of respondents in Rajasthan. However, sensitivity analysis was conducted to compare the coefficients and standard errors for Model 1 when restricting the model to those cases that had complete interviewer survey data, and the results did not vary when comparing the model using all data to the model using only non-missing data. Fourth, for the sake of simplicity, the models do not include interactions between respondent and interviewer characteristics, which could be explored in future research. Fifth, while survey language was included as a potential explanatory variable in this analysis because linguistic differences in question wording was hypothesised to affect reporting due to potential differences in meanings and associations, the language spoken by respondents may reflect other respondent characteristics which are not fully accounted for in the model, such as religion and ethnicity (though region and education status are included). Finally, the limited variation in interviewer characteristics (Table 1) and the limited number of questions relating to interviewer attitudes, behaviours, experiences and expectations in the staff survey may have reduced the predictive power of the variables included in this analysis.

This study also has several strengths. It is the first to assess interviewer effects in the expanded abortion module used by the PMA surveys in 2018, and it is one of the first to assess interviewer effects for abortion questions in household surveys in LMICs. The analysis used data collected from diverse settings in the same time period using standardised questionnaires and trainings. The data provide a rich range of respondent and community characteristics, including abortion service delivery environment data, as well as interviewer characteristics (in two countries) and interview characteristics. Finally, the PMA model of using local interviewers enabled further exploration of the role of respondent-interviewer familiarity in the context of interviewer effects.

Conclusion

Surveys offer one of the only opportunities to gather representative evidence about the sources and safety of abortions, the sub-groups most affected by unsafe abortion and their abortion-seeking pathways. These data are critical to inform strategies, policies and programmes, and will become even more important with the shift towards self-managed medication abortions using drugs purchased informally through pharmacies

For peer review only

or online. Understanding interviewer effects for abortion reporting can help inform decisions about whether to include abortion questions in demographic and public health surveys, which questions to use, and how abortion survey data quality can be improved through methodological adjustments. This analysis highlights that interviewer effects for abortion reporting were high in the PMA surveys in Côte d'Ivoire, Nigeria and Rajasthan from 2018. Further work is needed to identify the effect of different question wordings on abortion reporting through comparative studies and more comprehensive qualitative and cognitive interviewing. Observable interviewer characteristics were not significantly associated with abortion reporting, suggesting skills and behaviours of interviewers may be responsible for unexplained variance at the interviewer level, and this could be further explored through the addition of relevant variables to the interviewer survey. Additional values clarification trainings or other mechanisms to address issues of abortion stigma and ensure interviewers feel comfortable asking questions about abortion may improve the quality of abortion data from surveys. Interview characteristics, including respondent-interviewer familiarity and language of interview, were also significantly associated with abortion reporting, which raises challenges for survey logistics when using 'insider' interviewers, for countries with high linguistic diversity and cross-national surveys. Consideration of variations in language should inform testing of different question wordings and designs in future work.

Funding: The UK Economic and Social Research Council funded this analysis as part of a 1+3 doctoral grant (F79676A). The PMA abortion module was funded by an Anonymous Donor. The funders had no role in study design, data collection and analysis, decision to publish, or preparation of the manuscript.

Acknowledgements: I am extremely grateful to Dr Suzanne Bell for enabling my access to the PMA interviewer data, for her feedback on the analysis and paper, and for her support throughout the project. This study was completed as part of a Masters dissertation, supervised by Professor Patrick Sturgis, and I am very grateful for his advice and guidance. I would also like to thank my PhD supervisors, Professor Ernestina Coast and Dr Tiziana Leone, for their reviews of this paper, and Dr Laura Sochas for very helpful discussions at the analysis stage. I am grateful to all the researchers involved in the design, management and sharing of PMA survey data at Johns Hopkins Bloomberg School of Public Health, the Centre for Research, Evaluation Resources and Development (CRERD), the Écoles Nationale de Statistiques et d'Economie Appliquee (ENSEA) and the Indian Institute of Health Management Research. Finally, I would like to gratefully acknowledge the time and contributions made by the participants and interviewers of each of the surveys.

Contributorship statement: This analysis was conceptualised and conducted and the manuscript was written by the sole author (KF).

Data sharing statement: PMA survey datasets can be requested through the PMA website: https://www.pmadata.org/data/available-datasets/request-access-datasets.

Competing interests: There are no competing interests.

List of figures:

Figure 1: Caterpillar plots showing interviewer effects (Level 2 Residuals) with 95% confidence intervals for the log-odds of reporting ever removing a pregnancy, adjusted for respondent and community characteristics

Figure 2: Intra-interviewer correlation by country and survey question, with 95% confidence intervals, adjusted for respondent and community characteristics (Model 1)

Bibliography

1. Singh S, Remez L, Sedgh G, Kwok L, Tsuyoshi O. *Abortion Worldwide 2017: Uneven Progress and Unequal Access*. New York; 2018.

For peer review only

2. Ganatra B, Gerdts C, Rossier C, et al. Global, regional, and subregional classification of abortions by safety, 2010–14: estimates from a Bayesian hierarchical model. *Lancet*. 2017;390(10110):2372-2381.
3. WHO. *Safe Abortion: Technical and Policy Guidance for Health Systems*. Geneva; 2012.
4. Sedgh G, Keogh SC. Novel approaches to estimating abortion incidence. *Reprod Health*. 2019;16(1):1-10.
5. Thapa S. Abortion law in Nepal: The road to reform. *Reprod Health Matters*. 2004;12(24):85-94.
6. Chavkin W, Stifani BM, Bridgman-Packer D, Greenberg JMS, Favier M. Implementing and expanding safe abortion care: An international comparative case study of six countries. *Int J Gynecol Obstet*. 2018;143:3-11.
7. Bell S. Abortion Reporting and Underreporting: Can Better Design Yield Better Data? Doctoral Dissertation, Johns Hopkins University. 2018. http://jhir.library.jhu.edu/handle/1774.2/59185.
8. Harvey P. Medical abortion: the hidden revolution. *J Fam Plann Reprod Heal Care*. 2015;41(3):193-196.
9. Bullough C, Meda N, Makowiecka K, Ronsmans C, Achadi EL, Hussein J. Current strategies for the reduction of maternal mortality. *BJOG An Int J Obstet Gynaecol*. 2005;112(9):1180-1188.
10. Chae S, Desai S, Crowell M, Sedgh G, Singh S. Characteristics of women obtaining induced abortions in selected low- and middle-income countries. *PLoS One*. 2017;12(3):1-19.
11. Bell SO, Shankar M, Omoluabi E, et al. Social network-based measurement of abortion incidence: promising findings from population-based surveys in Nigeria, Cote d'Ivoire, and Rajasthan, India. *Popul Health Metr*. 2020;18(1):1-15. doi:10.1186/s12963-020-00235-y
12. Randall S, Coast E, Compaore N, Antoine P. The power of the interviewer. *Demogr Res*. 2013;28:763-792.
13. Bell SO, Bishai D. The impact of respondent–interviewer familiarity and repeated survey participation on abortion reporting: Evidence from rajasthan, india. *Int Perspect Sex Reprod Health*. 2019;44(4):147-156.
14. Safi S, Greenleaf A, Hawes M, et al. Resident Interviewers and Repeat Surveys: Effects on Measures of Reproductive Health. In: *Population Associations of America Conference*. Chicago; 2017.
15. MacQuarrie KLD, Winfrey W, Meijer-Irons J, Morse AR. Consistency of reporting of terminated pregnancies in DHS calendars . *DHS Methodol Reports No 25* . 2018;(September). http://dhsprogram.com/pubs/pdf/MR25/MR25.pdf.
16. Rossier C. Estimating induced abortion rates: A review. *Stud Fam Plann*. 2003;34(2):87-102.
17. Anderson BA, Katus K, Puur A, Silver BD. The Validity of Survey Responses on Abortion : Evidence from Estonia. *Demography*. 1994;31(1):115-132.
18. Edmeades J, Nyblade L, Malhotra A, MacQuarrie K, Parasuraman S, Walia S. Methodological innovation in studying abortion in developing countries: A "narrative" quantitative survey in Madhya Pradesh, india. *J Mix Methods Res*. 2010;4(3):176-198.
19. Udry JR, Gaughan M, Schwingl PJ, Berg BJ Van Den. A Medical Record Linkage Analysis of Abortion Underreporting. *Fam Plann Perspect*. 1996;28(5):228-231.
20. Huntington D, Mensch B, Miller VC. Survey Questions for the Measurement of Induced Abortion. *Stud Fam Plann*. 1996;27(3):155.
21. Scott R, Bajos N, Wellings K, Slaymaker E. Comparing reporting of abortions in three nationally-representative surveys: Methodological and contextual influences. *BMJ Sex Reprod Heal*. 2019;45(3):213-219.
22. Moreau C, Bajos N, Bouyer J. Question comprehension and recall: the reporting of induced abortions in quantitative surveys on the general population. *Population (Paris)*. 2004;59(3):439-454.
23. Bell S, Shankar M, Guiella G, et al. P33 Menstrual regulation: Incidence, methods, and sources of this understudied reproductive practice in three countries. *Contraception*. 2020;102(4):287.
24. Lindberg L, Scott RH. Effect of ACASI on Reporting of Abortion and Other Pregnancy Outcomes in the US National Survey of Family Growth. *Stud Fam Plann*. 2018;49(3):259-278.

For peer review only

25. Lara D, García SG, Ellertson C, Camlin C, Suárez J. The measure of induced abortion levels in Mexico using random response technique. *Sociol Methods Res*. 2006;35(2):279-301.

26. Lara D, Strickler J, Olavarrieta CD, Ellertson C. Measuring induced abortion in Mexico: a comparison of four methodologies. *Sociol Methods Res*. 2004;32(4):529-558.

27. Singh S, Remez L, Tartaglione A. *Methodologies for Estimating Abortion Incidence and Abortion-Related Morbidity: A Review*. New York and Paris; 2010.

28. Moseson HS, Gerdts C, Fuentes L, et al. Measuring Texas women's experiences with abortion self-induction using a list experiment. *Contraception*. 2017;96(4):272.

29. Moseson H, Massaquoi M, Dehlendorf C, et al. Reducing under-reporting of stigmatized health events using the List Experiment: Results from a randomized, population-based study of abortion in Liberia. *Int J Epidemiol*. 2015;44(6):1951-1958.

30. Cowan SK, Wu LL, Makela S, England P. Alternative estimates of lifetime prevalence of abortion from indirect survey questioning methods. *Perspect Sex Reprod Health*. 2016;48(4):229-234.

31. Elewonibi B, Gleason S, Msuya S, Shah I, Mahande M, Canning D. Estimating Prevalence of Abortion in Tanzania via Direct Question and the List Experiment. In: *APHA's 2019 Annual Meeting and Expo (Nov. 2-Nov. 6)*. American Public Health Association; 2019.

32. Bell SO, Bishai D. Can a List Experiment Improve Validity of Abortion Measurement? *Stud Fam Plann*. 2019;50(1):43-61.

33. Keogh SC, Otupiri E, Chiu DW, et al. Estimating the incidence of abortion: a comparison of five approaches in Ghana. *BMJ Glob Heal*. 2020;5(4):e002129.

34. Rossier C. Measuring abortion with the Anonymous Third Party Reporting Method. In: Singh S, Remez L, Tartiglione A, eds. *Methodologies for Estimating Abortion Incidence and Abortion-Related Morbidity: A Review.* New York and Paris: Guttmacher Institute and IUSSP; 2010:99-106. http://www.guttmacher.org/pubs/compilations/IUSSP/IUSSP-Chapter7.pdf.

35. Elul B. Anonymous third party reporting of induced abortion: An experiment in Rajasthan, India. In: *Annual Meeting of the Population Association of America*. Boston; 2004.

36. Yeatman S, Trinitapoli J. Best-friend reports: A tool for measuring the prevalence of sensitive behaviors. *Am J Public Health*. 2011;101(9):1666-1667.

37. Tourangeau R, Yan T. Sensitive Questions in Surveys. *Psychol Bull*. 2007;133(5):859-883.

38. Jones EF, Forrest JD. Underreporting of abortion in surveys of U.S. women: 1976 to 1988. *Demography*. 1992;29(1):113-126.

39. Jagannathan R. Relying on surveys to understand abortion. *Am J Public Health*. 2001;91(11):1825-1831.

40. Broussard K, Hathi P, Coffey D. Assessing public awareness and use of medical abortion via mobile phone survey in India. *Contraception*. 2019;100(6):457-463.

41. Leone T, Sochas L, Coast E. Depends who's asking: interviewer effects in Demographic and Health Surveys abortion data. *Demography*. 2020;In press.

42. Blom AG, Julie M. Korbmacher. Measuring Interviewer Characteristics Pertinent to Social Surveys: A Conceptual Framework. *Surv Methods Insights from F*. 2013:1-16.

43. GSS, GHS, ICF. *Ghana Maternal Health Survey 2017.* Accra, Ghana; 2018.

44. IIPS, ICF. *National Family Health Survey (NFHS-4), 2015-16: India*. Mumbai; 2017.

45. Bell SO, OlaOlorun F, Shankar M, et al. Measurement of abortion safety using community-based surveys: Findings from three countries. *PLoS One*. 2019;14(11):1-14.

46. Davis RE, Couper MP, Janz NK, Caldwell CH, Resnicow K. Interviewer effects in public health surveys. *Health Educ Res*. 2010;25(1):14-26.

47. Rodriguez LA, Sana M, Sisk B. Self-administered Questions and Interviewer–Respondent Familiarity. *Field methods.* 2015;27(2):163-181. doi:10.1177/1525822X14549315

48. Weinreb A, Sana M, Stecklov G. Strangers in the Field: A Methodological Experiment on Interviewer–Respondent Familiarity. *BMS Bull Sociol Methodol*. 2018;137-138(1):94-119.

49. West BT, Blom AG. Explaining interviewer effects: A research synthesis. *J Surv Stat Methodol*. 2017;5(2):175-211.

For peer review only - http://bmjopen.bmj.com/site/about/guidelines.xhtml

50. Schnell R, Kreuter F. Separating interviewer and sampling-point effects. *J Off Stat*. 2005;21(3):389-410.

51. Pickery J, Loosveldt G. An Exploration of Question Characteristics that Mediate Interviewer Effects on Item Nonresponse. *J Off Stat*. 2001;17(3):337-350.

52. Schaeffer NC, Dykema J, Maynard D. Interviewers and Interviewing. In: Marsden P, Wright J, eds. *Handbook of Survey Research*. 2nd ed. Bingley: Emerald Group Publishing Limited; 2014:437-471.

53. West BT, Conrad FG, Kreuter F, Mittereder F. Nonresponse and measurement error variance among interviewers in standardized and conversational interviewing. *J Surv Stat Methodol*. 2018;6(3):335-359.

54. West BT, Olson K. How much of interviewer variance is really nonresponse error variance? *Public Opin Q*. 2010;74(5):1004-1026. doi:10.1093/poq/nfq061

55. Groves RM, Magilavy LJ. Measuring and explaining interviewer effects in centralized telephone surveys. *Public Opin Q*. 1986;50(2):251-266.

56. Hox JJ. Hierarchical Regression Models for Interviewer and Respondent Effects. *Sociol Methods Res*. 1994;22(3):300-318.

57. Amos M. Interviewer effects on patterns of nonresponse: Evaluating the impact on the reasons for contraceptive nonuse in the Indonesia and the Philippines DHS. *Demogr Res*. 2018;39(1):415-430.

58. Assche SB Van, Reniers G, Weinreb AA. An assessment of the KDICP and MDICP data quality: Interviewer effects, question reliability and sample attrition. *Demogr Res*. 2003;9(1):31-76.

59. Agula J, Barrett JB, Tobi H. The other side of rapport: Data collection mode and interviewer gender effects on sexual health reporting in Ghana. *Afr J Reprod Health*. 2015;19(3):111-117.

60. Becker S, Feyisetan K, Makinwa-Adebusoye P. The Effect of the Sex of Interviewers on the Quality of Data in a Nigerian Family Planning Questionnaire. *Stud Fam Plann*. 1995;26(4):233.

61. Johnson K, Grant M, Khan S, Moore Z, Armstrong A, Sa Z. *Fieldwork-Related Factors and Data Quality in the Demographic and Health Surveys Program.* Calverton; 2009.

62. McCombie SC, Anarfi JK. The influence of sex of interviewer on the results of an AIDS survey in Ghana. *Hum Organ*. 2002;61(1):51-57.

63. Anglewicz P, Akilimali P, Eitmann LP, Hernandez J, Kayembe P. The relationship between interviewer-respondent familiarity and family planning outcomes in the Democratic Republic of Congo: A repeat cross-sectional analysis. *BMJ Open*. 2019;9(1):1-9.

64. Stecklov G, Weinreb AA, Sana M, Anglewicz P. Family planning for strangers: An experiment on the validity of reported contraceptive use. *PLoS One*. 2015;10(8):1-19.

65. Weinreb A. The Limitations of Stranger-Interviewers in Rural Kenya. *Am Sociol Rev*. 2006;71:1014-1039.

66. [dataset] Centre for Population and Reproductive Health (CPRH) University of Ibadan, Centre for Research, Evaluation Resources and Development (CRERD), Population and Reproductive Health Program (PRHP), Obafemi Awolowo University (OAU), Bayero University Kano (BUK), The Bill & Melinda Gates Institute for Population and Reproductive Health at The Johns Hopkins Bloomberg School of Public Health. Performance Monitoring and Accountability 2020 (PMA2020) Household and Female Survey Round 5, PMA2017/Nigeria-R5-HQFQ. 2018. doi:https://doi.org/10.34976/b5eg-a867

67. [dataset] Centre for Population and Reproductive Health (CPRH) University of Ibadan, Centre for Research, Evaluation Resources and Development (CRERD), Population and Reproductive Health Program (PRHP), Obafemi Awolowo University (OAU), Bayero University Kano (BUK), The Bill & Melinda Gates Institute for Population and Reproductive Health at The Johns Hopkins Bloomberg School of Public Health. Performance Monitoring and Accountability 2020 (PMA2020) Service Delivery Point Survey Round 5, PMA2017/Nigeria-R5-SQ. 2018. doi:https://doi.org/10.34976/xjzj-y288

68. [dataset] Institut National de la Statistique de la Côte d'Ivoire (INS-Côte d'Ivoire), La Direction de Coordination du Programme National de Santé de la Mère et de l'Enfant (DC-PNSME), The Bill & Melinda Gates Institute for Population and Reproductive Health at The Johns Hopkins Bloomberg School of Public Health. Performance Monitoring and Accountability 2020 (PMA2020) Household and Female Survey Round 2, PMA2018/Côte d'Ivoire-R2-HQFQ. 2018.

For peer review only

doi:https://doi.org/10.34976/716t-1697

69. [dataset] Institut National de la Statistique de la Côte d'Ivoire (INS-Côte d'Ivoire), La Direction de Coordination du Programme National de Santé de la Mère et de l'Enfant (DC-PNSME), The Bill & Melinda Gates Institute for Population and Reproductive Health at The Johns Hopkins Bloomberg School of Public Health. Performance Monitoring and Accountability 2020 (PMA2020) Service Delivery Point Survey Round 2, PMA2018/Côte d'Ivoire-R2-SQ. 2018. doi:https://doi.org/10.34976/nd0a-ew46

70. [dataset] Indian Institute of Health Management Research (IIHMR) University in Jaipur, The Bill & Melinda Gates Institute for Population and Reproductive Health at The Johns Hopkins Bloomberg School of Public Health. Performance Monitoring and Accountability 2020 (PMA2020) Household and Female Survey Round 4, PMA2018/India-R4-HQFQ. 2018. doi:https://doi.org/10.34976/87gc-wn69

71. [dataset] Indian Institute of Health Management Research (IIHMR) University in Jaipur, The Bill & Melinda Gates Institute for Population and Reproductive Health at The Johns Hopkins Bloomberg School of Public Health. Performance Monitoring and Accountability 2020 (PMA2020) Service Delivery Point Survey Round 4, PMA2018/India-R4-SQ. 2018. doi:https://doi.org/10.34976/tnkm-fk45

72. Shellenberg KM, Moore AM, Bankole A, et al. Social stigma and disclosure about induced abortion: Results from an exploratory study. *Glob Public Health*. 2011;6(1):111-125.

73. Singh S, Shekhar C, Acharya R, et al. The incidence of abortion and unintended pregnancy in India, 2015. *Lancet Glob Heal*. 2018;6(1):e111-e120.

74. Akiode A, Fetters T, Okoh M, et al. The availability of misoprostol in pharmacies and patent medicine stores in two Nigerian cities. *Ebonyi Med J*. 2010;9(2).

75. Stillman M, Owolabi O, Fatusi AO, et al. Women's self-reported experiences using misoprostol obtained from drug sellers: a prospective cohort study in Lagos State, Nigeria. *BMJ Open*. 2020;10(5):e034670.

76. PMA2020. *PMA2020 Abortion Survey Results: Cote D'Ivoire*.; 2018. https://www.pma2020.org/sites/default/files/Cote d'Ivoire_AbortionModule_Brief_EN_012019.pdf.

77. PMA2020. *PMA2020 Abortion Survey Results: Nigeria*.; 2018. https://www.pma2020.org/sites/default/files/AbortionModule_Brief_111518.pdf.

78. Guillaume A, Du Loû AD. Fertility regulation among women in Abidjan, Côte d'Ivoire: contraception, abortion or both? *Int Fam Plan Perspect*. 2002;28(3):159-166.

79. Zimmerman L, Olson H, Group PPI, Tsui A, Radloff S. PMA2020: Rapid turn-around survey data to monitor family planning service and practice in ten countries. *Stud Fam Plann*. 2017;48(3):293-303.

80. Plummer ML, Wamoyi J, Nyalali K, et al. Aborting and Suspending Pregnancy in Rural Tanzania: An Ethnography of Young People's Beliefs and Practices. *Stud Fam Plann*. 2008;39(4):281-292.

81. Ladipo OA, Ojo OA, James S, Stewart KR. Menstrual regulation in Ibadan, Nigeria. *Int J Gynecol Obstet*. 1978;15(5):428-432.

82. Jones RK, Kost K. Underreporting of induced and spontaneous abortion in the United States: An analysis of the 2002 National Survey of Family Growth. *Stud Fam Plann*. 2007;38(3):187-197.

83. Steele F. Multilevel Models for Binary Responses. LEMMA VLE Module 7.

84. Stuart GS, Grimes DA. Social desirability bias in family planning studies: a neglected problem. *Contraception*. 2009;80(2):108-112.

85. Smith C, Edwards P, Free C. Assessing the validity and reliability of self-report data on contraception use in the MObile Technology for Improved Family Planning (MOTIF) randomised controlled trial. *Reprod Health*. 2018;15(1):50.

86. Nwaohiri AN, Tang JH, Stanczyk F, et al. Discordance between self-reported contraceptive use and detection of exogenous hormones among Malawian women enrolling in a randomized clinical trial. *Contraception*. 2018;97(4):354-356.

87. Biddlecom AE, Fapohunda BM. Covert Contraceptive Use: Prevalence, Motivations, and Consequences. *Stud Fam Plann*. 1998;29(4):360-372. doi:10.2307/172249

88. Bell SO, Omoluabi E, OlaOlorun F, Shankar M, Moreau C. Inequities in the incidence and safety of abortion in Nigeria. *BMJ Glob Heal*. 2020;5(1).

89. Bell S, Shankar M, Omoluabi E, et al. Methodological Advances in Survey-Based Abortion Estimation: Promising Findings from Nigeria, India, and Cote d'Ivoire. In: *Population Association of America Annual Meeting*. Austin, Texas; 2019.

90. Bell SO, Sheehy G, Hyacinthe AK, Guiella G, Moreau C. Induced abortion incidence and safety in Côte d'Ivoire. *PLoS One*. 2020;15(5):e0232364.

91. Groves RM, Fowler FJ, Couper MP, Lepkowski JM, Singer E, Tourangeau R. *Survey Methodology*. 2nd ed. Wiley; 2009.

92. Turner KL, Pearson E, George A, Andersen KL. Values clarification workshops to improve abortion knowledge, attitudes and intentions: a pre-post assessment in 12 countries. *Reprod Health*. 2018;15(1):40.

93. Kühne S. From strangers to acquaintances? Interviewer continuity and socially desirable responses in panel surveys. *Surv Res Methods*. 2018;12(2):121-146.

94. Brunton-Smith I, Sturgis P, Leckie G. Detecting and understanding interviewer effects on survey data by using a cross-classified mixed effects location–scale model. *J R Stat Soc*. 2017;180(2):551-568.

95. Vassallo R, Durrant G, Smith P. Separating interviewer and area effects by using a cross-classified multilevel logistic model: simulation findings and implications for survey designs. *J R Stat Soc*. 2017;180(2):531-550.

For peer review only

Table 1. Characteristics of interviewers and interview (weighted), by country

	N	%	N	%	N	%^
**Interviewer characteristics**	Côte d'Ivoire (n=70)		Rajasthan, India (n=145)		Nigeria (n=285)	
Mean respondents per interviewer	41		43		43	
**Socio-demographics**						
Mean age (SE)	31.1 (0.63)		26.9 (0.55)			
Female	70	100%	134	100%		
Married	14	20%	107	80%		
Has children	43	61%	86	64%		
**Education**						
Primary or secondary	10	14%	65	49%		
Technical or graduate	60	86%	69	51%		
**Experience**						
Existing staff	68	97%	111	83%		
Previous survey experience	63	90%	26	19%		
Comfortable asking about abortion	66	94%	130	97%		
Existing staff	68	97%	111	83%		
**Interview characteristics**	Côte d'Ivoire (n=2798)		Rajasthan, India (n=5915)		Nigeria (n=11,303)	
**Interviewer-respondent familiarity**						
Very or well acquainted	42	1%	3,286	53%	1,783	19%
Not well acquainted	109	3%	1,893	33%	3,654	32%
Not at all acquainted	2,654	95%	741	14%	5,915	49%
**Language of interview**						
French ¦ Hindi ¦ Hausa	1,977	71%	5,673	94%	5,494	48%
Baoule ¦ English ¦ English	170	6%	44	2%	4,350	37%
Yacouba ¦ Other ¦ Igbo	69	3%	198	4%	850	8%
Attie ¦ - ¦ Yoruba	29	1%			159	2%
Dioula ¦ - ¦ Pidgin	438	14%			247	3%
Lobi ¦ - ¦ Other	54	3%			203	3%
Other ¦ - ¦ -	2%	2%				

Abbreviations: SE = Standard Error. Note: Interviewer survey data was not collected in Nigeria (n=285). Interviewer survey data were missing for 3 interviewers (123 respondents) in Côte D'Ivoire and 11 interviewers (384 respondents) in Rajasthan.

Table 2. Reported abortions of respondents and their closest confidante by country, weighted

	Côte d'Ivoire			Rajasthan, India			Nigeria		
	N	%	95% CI	N	%	95% CI	N	%	95% CI
**Self-reported**									
Pregnancy removal	511	19	[16;22]	390	7	[6;8]	1,392	15	[13;16]
Period regulation	222	7	[5;10]	109	2	[1;2]	679	7	[6;8]
**Closest confidante**									
Pregnancy removal	305	18	[14;21]	705	15	[12;17]	1,120	20	[18;22]
Period regulation	161	8	[6;22]	294	6	[4;8]	556	9	[8;11]
Total n respondents	2,795			5,912			11,254		
Total n confidantes	1,803			4,983			5,986		

For peer review only

Notes: For self-reported pregnancy removal a small number of respondents were coded as -99 (no response) in each country: 3 (0.1%) in Côte D'Ivoire, 3 (0.04%) in Rajasthan, 49 (0.4%) in Nigeria. For self-reported period regulation, the number of non-responses were 2, 9 and 47 respectively.
A high proportion of respondents reported that they did not have any close female confidantes age 15-49 who they mutually shared very personal information with: 991 (35.5%) in Côte D'Ivoire, 893 (15.2%) in Rajasthan and 4984 (45.4%) in Nigeria, resulting in a smaller sample size for these questions.

For peer review only

Table 3: Full multilevel random intercept logit model (Model 2) for the odds of reporting removing a pregnancy, adjusted for interviewer, interview, respondent and community characteristics*

	Côte d'Ivoire		Rajasthan, India		Nigeria	
	OR	[95% CI]	OR	[95% CI]	OR	[95% CI]
**Interviewer characteristics**						
Number of respondents	1.00	[0.98;1.01]	0.99	[0.97;1.01]	**0.99**	**[0.98;1.00]**
Interviewer age	0.99	[0.96;1.02]	1.02	[0.99;1.06]		
Married (vs not married)	1.17	[0.84;1.64]	0.73	[0.39;1.34]		
Has children (vs no children)	***1.33***	***[0.96;1.83]***	1.52	[0.85;2.74]		
Technical / Uni grad (vs secondary)	***1.47***	***[0.95;2.29]***	1.08	[0.73;1.59]		
Existing staff (vs new staff)	1.34	[0.52;3.44]	1.11	[0.66;1.87]		
Previous survey experience (v none)	1.36	[0.82;2.26]	1.01	[0.62;1.64]		
Very comfortable asking about abortion (vs somewhat/not comfortable)	***1.88***	***[0.9;3.9]***	1.55	[0.45;5.34]		
**Interview Characteristics**						
Very well or well acquainted	Ref		Ref		Ref	
Not well acquainted	1.22	[0.28;5.22]	**0.68**	**[0.49;0.96]**	**0.75**	**[0.58;0.97]**
Not acquainted	1.67	[0.45;6.17]	0.91	[0.57;1.45]	0.85	[0.65;1.13]
Language of interview (Côte d'Ivoire / India / Nigeria)						
French / Hindi / Hausa	Ref		Ref		Ref	
Baoule / English / English	**3.11**	**[2.00;4.83]**	0.77	[0.08;6.97]	**1.59**	**[1.19;2.14]**
Yacouba / Other / Igbo	0.54	[0.15;1.93]	1.54	[0.69;3.47]	1.13	[0.72;1.78]
Attie / - / Yoruba	0.75	[0.28;2.02]			**1.94**	**[1.16;3.25]**
Dioula / - / Pidgin	**0.26**	**[0.14;0.48]**			**1.57**	**[0.99;2.47]**
Lobi / - / Other	***0.17***	***[0.02;1.35]***			1.61	[0.73;3.55]
Other / - / -	0.67	[0.27;1.72]				
**Intra-Interviewer Correlation**	**0.01**		**0.15**		**0.22**	

Notes: Coefficients in bold are significant at the <0.05 level, coefficients in bold and italics are significant at the <0.10 level. Interviewer characteristics were unavailable for Nigeria. OR = odds ratio. CI = confidence intervals.
*Respondent characteristics include age, age squared, education, marital status, ever given birth and number of birth events, wealth quintile and whether the respondent is a previous PMS respondent. Community characteristics include region or state, rural/urban status, monthly number of abortions per community (mean, facility-reported) and whether there is an abortion care facility in the community. The full model is available in Appendix 3, including all respondent and community variable coefficients.

For peer review only

For peer review only

For peer review only - http://bmjopen.bmj.com/site/about/guidelines.xhtml

Figure 1. Caterpillar plots showing interviewer effects (Level 2 Residuals) with 95% confidence intervals for the log-odds of reporting ever removing a pregnancy, adjusted for respondent and community characteristics

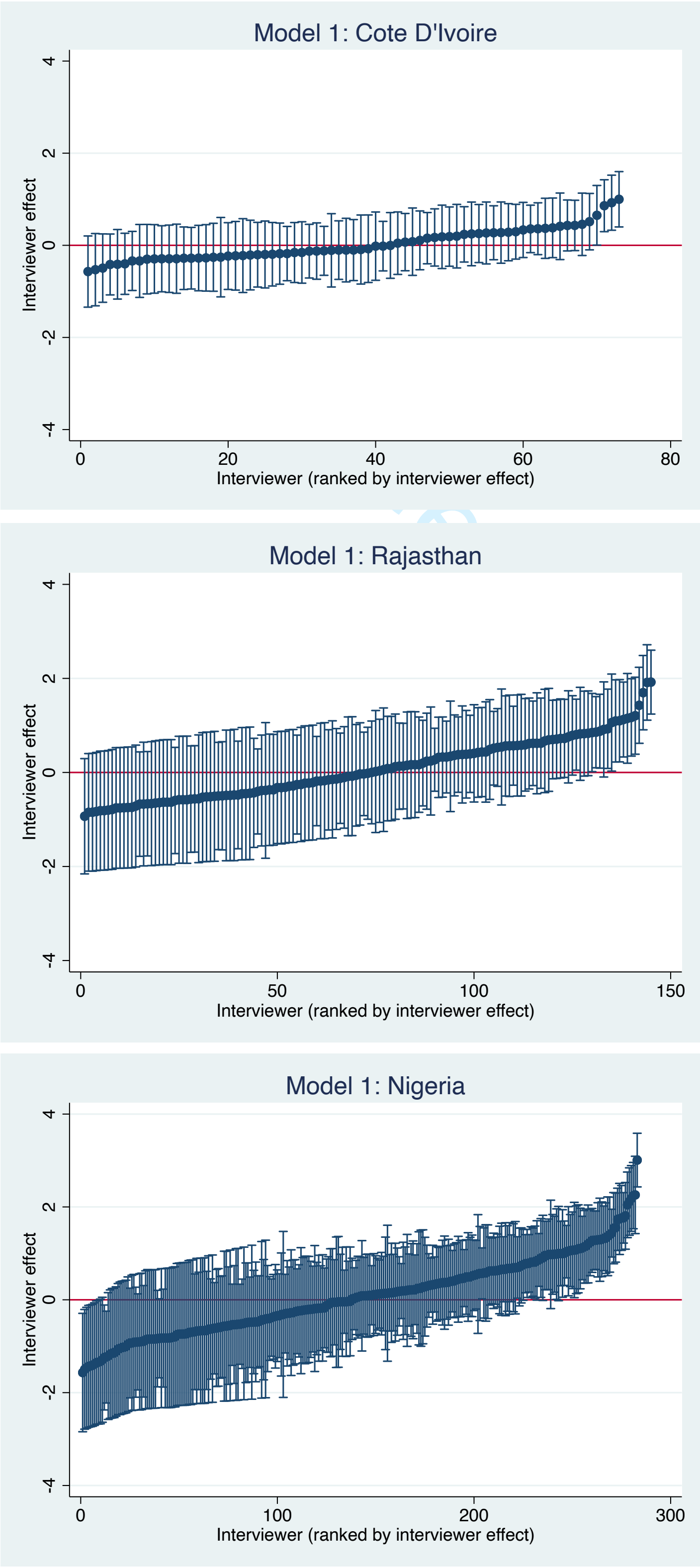

Figure 2. Intra-interviewer correlation by country and survey question, with 95% confidence intervals, adjusted for respondent and community characteristics (Model 1)

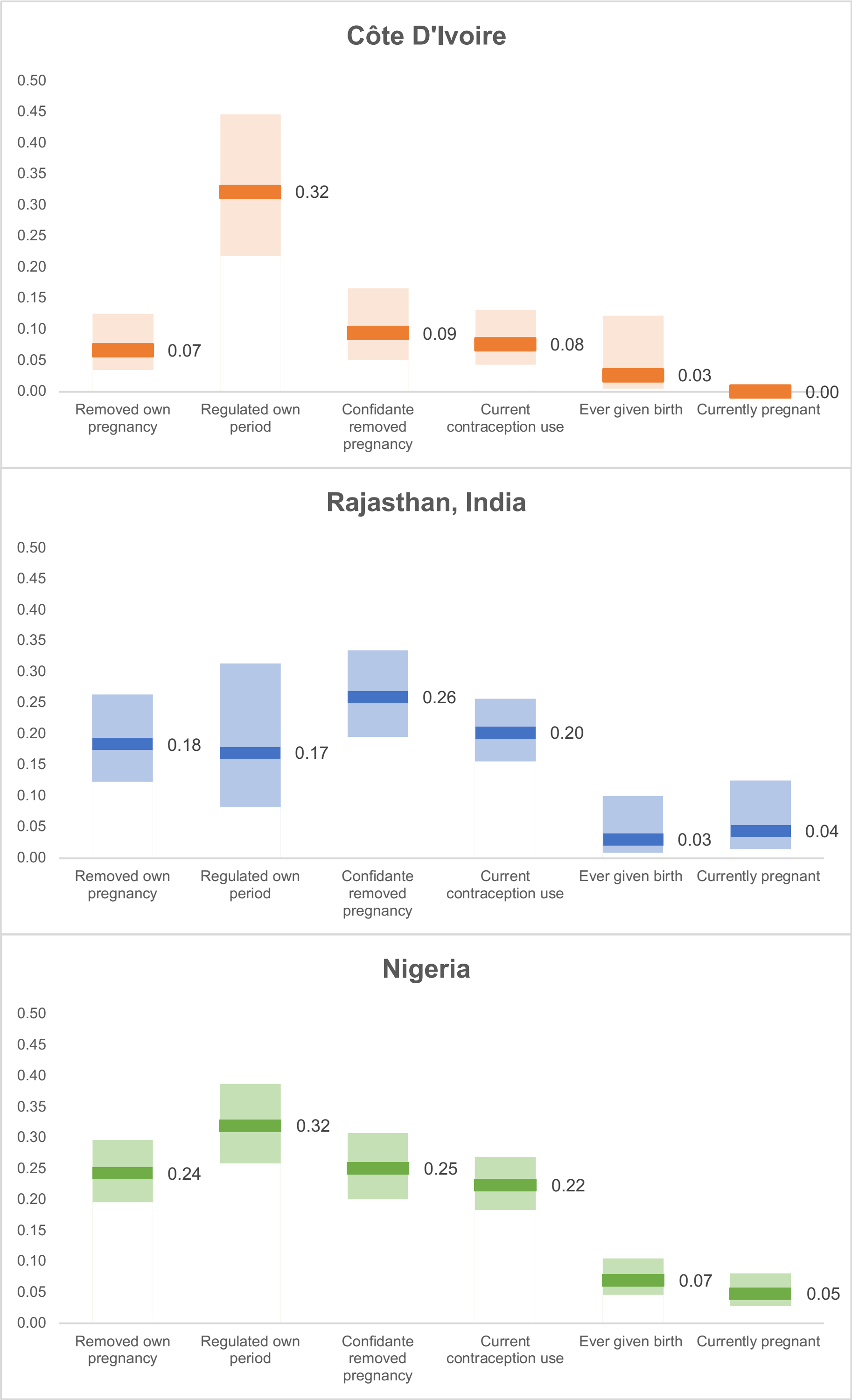

For peer review only

Appendix 1. Details of explanatory variables

Variable	Question	Categories	Re-code
**Respondent-Level Variables**			
**Age**	How old were you at your last birthday? [confirmed agreement with birth date]	Integer	- Treated as continuous
**Education**	What is the highest level of school you attended? [Only record formal schooling]	Never attended<br>Primary<br>Secondary<br>Higher Secondary [India only]<br>Tertiary / Higher / Graduate<br>No response	- 'Higher secondary' grouped with 'Secondary'<br>- No response treated as missing
**Marital status**	Are you currently married or living together with a man as if married? [Probe: If no, ask whether the respondent is divorced, separated, or widowed.]	Currently married<br>Currently married, gauna not performed [India only]<br>Living with a man<br>Divorced / separated<br>Widow<br>No, never in union<br>No response	- 'Married, gauna not performed' coded as 'Currently married'<br>- Divorced/separated grouped with Widowed<br>- No response treated as missing.
**Ever given birth**	Have you ever given birth?	Yes<br>No<br>No response	- No response treated as missing.
**Number of birth events**	How many times have you given birth?	Integer	- No response treated as missing
**Wealth**	Household wealth index questions administered in household questionnaire	Lowest quintile<br>Lower quintile<br>Middle quintile<br>Higher quintile<br>Highest quintile	- Pre-constructed in dataset
**Previous PMA participation**	Has the respondent previously participated in PMA 2020 surveys? [completed by interviewer]	Yes<br>No<br>Do not know<br>No response	- Do not know and No response coded as missing
**Community-Level Variables**			
**Region / State**	Enumeration area categorised based on sampling frame	Vary by country	- Pre-constructed in dataset
**Urban /rural**		Urban<br>Rural	- Pre-constructed in dataset
**Abortion care facility in community**	Does this facility provide post-abortion care for pregnancies of 12 weeks or less / more than 12 weeks?<br>AND<br>Does this facility provide abortion for pregnancies of 12 weeks or less / more than 12 weeks?	Yes<br>No<br>Do not know<br>No response	- Binary variable coded 1 if any facilities in or serving the enumeration area answered yes to providing abortion or post-abortion care for either gestational age group, and 0 if all facilities answered no to all questions. Public hospitals were excluded as hospital catchment areas can be very large, so an enumeration area may not have abortion care available if it only has a public hospital serving the area.
**Monthly mean number of abortions**	In an AVERAGE / the LAST month, how many post-abortion care patients would you estimate are/were treated as INPATIENTS / OUTPATIENTS in this facility as a whole? Please remember to include all patients	Integer variable<br>-88 for do not know<br>-99 for no response.	- Average of the number of post-abortion care services in the last month and in an average month was calculated for each facility (to get a more accurate average that accounts for possible seasonal variation).<br>- Average of the number of abortion services in the last month and in an average month was calculated for each facility (to get a

For peer review only

	treated for extremely serious or minor abortion complications, whether the abortions were spontaneous or induced. Ensure provider responds from memory and does NOT reference the log book.<br><br>In an AVERAGE / the LAST month, how many abortions are/were provided?		-	more accurate average that accounts for possible seasonal variation).<br>The average numbers of abortions and post-abortion care services were summed per facility, and then per cluster to give a cluster total of the number of abortions / post-abortion care services reported by facilities per month on average.
**Interview-level Variables**				
**Interviewer-respondent familiarity**	How well acquainted are you with the respondent? [completed by interviewer]	Very well acquainted<br>Well acquainted<br>Not well acquainted<br>Not acquainted	-	Very well acquainted and well acquainted grouped together to give a categorical variable with three groups.
**Survey language**	Administrative data recorded by interviewer	Varied by country	-	Languages used in <20 interviews grouped into 'Other'
**Interviewer-level Variables**				
**Number of respondents**	-	-	-	Constructed by summing the number of interviews completed per interviewer
**Age**	How old were you at your last birthday?	Integer	-	Treated as continuous
**Marital status**	Select your marital status	Never Married<br>Currently Married<br>Living with a partner<br>Divorced<br>Widowed	-	Coded as binary variable where 1 is married ('Currently Married') and 0 is all other categories
**Children**	Do you have any children?	Yes<br>No	-	
**Education**	What is the highest level of education that you have completed?	Primary School<br>Secondary School<br>Higher Secondary [India only]<br>University<br>Technical School<br>Graduate School (Masters)<br>Graduate School (Doctoral)<br>[Côte D'Ivoire only]	-	Recoded into a binary variable where 1 is "Primary/Secondary" and 2 is "Technical / Uni graduate and above"
**Existing staff**	Have you ever completed a round of data collection for PMA2020 before the round you are preparing for now?	Yes, I am a returning PMA2020 staff member<br>No, this will be my first round of data collection	-	
**Previous experience**	Do you have any previous survey experience?	Yes<br>No	-	
**Comfort asking about abortion**	Are you comfortable asking respondents questions about abortion?	Yes, completely<br>Somewhat<br>No	-	Binary variable coded as 1 if interviewer answered 'yes completely' and 0 if they answered 'somewhat' or 'no'

For peer review only

Appendix 2. Characteristics of respondents and communities by country (weighted)

Respondent characteristics	Côte d'Ivoire (n=2798)		Rajasthan, India (n=5915)		Nigeria (n=11,303)	
	N	%	N	%	N	%
**Mean age** (SE)	28.5 (0.21)		29.1 (0.15)		29.1 (0.13)	
**Education**						
No formal schooling	1,282	45%	2,212	37%	2,389	18%
Primary school	733	26%	1,424	24%	1,931	15%
Secondary school	626	23%	1,509	26%	5,039	47%
Higher education	154	6%	770	14%	1,944	20%
**Marital status**						
Currently married	1,187	41%	4,547	77%	7,069	59%
Living with partner	622	24%	9	0%	414	5%
Divorced or widowed	129	4%	153	3%	528	5%
Never married	859	31%	1,188	20%	3,288	31%
**Parity**						
Ever given birth	2,080	74%	4,082	69%	7,456	65%
Mean number of births (SE)	2.5 (0.12)		1.9 (0.04)		2.3 (0.06)	
Previous PMA participant	387	23%	1,281	25%	1,884	23%
**Community characteristics**						
Urban	1,768	62%	1,503	35%	5,664	57%
Rural	1,096	38%	4,561	65%	5,875	43%
Abortion care facility in community	2,129	76%	1,624	27%	5,602	50%
Mean monthly facility-reported number of abortions, per community with abortion facility (SE)	2.7 (0.09)		12.7 (0.51)		5.9 (0.11)	

Abbreviations: SE = Standard Error.

For peer review only

Appendix 3: Full multilevel random intercept logit model (Model 2) for the odds of reporting removing a pregnancy, adjusted for interviewer, interview, respondent and community characteristics

	Côte d'Ivoire		Rajasthan, India		Nigeria	
	OR	[95% CI]	OR	[95% CI]	OR	[95% CI]
**Interviewer characteristics**						
Number of respondents	1.00	[0.98;1.01]	0.99	[0.97;1.01]	**0.99**	**[0.98;1.00]**
Interviewer age	0.99	[0.96;1.02]	1.02	[0.99;1.06]		
Married (vs not married)	1.17	[0.84;1.64]	0.73	[0.39;1.34]		
Has children (vs no children)	***1.33***	***[0.96;1.83]***	1.52	[0.85;2.74]		
Technical / Uni grad (vs secondary)	***1.47***	***[0.95;2.29]***	1.08	[0.73;1.59]		
Existing staff (vs new staff)	1.34	[0.52;3.44]	1.11	[0.66;1.87]		
Previous survey experience (v none)	1.36	[0.82;2.26]	1.01	[0.62;1.64]		
Very comfortable asking about abortion (vs somewhat/not comfortable)	***1.88***	***[0.9;3.9]***	1.55	[0.45;5.34]		
**Interview Characteristics**						
Very well or well acquainted	Ref		Ref		Ref	
Not well acquainted	1.22	[0.28;5.22]	**0.68**	**[0.49;0.96]**	**0.75**	**[0.58;0.97]**
Not acquainted	1.67	[0.45;6.17]	0.91	[0.57;1.45]	0.85	[0.65;1.13]
Language of interview						
French / Hindi / Hausa	Ref		Ref		Ref	
Baoule / English / English	**3.11**	**[2.00;4.83]**	0.77	[0.08;6.97]	**1.59**	**[1.19;2.14]**
Yacouba / Other / Igbo	0.54	[0.15;1.93]	1.54	[0.69;3.47]	1.13	[0.72;1.78]
Attie / - / Yoruba	0.75	[0.28;2.02]			**1.94**	**[1.16;3.25]**
Dioula / - / Pidgin	**0.26**	**[0.14;0.48]**			**1.57**	**[0.99;2.47]**
Lobi / - / Other	***0.17***	***[0.02;1.35]***			1.61	[0.73;3.55]
Other / - / -	0.67	[0.27;1.72]				
**Respondent Characteristics**						
Age	**1.35**	**[1.22;1.50]**	**1.40**	**[1.21;1.61]**	**1.46**	**[1.38;1.56]**
Age squared	**1.00**	**[0.99;1.00]**	**0.99**	**[0.99;1.00]**	**0.99**	**[0.99;1.00]**
No formal schooling	Ref		Ref		Ref	
Primary school	**1.96**	**[1.48;2.60]**	**2.01**	**[1.48;2.72]**	**1.62**	**[1.21;2.16]**
Secondary school	**2.10**	**[1.50;2.92]**	**1.46**	**[1.00;2.14]**	**2.19**	**[1.63;2.94]**
Higher education	1.31	[0.8;2.150]	1.49	[0.92;2.41]	**1.94**	**[1.39;2.7]**
Married	Ref		Ref		Ref	
Living with partner	**2.85**	**[2.12;3.83]**	1.33	[0.14;13.0]	**2.75**	**[2.07;3.65]**
Divorced or widowed	**2.25**	**[1.39;3.63]**	0.94	[0.47;1.89]	**1.44**	**[1.11;1.87]**
Never married	**3.66**	**[2.54;5.27]**	**0.14**	**[0.04;0.51]**	**1.30**	**[1.01;1.67]**
Ever given birth (vs never)	**3.63**	**[2.41;5.46]**	**2.76**	**[1.49;5.13]**	**1.39**	**[1.09;1.79]**
Number of birth events	**0.92**	**[0.85;1.00]**	1.08	[0.97;1.19]	***0.96***	***[0.92;1.01]***
Lowest wealth quintile	Ref		Ref		Ref	
Lower quintile	1.36	[0.90;2.05]	0.81	[0.51;1.29]	**1.44**	**[1.10;1.90]**
Middle quintile	1.23	[0.78;1.95]	1.06	[0.67;1.66]	***1.33***	***[0.96;1.84]***
Higher quintile	***1.61***	***[0.97;2.64]***	0.88	[0.54;1.43]	**1.50**	**[1.07;2.11]**
Highest quintile	**1.88**	**[1.12;3.15]**	1.07	[0.64;1.78]	**1.75**	**[1.22;2.52]**
Previous PMA respondent (vs not)	1.26	[0.90;1.75]	0.82	[0.62;1.10]	0.91	[0.74;1.10]
**Community Characteristics**						
Region or state	0.99	[0.97;1.01]	0.99	[0.97;1.01]	1.01	[0.94;1.08]
Rural (vs urban)	0.99	[0.68;1.46]	**0.57**	**[0.36;0.91]**	0.77	[0.56;1.05]
Monthly number of abortions per community (mean, facility-reported)	0.99	[0.95;1.04]	1.00	[0.97;1.02]	**0.97**	**[0.94;0.99]**
Abortion care facility in community (vs none)	0.75	[0.55;1.03]	1.42	[0.90;2.26]	**1.36**	**[1.04;1.78]**

For peer review only

**Intra-Interviewer Correlation**	**0.01**	**0.15**	**0.22**

Notes: Coefficients in bold are significant at the <0.05 level, coefficients in bold and italics are significant at the <0.10 level. Interviewer characteristics were unavailable for Nigeria. OR = odds ratio. CI = confidence intervals.

For peer review only

Appendix 4. Multilevel logit models with interviewer effects for PMA survey outcomes

	Côte d'Ivoire				Rajasthan, India				Nigeria			
	Level 2 variance	IIC	95% CI	LR test P value	Level 2 variance	IIC	95% CI	LR test P value	Level 2 variance	IIC	95% CI	LR test P value
**Abortion-related survey questions**												
**Ever removed a pregnancy**												
Null model	0.60	**0.15**	[0.10;0.23]		0.81	**0.20**	[0.14;0.28]		1.86	**0.36**	[0.31;0.42]	
Model 1	0.24	**0.07**	[0.03;0.13]	<0.001	0.74	**0.18**	[0.12;0.26]	<0.001	1.05	**0.24**	[0.20;0.30]	<0.001
Model 2	0.03	**0.01**	[0.00;0.11]	<0.001	0.60	**0.15**	[0.10;0.24]	0.15	0.94	**0.22**	[0.18;0.27]	<0.001
**Ever regulated a period**												
Null model	1.63	**0.33**	[0.23;0.45]		0.74	**0.18**	[0.10;0.32]		1.95	**0.37**	[0.31;0.44]	
Model 1	1.56	**0.32**	[0.22;0.45]	<0.001	0.67	**0.17**	[0.08;0.31]	<0.001	1.55	**0.32**	[0.26;0.39	<0.001
Model 2	1.10	**0.25**	[0.15;0.38]	0.03	0.45	**0.12**	[0.05;0.27]	0.08	1.51	**0.32**	[0.25;0.38]	0.30
**Confidante removed pregnancy**												
Null model	0.40	**0.11**	[0.06;0.18]		1.17	**0.26**	[0.20;0.34]		1.33	**0.29**	[0.24;0.35]	
Model 1	0.34	**0.09**	[0.05;0.17]	<0.001	1.15	**0.26**	[0.20;0.33]	<0.001	1.10	**0.25**	[0.20;0.31]	<0.001
Model 2	0.15	**0.04**	[0.01;0.12]	0.01	0.99	**0.23**	[0.17;0.31]	0.18	1.01	**0.23**	[0.19;0.29]	<0.001
**Less stigmatised survey questions**												
**Current contraceptive user**												
Null model	0.40	**0.11**	[0.07;0.17]		0.35	**0.10**	[0.07;0.13]		1.15	**0.26**	[0.22;0.31]	
Model 1	0.27	**0.08**	[0.04;0.13]	<0.001	0.83	**0.20**	[0.16;0.26]	<0.001	0.95	**0.22**	[0.18;0.27]	<0.001
Model 2	0.17	**0.05**	[0.02;0.10]	<0.001	0.73	**0.18**	[0.14;0.24]	0.07	0.92	**0.22**	[0.18;0.26]	0.07
**Ever given birth**												
Null model	0.43	**0.11**	[0.07;0.17]		0.06	**0.02**	[0.01;0.03]		0.37	**0.10**	[0.08;0.13]	
Model 1*	0.09	**0.03**	[0.01;0.12]	<0.001	0.10	**0.03**	[0.01;0.10]	<0.001	0.25	**0.07**	[0.05;0.11]	<0.001
Model 2	0.02	**0.01**	[0.00;0.00]	0.19	0.02	**0.00**	[0.00;0.85]	0.06	0.23	**0.06**	[0.04;0.10]	<0.001
**Currently pregnant**												
Null model	0.06	**0.02**	[0.00;0.00]		0.19	**0.05**	[0.02;0.12]		0.27	**0.08**	[0.05;0.11]	
Model 1**	0.00	**0.00**	[0.00;0.00]	<0.001	0.15	**0.04**	[0.01;0.13]	<0.001	0.17	**0.05**	[0.03;0.08]	<0.001
Model 2	0.00	**0.00**	[0.00;0.00]	0.29	0.11	**0.03**	[0.01;0.14]	0.41	0.17	**0.05**	[0.03;0.08]	0.08

Notes: Model 1 includes respondent variables (age, education, marital status, ever given birth, number of birth events, wealth, previous PMA experience) and community variables (region or state, rural / urban status, presence of facility providing abortion, monthly average number of abortions reported by facilities in community).
Model 2 includes respondent, community, and interviewer / interview-related variables (interviewer age, marital status, children, education, previous PMA staff, previous survey experience, comfort asking questions about abortion, respondent-interviewer familiarity and survey language).
*Excluding parity variables. **Excludes respondent marital status for Rajasthan. IIC = intra-interviewer correlation. CI = confidence intervals. LR test = Likelihood ratio test.

For peer review only

STROBE Statement—Checklist of items that should be included in reports of ***cross-sectional studies***

	Item No	Recommendation	Page No
**Title and abstract**	1	(*a*) Indicate the study's design with a commonly used term in the title or the abstract	Page 1
		(*b*) Provide in the abstract an informative and balanced summary of what was done and what was found	Page 1
**Introduction**			
Background/rationale	2	Explain the scientific background and rationale for the investigation being reported	Page 3-4
Objectives	3	State specific objectives, including any prespecified hypotheses	Page 5
**Methods**			
Study design	4	Present key elements of study design early in the paper	Page 5-6,7
Setting	5	Describe the setting, locations, and relevant dates, including periods of recruitment, exposure, follow-up, and data collection	Page 5-6
Participants	6	(*a*) Give the eligibility criteria, and the sources and methods of selection of participants	Page 5-6
Variables	7	Clearly define all outcomes, exposures, predictors, potential confounders, and effect modifiers. Give diagnostic criteria, if applicable	Page 6-7, Appendix 1
Data sources/ measurement	8*	For each variable of interest, give sources of data and details of methods of assessment (measurement). Describe comparability of assessment methods if there is more than one group	Page 6-7, Appendix 1
Bias	9	Describe any efforts to address potential sources of bias	Page 8
Study size	10	Explain how the study size was arrived at	Page 5-6
Quantitative variables	11	Explain how quantitative variables were handled in the analyses. If applicable, describe which groupings were chosen and why	Page 6-7, Appendix 1
Statistical methods	12	(*a*) Describe all statistical methods, including those used to control for confounding	Page 8
		(*b*) Describe any methods used to examine subgroups and interactions	Page 8
		(*c*) Explain how missing data were addressed	Page 8
		(*d*) If applicable, describe analytical methods taking account of sampling strategy	Page 8
		(*e*) Describe any sensitivity analyses	Page 8
**Results**			
Participants	13*	(a) Report numbers of individuals at each stage of study—eg numbers potentially eligible, examined for eligibility, confirmed eligible, included in the study, completing follow-up, and analysed	n/a
		(b) Give reasons for non-participation at each stage	n/a
		(c) Consider use of a flow diagram	n/a
Descriptive data	14*	(a) Give characteristics of study participants (eg demographic, clinical, social) and information on exposures and potential confounders	Page 19/20 Tables 1 & 2
		(b) Indicate number of participants with missing data for each variable of interest	Page 19/20 Tables 1 & 2
Outcome data	15*	Report numbers of outcome events or summary measures	Page 20, Table 2

For peer review only

For peer review only - http://bmjopen.bmj.com/site/about/guidelines.xhtml

Main results	16	(*a*) Give unadjusted estimates and, if applicable, confounder-adjusted estimates and their precision (eg, 95% confidence interval). Make clear which confounders were adjusted for and why they were included	Page 23, Table 3
		(*b*) Report category boundaries when continuous variables were categorized	N/A
		(*c*) If relevant, consider translating estimates of relative risk into absolute risk for a meaningful time period	N/A
Other analyses	17	Report other analyses done—eg analyses of subgroups and interactions, and sensitivity analyses	Page 8
**Discussion**			
Key results	18	Summarise key results with reference to study objectives	Page 10
Limitations	19	Discuss limitations of the study, taking into account sources of potential bias or imprecision. Discuss both direction and magnitude of any potential bias	Page 10-11
Interpretation	20	Give a cautious overall interpretation of results considering objectives, limitations, multiplicity of analyses, results from similar studies, and other relevant evidence	Page 10-11
Generalisability	21	Discuss the generalisability (external validity) of the study results	Page 11
**Other information**			
Funding	22	Give the source of funding and the role of the funders for the present study and, if applicable, for the original study on which the present article is based	Page 13

*Give information separately for exposed and unexposed groups.

Note: An Explanation and Elaboration article discusses each checklist item and gives methodological background and published examples of transparent reporting. The STROBE checklist is best used in conjunction with this article (freely available on the Web sites of PLoS Medicine at http://www.plosmedicine.org/, Annals of Internal Medicine at http://www.annals.org/, and Epidemiology at http://www.epidem.com/). Information on the STROBE Initiative is available at www.strobe-statement.org.

For peer review only
